# Individual Differences in Responsiveness to Acupuncture: An Exploratory Survey of Practitioner Opinion

**DOI:** 10.3390/medicines5030085

**Published:** 2018-08-06

**Authors:** David F. Mayor, Lara S. McClure, J. Helgi Clayton McClure

**Affiliations:** 1 Department of Allied Health Professions and Midwifery, School of Health and Social Work, University of Hertfordshire, Hatfield AL10 9AB, UK; 2 Northern College of Acupuncture, York YO1 6LJ, UK; LaraMcClure@chinese-medicine.co.uk (L.S.M.); helgi.claytonmcclure@oxon.org (J.H.C.M.)

**Keywords:** acupuncture, responsiveness, practitioner survey, patient characteristics, thematic analysis, Shannon entropy

## Abstract

**Background:** Previous research has considered the impact of personal and situational factors on treatment responses. This article documents the first phase of a four-stage project on patient characteristics that may influence responsiveness to acupuncture treatment, reporting results from an exploratory practitioner survey. **Methods:** Acupuncture practitioners from various medical professions were recruited through professional organisations to complete an online survey about their demographics and attitudes as well as 60 questions on specific factors that might influence treatment. They gave categorical (“Yes”, “No”, and “Don’t know”) and free-text responses. Quantitative and qualitative (thematic) analyses were then conducted. **Results:** There were more affirmative than negative or uncertain responses overall. Certain characteristics, including ability to relax, exercise and diet, were most often considered relevant. Younger and male practitioners were more likely to respond negatively. Limited support was found for groupings between characteristics. Qualitative data provide explanatory depth. Response fatigue was evident over the course of the survey. **Conclusions:** Targeting and reminders may benefit uptake when conducting survey research. Practitioner characteristics influence their appreciation of patient characteristics. Factors consistently viewed as important included ability to relax, exercise and diet. Acupuncture practitioners may benefit from additional training in certain areas. Surveys may produce more informative results if reduced in length and complexity.

## 1. Introduction

Outcomes from acupuncture treatment have been considered to depend on many interacting factors, including—among others—the condition treated, treatment parameters (acupuncture points and procedures used), setting, practitioner experience, characteristics and attitude, the patient–practitioner relationship, advice given, co-interventions, conditioning (e.g., from treatment repetition) and expectation [1,2,3,4,5,6,7,8,9,10,11].

What about the patient in the acupuncture scenario? The respected German-born British pioneering medical acupuncture practitioner Felix Mann (1931–2014) introduced the term “strong reactor” to describe a subset of patients who respond particularly strongly to acupuncture [12], with very rapid alleviation of their symptoms, although he was not able to define such patients otherwise than by observing that they seemed more likely to be artistic or inclined toward religious belief than less strong reactors. Similar to Mann, some British proponents of medical acupuncture such as Anthony Campbell and Peter Baldry have noted that strong reactors are also often “good responders” to acupuncture [1,13], with Campbell following a suggestion by Johnson et al. [14] concerning transcutaneous electrical nerve stimulation (TENS) that strong reactors could be people whose central nervous system, including the limbic system, is particularly sensitive to sensory stimulation [13]. Among the present authors, in D.F.M.’s experience over 36 years of clinical practice and in line with Campbell’s findings [15], some patients have certainly seemed to respond well to acupuncture and benefit a great deal from receiving treatment, whereas others have appeared to respond less well, or even poorly, and benefit less from treatment—almost regardless of what that treatment is.

A central aim of this paper is to explore what practitioners consider as possible individual characteristics, attitudes and experiences that may contribute to someone being a “good” or “poor” responder. We make no claims about what actually are the factors that influence responsiveness, but contribute to a framework for assessing the impact of patient characteristics on treatment outcomes. This may ultimately assist in developing models of patient response tendencies, such as that of the “good responder” [1,13]. Further aims are to present some preliminary findings concerning such general questions as: “Are some patients more receptive to acupuncture in general, with a better, faster or more enduring response to treatment than others?”, “Can acupuncture responders be consistently categorised as ‘good’, ‘average’ or ‘poor’, or does this vary?”, or “Is placebo responsiveness considered materially to contribute to acupuncture responsiveness?”

Investigating such characteristics and questions is potentially important for any therapeutic intervention, and certainly not acupuncture alone, but they are rarely addressed in the literature except in a very limited way. In recent years, for example, genetic polymorphism (genotyping) has been investigated for its effects on treatment outcome in several fields—particularly in hepatology [16,17]—with a view to developing more personalised approaches to treatment or improving outcome prediction. Genomic correlates of the placebo response (the “placebome”) have also been proposed [18,19], and such an approach has been used, if sparsely, in the field of acupuncture. Thus, response to acupuncture for smoking cessation was found in one Korean study to vary with genetic polymorphisms [20], and using serotonin transporter polymorphism techniques has been proposed as a method of guiding individualised treatment of irritable bowel syndrome in Chinese medicine [21]. More generally, genetic polymorphism has been shown to regulate the default mode network (DMN) in the brain, and so to regulate response to acupuncture stimulation [22]. In a very different approach, poor response to acupuncture used as an adjuvant treatment for In Vitro Fertilisation was found to be more likely in those with high peak levels of follicular stimulating hormone, longer histories of infertility and worse sperm morphology [23], but a quick PubMed search revealed no other studies that looked explicitly at the effects of such factors on treatment outcome.

Furthermore, genetic testing is highly technical and costly, and offers little insight to the clinician in daily practice. Similarly, although there are many studies on the endocrinology [24] and neurochemistry of acupuncture [25,26], again few are relevant in everyday practice. Therefore, a simpler, more accessible questionnaire-based research protocol was developed in an attempt to assess whether there might be any simple answers to the question “who responds well to acupuncture?” without recourse to complex and costly biomarkers (a similar approach, using psychometric data to evaluate the effects of patient personality on their response to placebo acupuncture, was explored by Kaptchuk and colleagues [27]). Such a protocol might seem not just simple but naïve and simplistic to those used to large-scale scientific research, but its feasibility and limited funding requirements recommended it for the current exploratory work. Development was by D.F.M. in association with a small focus group comprising six other experienced acupuncturists and researchers (one of whom previously published a paper about the effects of attachment style on response to acupuncture [28]), one neurofeedback practitioner and a retired medical doctor/government advisor.

To contextualise this paper, the protocol, still in development, is in four phases:

A A survey of UK acupuncture practitioners to find why members of the profession think some patients respond better to acupuncture than others.
B Use of self-report personality scales with around 100 participants who have taken part in acupuncture-related studies conducted at the University of Hertfordshire since 2011, to assess whether there are any meaningful associations between these traits and their electroencephalography (EEG), heart rate variability (HRV) and outcomes data already collected.
C A (blinded) retrospective survey of acupuncture teaching clinic patients who have responded either well or poorly to acupuncture, using a variety of short, established self-report personality questionnaires to determine whether any of the traits assessed have a bearing on outcome.
D A prospective study of patients using a smaller selection of self-report questionnaires (based on Phases A–C above), together with outcome measures such as the Measure Yourself Medical Outcome Profile (MYMOP) and perhaps a multiple measure of mood change similar to those developed and piloted by D.F.M. and other collaborators [29,30].

Phase A of the project is presented here. The survey process is described in Section 2.1 and Section 2.2; a quantitative analysis of the categorical “Yes”/“No”/“Don’t know” survey responses is undertaken in Section 3.2.3, with a nested qualitative analysis of the free-text responses in Section 3.3; the main conclusions are given in Section 5. The survey questions and the actual data gathered are provided in the Supplementary Materials (http://www.mdpi.com/2305-6320/5/3/85/s1).

Data for Phase B have now been collected and are currently in process of analysis. Phases C and D are still in the planning stage. In preparation, some small pilot studies have been undertaken to test the use of selected self-report questionnaires for the retrospective self-assessment of acupuncture’s effectiveness and prospective assessment of mood changes in response to electroacupuncture in a teaching situation [30,31], with a further pilot study still in process.

## 2. Materials and Methods

Ethics approval was obtained from the University of Hertfordshire for Phases A and B of this project (Protocol HSK/SF/UH/02930, 3 August 2017).

Initially, the acupuncture literature as well as general literature on factors having an impact on health and wellbeing was reviewed to locate possible individual characteristics and experiences that might possibly affect treatment responsiveness and for which validated self-report questionnaires exist. Based on this review, the pilot studies mentioned above and prior experience of running acupuncture surveys [32,33], a survey was developed in consultation with the focus group, members of which were recruited informally from among those with an interest in the survey subject matter. The survey was then trialled by the group prior to launch. Several revisions were made at each stage of this process until the survey was finally considered ready for use.

### 2.1. The Survey

The final online version of the survey, hosted by Jisc (Bristol Online Surveys), was launched on 16 October 2017 and closed after 19 weeks on 28 February 2018. Before respondents could take the survey, they were informed about its purpose, origins and how long it was likely to take them to complete, and then asked for their consent to continue [34]. The 13-page survey included three initial questions about the respondents themselves, four about their acupuncture training, professional affiliation and practice, and one (Q9) asking whether—before becoming aware of the survey—they had ever considered that patient characteristics (such as temperament or personality traits) might affect treatment response.

Then followed the main part of the survey, consisting of 60 questions on particular patient characteristics, attitudes or experience that could contribute to how well (or poorly) they respond to acupuncture, derived from the literature on what can affect health (Q10.1 to Q12.18). Respondents were asked which of the listed suggestions they currently considered might have an important effect on treatment response (“What you THINK NOW”). They were also asked whether, in their own clinical practice, they thought at the time of giving a treatment or shortly afterwards that any of the listed suggestions had an impact on an individual patient’s response to acupuncture (“What you THOUGHT THEN”). Answers could be “Yes”, “No” or “Don’t know” (“Yes”, “No” and blank for the “THEN” responses). NOW and THEN questions were separately included deliberately to encompass responses based on both current knowledge (or opinion) and historical experience, which could relate to a single, specific past experience or on the practitioner’s career experience as a whole. Explanations were provided for terms used in 10 of the questions which might be unfamiliar to those taking the survey. The survey also included 69 free-text boxes where respondents could choose to qualify their categorical answers with further comments.

In addition, respondents were asked further questions about their views on consistency of response to acupuncture treatment (Q13), short-term and long-term response to acupuncture treatment (Q14), three questions on their own response to acupuncture (Q15), two about their patients’ response to acupuncture (Q16–17), and a final two about conditions they consider as responding particularly well or badly to acupuncture (Q18–19).

The survey questions and responses are summarised in the Supplementary Materials.

### 2.2. Recruitment

**Figure 1:**
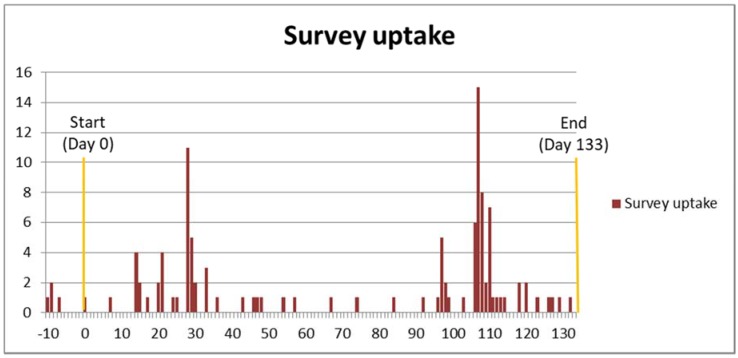
Survey uptake over time, showing the effects of the reminder sent five weeks before closure.

Information about the survey was sent to all the major (and some less established) UK professional acupuncture associations (Table 1), and also to selected training institutions whose graduates tended to become members of these associations. A template was provided for them to inform their members/graduates about the survey using various methods—newsletters (printed and/or electronic), emails, member forums and social media. No incentives were offered for completing the survey. Survey uptake was monitored by D.F.M.; reminders were sent to the associations, and by them to their members, some five weeks before the survey closed. Clearly, the reminder appears to have been effective (Figure 1); it also seems possible that members of the smaller associations (apart from the Chinese Medical Institute and Register (CMIR)) were more likely to take the survey; although the correlation between membership numbers and percentages of these that took the survey was not significant (*r_s_* = −0.464, *p* = 0.294), the trendline of a scatterplot for the two remaining sets of six numbers—without the CMIR data—shows a tantalisingly clear power distribution, so that overall numbers were low (median percentage of membership 1.43%, interquartile range 0.43–1.94%).

### 2.3. Analysis

For the most part, data were not normally distributed when tested using the Shapiro–Wilk test and for skewness and kurtosis. Analysis was therefore conducted using non-parametric methods in SPSS (v23) and Excel (2010). Randolph’s free-marginal multi-rater *kappa* was calculated using the online calculator (at http://justusrandolph.net/kappa/), and Shannon entropy using the simple Excel-based method described in a previous study [30].

## 3. Results

### 3.1. The Respondents

In total, 114 people completed the survey, including four members of the focus group before the official survey start date (the results for these four are not included in the Supplementary Materials). One of these was not an acupuncture practitioner, one other respondent was also not an acupuncturist (although trained), and one completed the survey prior to training.

There were 79 female and 35 male respondents, the median age for both genders being 52 (interquartile ranges, IQR, being 42–57 and 48–64, respectively).

Most respondents (83) considered themselves primarily as acupuncturists or practitioners of traditional Chinese medicine (TCM), as against only seven as medical doctors, six as physiotherapists or nurses (including midwives) and two as chiropractors. There were more male than female medical doctors and chiropractors among the respondents, although not significantly.

Median ages for the acupuncturists/TCM practitioners, chiropractors and other professionals who completed the survey were very similar (52, IQR 46–58). However, those for medical doctors were much greater (63, IQR 59.5–69.5), and those for physiotherapists much less (35.5, IQR 30.5–48).

There was a strong correlation between respondents’ age and how long they had used acupuncture in clinical practice (Spearman’s rank correlation coefficient *rho* or *r_s_* = 0.535, *p* < 0.0001); this was more marked for men (*r_s_* = 0.712) than for women (*r_s_* = 0.438).

Respondents had used acupuncture for a median of ten years (IQR 5–19), with medical doctors using it for longest (20 years, IQR 13.5–35) and “other” practitioners for the shortest time (3 years, IQR 1.5–10). 

Different styles of acupuncture treatment were used, which could be categorised as more “traditional” (e.g., TCM, Five-Element, Japanese or “Tung’s style”), used by 88 respondents, and more “modern” or “Western” (e.g., Western medical, Trigger point or “Formula”), used by 23. Those using more Western styles tended to be older (median age 56, IQR 47–63) than the traditionalists (median age 52, IQR 42.25–57), although not significantly. There was also a higher proportion of males among those using Western styles (72.7%) than those using more traditional methods (65.2%), but again this difference was not significant.

Of those who classed themselves as good responders to acupuncture (Q15), 34 were older than the median age for the sample, and 26 younger, whereas this was reversed for those who considered themselves only as average responders (20 being younger and 15 older than the median age). Those who considered themselves good responders were also more likely than those who thought of themselves as average responders to assess their own patients as good responders (Q16): 65.6% of the former described 80% or 100% of their patients as good responders, as against only 43.2% of the latter (with good responder practitioners correspondingly less likely to describe their patients as poor responders (Q17)). However, none of these differences were significant.

### 3.2. The Questions

#### 3.2.1. Questions Requiring “Yes” or “No” Responses—An Overview

In total, 124 questions required a “Yes” or “No” response. The 60 questions on particular patient characteristics, attitudes or experience could be answered as “What you THINK NOW” and “What you THOUGHT THEN”, with “Yes”, “No” or “Don’t know” responses, and a further four more general questions could be answered simply with “Yes” or “No”. A full list of responses for these questions is given in Appendix A.

The 60 specific questions were generally answered in the affirmative, both when considered by respondent and by question (Table 2). This is consistent with the fact that 96 respondents (84.2%) stated that they had previously considered that patient characteristics (such as their temperament or personality traits) might affect their response to treatment (Q9), and that 70 respondents (61.4%) stated their believe that that some patients respond consistently well or poorly to acupuncture, almost regardless of other factors such as the condition treated or their state of health at the time (Q13).

There were strong correlations between “Yes” (NOW) and “Yes” (THEN) responses (*r_s_* = 0.655, *p* < 0.0001), between “No” (NOW) and “No” (THEN) responses (*r_s_* = 0.609, *p* < 0.0001), and between “Yes” and “No” (NOW) responses (*r_s_* = −0.685, *p* < 0.0001). There was also a slightly weaker correlation between “Don’t know” (NOW) and “Don’t know” (THEN) responses (*r_s_* = 0.487, *p* < 0.0001), but none between “Yes” and “No” (THEN) responses (*r_s_* = 0.001, *p* = 0.989). 

#### 3.2.2. On Specific Questions Requiring “Yes” or “No” Responses

Of the 60 questions in the main part of the survey, those in the upper and lower deciles for numbers of “Yes” and “No” scores are shown in Table 3 (with numbers of responses in each case).

**Figure 2:**
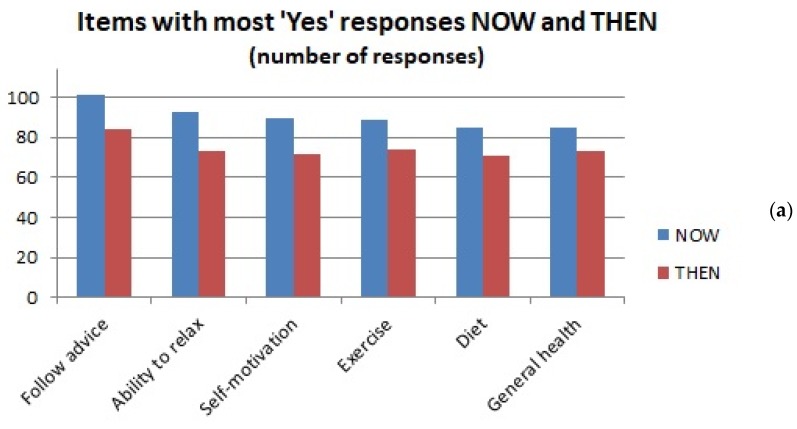

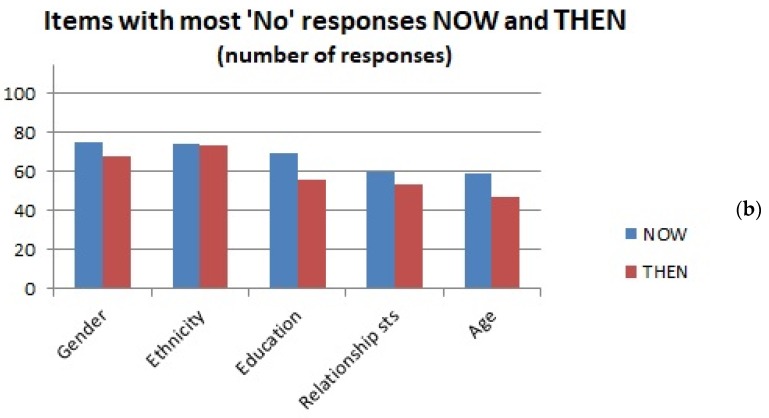
Salient findings for the “Yes” and “No” responses in Table 3. (**a**): Items with most “Yes” responses; (**b**): Items with most “No” responses.

A graphical representation of salient findings for “Yes” and “No” responses is given in Figure 2.

As for the “Yes” and “No” responses in general, there is thus considerable agreement between the patient characteristics perceived to affect treatment outcome in past clinical practice and at the time of responding.

Attributes most consistently considered to affect response are *willingness to follow advice*, *self-motivation*, *general health status*, *ability to relax*, *exercise* and *diet*. Those most consistently considered *not* to affect response are *patient age*, *gender*, *ethnicity*, *education* and *relationship status* (“Relnship status” in Table 3).

Respondents were least likely to hazard a guess for the somewhat abstract characteristics of *alexithymia* and *central sensitisation* (“Central sensitisn” in Table 3), as well as *gender issues*, *child poverty*, *character when young* and *TCM pattern*.

#### 3.2.3. Respondent Characteristics and Yes/No Responses

##### Age and Years in Practice

Other than a small negative correlation between respondent age and the number of “No” (THEN) answers given (*r_s_* = −0.215, *p* = 0.022), there were no particular correlations between numbers of “Yes” or “No” responses and either respondents’ ages or years in practice. However, if the sample was divided into those younger and older than the median age (52), a Mann–Whitney test showed significant differences of “No” and “Don’t know” (THEN) responses between older and younger respondents (“No”: *U* = 903.0, *p* = 0.004; “Don’t know”: *U* = 928.5, *p* = 0.006). Younger respondents (THEN) were more likely to answer “No”, and older to answer “Don’t know” (the same was true for the NOW responses considered together, but these differences were nonsignificant).

These patterns in age differences were significant for responses to ten of the 60 questions (three NOW and seven THEN), with a Kruskal–Wallis test indicating *p* values < 0.01 for three of them: Birth and prenatal experience (NOW) (*p* = 0.008, *χ*^2^ = 9.57), Housing situation (THEN) (*p* = 0.002, *χ*^2^ = 12.19) and Work situation (NOW) (*p* = 0.005, *χ*^2^ = 10.54).

There were also eight significant differences among responses to specific questions with years in practice (*p* values < 0.05), those in practice for longer being more likely to consider central sensitisation (both NOW and THEN) a relevant factor, for example, but also less likely to consider optimism (both NOW and THEN) as having an impact on treatment outcome.

##### Gender

Table 4 illustrates differences in response frequencies by respondent gender.

A Mann–Whitney test indicated that significantly more women than men gave “Yes” (THEN) responses (two-tailed significance, *U* = 941.5, *p* = 0.007), but this difference was not significant for the “Yes” (NOW) responses (*p* = 0.071). Conversely, men provided significantly more “Don’t know” (THEN) responses (i.e., did not answer these questions) than women (*U* = 1000.0, *p* = 0.018). 

**Figure 3:**
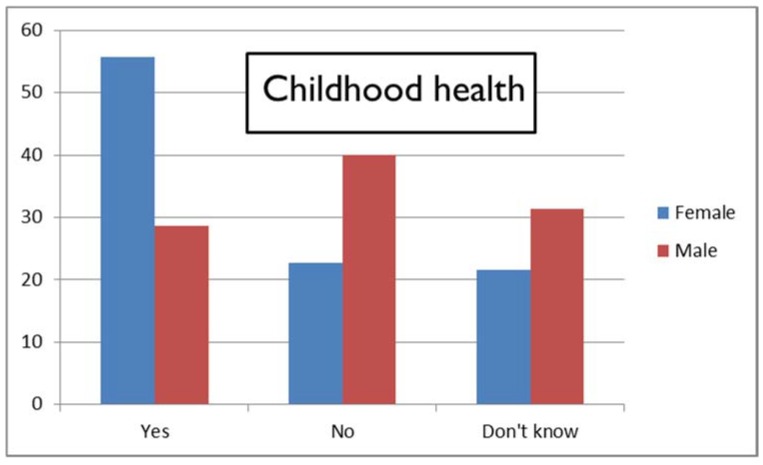
Percentages of female and male respondents giving “Yes”, “No”, and “Don’t know” responses regarding the item on *childhood health* (NOW responses).

One particular question exemplifies this difference between female and male respondents, as shown in Figure 3: most women considered NOW that childhood health could impact treatment response, whereas men generally did not (Pearson’s *χ*^2^ = 7.30, *p* = 0.026).

A very similar result was found for *family health in the patient’s childhood* (Pearson’s *χ*^2^ = 7.26, *p* = 0.027). In all there were 11 questions (three NOW, seven THEN) for which female and male responses differed significantly. As in Table 4 for the 60 questions considered together, more males than females replied “Don’t know” to these individual questions.

##### Main Profession

The medical doctors (who were mostly male) gave fewer “Yes” responses than the remainder of the respondents, although a Kruskal–Wallis test showed these difference not to be significant. When ratios of numbers of “Yes” to “No” scores are considered, acupuncturists appear least likely to consider that individual patient characteristics may affect outcome. In contrast, physiotherapists and nurses (including midwives) showed the highest ratio of numbers of “Yes” to “No” scores (both NOW and THEN) (Table 5).

Taking only those professions showing the most extreme NOW ratios from the table above (acupuncturists and physiotherapists), responses for the individual questions were examined. Mostly Yes-to-No count ratios were of a similar order and in the same direction for both professions; they were only significantly different and definitively in the opposite direction for one question: religious beliefs or practices (*p* = 0.005, *χ*^2^ = 10.41). All the physiotherapists considered this might be a factor in treatment response, whereas only 33.8% of the acupuncturists did.

##### Professional Association Membership

BAcC and ATCM members, although all acupuncturists, responded in different ways to this survey. The median ages of those belonging to the two associations were comparable, but, whereas there were equal numbers of female and male ATCM respondents, there were many more female than male BAcC respondents (47 versus 9). The former recorded the most “No” (THEN) responses of any association members (median per member 18.5, IQR 4–28), and the latter the least (median 4.5, IQR 0.8–9.3), a significant difference (*p* values of between 0.014 and 0.043 using the Mann–Whitney test, depending on analysis of multiple-affiliated respondents). Correspondingly, median Yes-to-No count ratios THEN were highest for ATCM members (although ratios NOW were highest for AACP members). Otherwise, association membership did not appear to have a significant effect on the total number of “Yes”, “No” or “Don’t know” responses (NOW or THEN). Lowest Yes-to-No count ratios (whether NOW or THEN) were for those stating they belonged to another association than those listed.

Analysing only the THEN responses for ATCM and BAcC members using the same procedure as for acupuncturists and physiotherapists in the previous section, again Yes-to-No count ratios were mostly of a similar order and in the same direction for both groups.

##### Style of Practice

There were no significant differences in numbers of “Yes” or “No” responses (either NOW or THEN) with style of practice—more “traditional” or more “Western”—although the former tended to respond more definitively (whether with “Yes” or “No”) and the latter more with “Don’t know” (both NOW and THEN).

##### Prior Opinion That Patient Characteristics Might Affect Treatment Response

In total, 113 respondents answered the question on whether in the past they had considered that patient characteristics might affect treatment response, 97 of them in the affirmative.

Those who held the prior opinion that patient characteristics might affect their response to treatment were more likely to respond “Yes” to both NOW (*U* = 366.0, *p* = 0.001) and THEN (*U* = 480.0, *p* = 0.015) questions than those who did not, and were correspondingly less likely to give “No” responses.

#### 3.2.4. Associations between the 60 Main Questions in the Survey

There is no unique or perfectly precise way of classifying the 60 questions on individual characteristics that might affect treatment outcome, and the list itself is not exhaustive. An initial attempt was made to group them under 14 different headings, such as early life, social/financial, behavioural attitudes, and so forth. A confirmatory cluster analysis was then undertaken on the basis of these groupings, but did not yield useful results. Therefore, an alternative approach was used, assessing associations among “Yes”, “No” and “Don’t know” responses for the different questions using *χ^2^* tests.

These showed strong associations (low *p* values) between “Yes”, “No” and “Don’t know” responses for some measures within the anticipated groupings. However, not all of these supported the groupings initially proposed, so that these were adjusted in an attempt to maximise significance of the associations, resulting in the final groupings shown in Appendix A. Even so, some groupings have more explanatory value than others.

The limited convergence of the *χ*^2^ (or phi or Cramer’s V) measures of association and the results of the hierarchical cluster analysis for NOW (only 19 of 100 tested associations appearing at the first stage of the agglomeration process) led to the decision not to investigate such a relationship for the THEN questions.

Some of the *χ^2^* groupings appeared as consecutive questions in the original survey, so this may have swayed respondents to answer similarly. To test this, associations between eight consecutive but unrelated questions were also explored (see Appendix A). The results of this test indicate that just because questions follow each other consecutively, they are not necessarily answered in the same manner.

As for internal consistency, Cronbach’s alpha was 0.930 for the NOW responses, and 0.981 for the THEN responses. Removal of individual items did not greatly affect these values—for NOW, alpha ranged from 0.928 to 0.931, and, for THEN, from 0.981 to 0.982.

#### 3.2.5. Agreement between Respondents, Variability and Variance of Responses

Three methods were used to assess the consistency of responses across respondents. Firstly, Randolph’s free-marginal multi-rater *kappa* [35,36] was calculated for both NOW and THEN questions and used to derive a measure of “overall agreement” between participants on each question. The questions for which *kappa* exceeded 0.4 (considered at least a moderate level of agreement [37]) are tabulated in Appendix A. Secondly, Shannon Entropy (SE) values were calculated for each question, both NOW and THEN. SE is a measure of the inherent “informativity” (uncertainty or randomness of information) in a given string of data, where higher values indicate more uncertainty or informativity [38]; we have used this approach in previous pilot studies [30,31]. Randolph’s *kappa* and SE are corollaries, since they respectively measure agreement and variability in a data sample. This was reflected by extremely strong negative correlations between *kappa* (or overall agreement) and SE across all questions in both NOW and THEN responses (*r_s_* ≤ −0.998, *p* < 0.0001). This pattern is shown graphically in Appendix A. A third measure, a non-parametric version of coefficient of variance (CV), defined as inter-quartile range divided by the median and multiplied by 100, produced significant correlations in the variance of both “Yes” and “No” responses between NOW and THEN (*r_s_* = 0.870 and 0.632, *p* < 0.0001 and *p* = 0.006), but was omitted from further analysis due to its lack of convergence with *kappa* or SE (correlations ns).

When individual questions were considered, there was considerable overlap with the previous analysis of “Yes”, “No” and “Don’t know” response frequencies outlined in Table 3 (Section 3.2.2). Only two questions with lowest inter-rater agreement (or greatest SE) appear in that table, both under “DK” responses. Thus, our analysis of respondent agreement (or variability) tallies with our previous analysis of consistency of response by question.

#### 3.2.6. Patterns in Survey Completion Assessed from Numbers of “Yes”, “No” and “Don’t know” Answers

**Figure 4:**
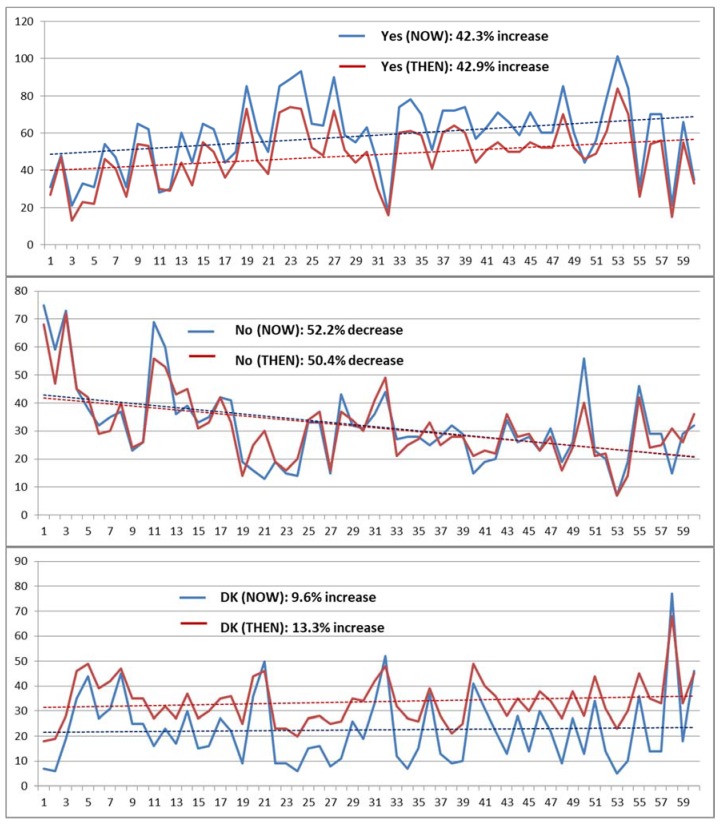
Changes in numbers of “Yes”, “No” and “Don’t know” responses to the 60 main questions over the course of the survey completing them.

There are clear trends in the numbers of “Yes” and “No” responses to the 60 main questions over the course of the survey, with considerable agreement between the NOW and THEN responses, but rather less pronounced trends in the numbers of “Don’t know” responses (Figure 4).

When variability values were computed for four consecutive quarters of the main questions (1–15, 16–30, etc.), a similar pattern was observed, with 95.3% and 80.0% overall increases in the *kappa* values for NOW and THEN questions between the first and last quarter, but generally smaller corresponding changes in SE and non-parametric CV (npCV).

### 3.3. Thematic Analysis of Free-Text Responses

**Figure 5:**
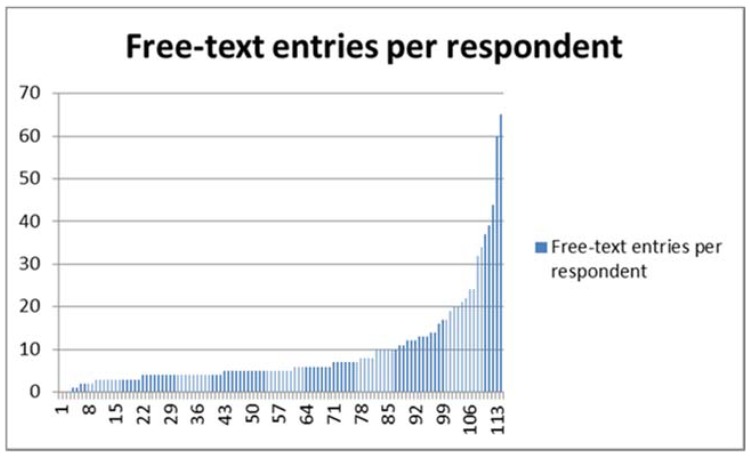
Free-text entries across all parts of the survey, shown in ascending order by respondent.

Respondents providing qualitative responses are self-selecting participants; by first ticking a box to indicate understanding of a question’s underlying premise (“Yes”, “No”, “Don’t know”), and then deciding at each free-text prompt whether to provide further information, respondents declare buy-in at two levels. Simple counts of the number of responses made to each question (Figure 5) show where this second level of buy-in is strongest.

The maximum number of free-text responses to any survey item was 106, and the minimum 1 (median 10, IQR 7–13). The maximum number of free-text responses for any respondent was 65, and the minimum 0 (median 5, IQR 4–10), as shown in Figure 5. In what follows, respondents are identified by number (from 1 to 114), and the questions to which they respond by their alphanumeric code (from 9.a to 20); thus, “11.4.c/63” refers to Respondent 63’s answer to question 11.4.c.

Questions that elicited the most free-text responses were the general questions (9, 15, 18 and 19), with fewest in the areas of *resilience* (3 responses), *self-regulation* (3), *suggestibility* (3) and *extraversion–introversion* (1).

It is perhaps unsurprising that Question 9, the first and most fundamental question—”Before you knew about this survey, did you ever consider that patient characteristics (such as their temperament or personality traits) might affect their response to treatment?”—elicited the largest number of responses (94/114), indicating a perceived need for qualification of the key issue at the outset. Some respondents pick out a single characteristic to highlight here—for example, “calm” patients (9.a/68) are deemed more responsive, “anxious or hostile” (9.a/66) or “depressed and negative” patients (9.a/71) less so. Variation in response intensity is allowed, with some patients identified as “super responders” (9.a/100).

This questionnaire deliberately uses the distinction between the general and the particular, asking respondents to both consider their general opinion at the time of responding, and give deliberately separate consideration to their opinion of a specific patient interaction in the past. A response of “Don’t know” is only possible in the “NOW” answers, demanding concrete judgment of the evoked historical event.

Some characteristics are in themselves symptoms or medical conditions and could be the reason for treatment being sought—e.g., anxiety, depression. One respondent highlights this with a repeat response—”depends if this is a symptom/being treated” (verbatim at 11.4.c/63, 11.5.c/63, 11.19.c/63).

Respondents’ comments about patient response are predicated on their own understanding of what “response” means in this context. Notions of “response” are unpacked in the introductory matter for the survey, with a range of meanings suggested encompassing improvement in symptoms and changes in wellbeing and quality of life. However, no formal definition of a “good” or “poor” response or responder was provided.

Texts were extracted from the optional free-text fields to a spreadsheet for thematic analysis. Whilst these texts are researcher-instigated data, they have been generated independently by respondents without the researcher present and so remain subjective, “re-presentations of reality rather than simply true or false” [39] (p. 275). These occupy a middle ground within the continuum of qualitative data between the “gold standard” interview and “naturally occurring” texts such as transcribed conversations and fieldnotes [40] (pp. 669–670). We allow that unique statements have as much potential importance as frequently-found words [41].

A relatively informal approach to analysis was appropriate, as “the qualitative text analysis is not at the core of the research but instead is in a subsidiary or complementary role” [40] (p. 670). Immersion in the data led to emergence of themes, illustrated below by means of in vivo quotes. Mason’s tripartite levels of analysis were used to arrange the themes at literal, interpretive, and reflexive levels [42] (p. 180) to gain understanding of what acupuncturists think about their patients’ identity as “responders” to acupuncture. These themes apply axially across the different question areas [43].

#### 3.3.1. Literal Analysis

Literal analysis assigns literal meaning to statements. Many emergent literal themes (indicated here in italics) in the present data directly stem from specific topic areas established in the survey.

Questions about the impact of *gender* and *age* on response elicit high levels of response (23 responses to each, the second-highest count amongst all the free-text responses). There is broad consensus that women are better responders than men, and younger patients better than older (for multiple examples, see the data repository at http://www.mdpi.com/2305-6320/5/3/85/s1). Respondent 99 is a lone voice opining that neither gender nor age affects response (10.1.c/99 and 10.2.c/99).

By contrast, there is no consensus on the effect of *ethnicity* on response, and only 11 responses are made, perhaps indicating that responder fatigue is already in play by this point in the survey [44].

Of the 15 free-text responses offered in relation to *work*, seven mention *stress*.

*Childhood* aspects are important for a small set of respondents, but as the factors listed multiply, answers become less specific, with certain respondents opting for generic “Ibid”-type responses (see, for example, 10.6.c/45 and 10.6.c/70, and Respondent 5’s generic approach to all answers). Others rephrase ideas already expressed in connection with the age question, perhaps indicating some theoretical saturation and respondent fatigue as well as consistency of views. Some respondents may not have been able to make subtle distinctions between overlapping categories such as *self-motivation* and *optimism*, *self-efficacy* and *resilience*—in some cases declaredly so, despite the provision of glossary information in the survey itself.

An important outcome of the present survey is an update to the acupuncture profession’s perceived *scope of expertise* as represented by the list of conditions that these practitioners find respond well to acupuncture. There is strong consensus around certain conditions, including musculoskeletal problems, anxiety, depression, irritable bowel syndrome (IBS), headaches and chronic pain conditions (see data repository for full details). There is clear overlap between this list and the “effectiveness gaps” identified in UK GP care over a decade ago [45], and also with those conditions identified as having the strongest published evidence for the effectiveness of acupuncture [46].

#### 3.3.2. Interpretive Analysis

Interpretive analysis assigns contextualised meaning to statements. Emergent themes are again indicated here in italics.

Paired polarised positions of *belief* and *scepticism* are given as baseline patient characteristics for some respondents, with general consensus that belief improves response, although the premise is also contested—”Belief is irrelevant” (9.a/11), “non believers can get the same benefit as well!” (9.a/21). Belief finds 69 direct mentions in the data and can also be discerned in “softened” forms such as expectation of positive outcome (9.a/5) and willingness to engage (9.a/34). Scepticism (24 mentions) is depicted as an undesirable starting-point characteristic which acupuncture treatment can shift—”attitude changes with improvement in their condition” (9.a/114). A reinforcing cycle of improved outcomes with increased buy-in to the system responsible is perceived, and acupuncture’s capacity to potentiate *positive change* and enhance *self-efficacy* is acknowledged as it is in current literature [47]. Respondent 43 (amongst others) is repeatedly concerned with acupuncture’s impact on individuals’ *ability to cope*, emphasising that this is a more important factor than changes to symptoms, in particular pain levels (see, for e.g., 10.12.c/43 and 10.13.c/43).

*Placebo* finds frequent mention both as a pejorative and as a self-evident absolute—”Positivity helps the placebo response” (9.a/40). Individual respondents’ understanding of and political attitude towards the term influences response. Debate on the elusive nature of a plausible inert placebo for use in clinical trials of acupuncture is not new [48]. Superficial needling or the application of non-penetrative devices to acupuncture points is recognised to stimulate these points in a manner equitable to a lower dose of the same treatment [49,50] and subsequent research in this area has led to the development of pragmatic trial models which assess the effectiveness of acupuncture treatment against active comparators in ecologically valid settings [51,52]. Awareness of this context is discernible in many responses here.

Appearing in relation to hypochondria (now more formally known as “illness anxiety disorder”), and elsewhere, the notion of *sick role* is important to several respondents, with reference to patients who “may not want to get well” (11.20.c/37), “have an identity with being ill” (11.20.c/109) or even “a victim complex” (9.a/77). Notions of belief are again in play—”some people are clearly stuck in the ‘sick role’ mindset and therefore physically get better but don’t believe they are” (9.a/30).

The experience of appropriately-placed experts is being deliberately sought by this survey, so evocation of the *theoretical knowledge base* particular to the profession is perhaps inevitable: “more metal types can be more resistant” (9.a/29). *Specialisms* appear too, with Respondent 74 focusing on pregnancy and Respondent 43 on chronic pain. Information being elicited is to some degree held as *self-evident*—”anyone qualified would know this as part of their studies if not common sense” (9.a/27), “pretty obvious” (10.8.c/45).

*Individual patient diagnosis* emerges as a priority with regard to response/non-response, with consensus found across many areas and specifically with regard to depression (see, for example, 11.18.c/4 and 11.18.c/18) that the innate and unique characteristics of each individual patient are the deciding factor with regard to response level. There are repeated pleas for the diagnostic specificity of the medicine (such as Respondent 63’s answers above) and acknowledgement of the non-specific treatment effects known to accompany it [47,53,54].

#### 3.3.3. Reflexive Analysis

Reflexive analysis assigns reflexive meaning to statements. In this context, this analysis considers the data in relation to the acupuncture profession as a whole. Emergent themes are again indicated here in italics.

*Belief* reappears in the form of witness-type statements when respondents are asked about themselves as responders, with 18 counts of “I believe” as well as mentions of “faith”. Pseudo-religious language may stem from the acupuncture profession’s self-identification as distinct, special and “other”. This question also prompts *ego*-driven statements: “since I started practising I’ve become quite adept at predicting who will respond well based on the initial consultation” (9.a/82).

Consideration of *Belief* and *scepticism* may have roots in the dichotomy-driven nature of Traditional Chinese Medicine theory. Thirty-two responses detail specific TCM/5-element diagnostic patterns, again indicating recourse to the *theoretical knowledge base* of the profession. Evocation of the particulars of a medicine system about which respondents are passionate indicates a desire to establish professional *credibility* and *expertise*. This is in places wielded with discernible *defensiveness* against the survey instrument, the questions, and the non-responsive *sick role* patient, who is “never satisfied” (11.20.c/30) and “not quite engaged into getting well” (9.a/39). Focus on *scepticism* may indicate a defensiveness on behalf of a medicine seen as contested—”there is always an element of you having to prove yourself continually” (9.a/49): “Some patients seem to be self sabotaging and come convinced that acupuncture does not work” (9.a/54). *Judgmental language* permeates the discourse of certain individuals (e.g., Respondent 43).

The response trajectory of specific individual *respondent personalities* can be traced through the data set. Respondent 45, a 69 year old male, gives 65 free-text answers, the maximum number of free-text responses by any respondent (minimum = 1, median = 5). Respondent 45 establishes a distinctive “voice” early on, taking issue with the fundamental premise of the endeavour—“characteristics such as temperament or personality traits seem a little fuzzy to me” (9.a/45), and succumbing to petulant exasperation later on—“I repeat: a patient with a healthy, balanced qi will respond better to acupuncture treatment and vice versa” (10.8.c/45). Respondent 45’s desire to return to a repeated universal answer reiterates his perception of the universality of the medicine he practises. When asked about factors like housing and work, he consistently holds that these things have relevance only insofar as they “affect the state of the person’s qi” (verbatim at 10.14.c/45 and 10.15.c/45).

45 personifies the survey instrument, adopting a tone of debate with the researcher, who is assigned an assumed medicopolitical stance—“all actions (external, internal, emotional, etc.) that affect the state of our qi, blood, jing, shen, etc. will have an effect on how we function, both in the language of CM or that of biomedical science, which you seem to favour” (11.1.c/45).

## 4. Discussion

This study resulted in several unexpected and useful findings. The first of these (Figure 1) is that it may clearly be useful, when survey uptake is flagging, to remind potential respondents of its existence. Another finding on recruitment (Table 1) is that members of the smaller professional associations were more likely to take the survey, suggesting that personal contact with association staff may be an important factor in increasing uptake. Even so, only 114 people completed the survey out of more than 12,000 potential respondents, an uptake of less than one percent. However, in this age of internet marketing, low response rates are not uncommon. As another organiser of acupuncture surveys has stated, “The number of acupuncture practitioners who respond to electronic surveys tends to be disappointing, but reasons are unclear why this is so” [55]. Personal contact may always be useful, but equally may bias responses.

Considerably more women than men took the survey (Section 3.1), as in our previous survey on electroacupuncture usage, where this was particularly true for UK respondents [33]. Again, as in the earlier survey, male respondents outnumbered females among medical doctors and chiropractors (osteopaths in the earlier survey), although numbers were too small for this to reach significance. Medical doctors tended to be older, and physiotherapists younger, than the majority of survey respondents. It is possible that those doctors that did respond had an interest in the subject matter of the survey because of early contact or training with the doyens of medical acupuncture who first proposed the notions of “strong reactor” and “good responder”. Acupuncturists often enter the profession as a second career, but for physiotherapists acupuncture is usually an add-on to their first main career, so it makes sense that physiotherapists would be the youngest group.

A high proportion (84.2%) of respondents stated that in the past (before they knew about this survey) they had considered that patient characteristics might affect their response to treatment (Section 3.2.1). Correspondingly, the vast majority of individual THEN and NOW questions were given “Yes” answers. This suggests that responses to the main body of the survey were generally made in a manner congruent with existing practitioner attitudes, rather than being arbitrary. The qualitative evidence indicating clear conceptual understanding of relevant patient characteristics supports this, also suggesting that the respondents answered with reference to theoretical frameworks central to their practice (Section 3.3). The fact that respondents generally did not make suggestions for additional attributes that ought to be included suggests that the “long-list” of 60 questions was quite comprehensive in terms of the factors readily encountered in a treatment context.

The overall positive correlation between “NOW” and “THEN” responses may be driven by various factors, including a simple tendency to tick the same box twice while completing the survey (response perseveration). Although it is of course possible that previous clinical experience heavily shapes later attitudes (i.e., a true similarity between what they thought THEN and NOW), it is difficult to conclude this from the current data alone, since the clinical context(s) on the basis of which practitioners made their THEN judgments were not controlled. More recently qualified practitioners (50 of 114 within the last 10 years) may have made these judgments in a very different manner from those who have had both greater subsequent experience and longer to forget!

The specific questions that elicited most and fewest responses (Section 3.2.2) merit particular attention. Those characteristics/behaviours which were most often considered to impact on treatment outcome (Table 3) included what acupuncture author Bob Flaws once called “the three frees”—relaxation, diet and exercise—but also *self-motivation*, *willingness to follow advice* given, *general state of health* and *openness* to new experiences. In the HRV literature on acupuncture, parasympathetic activation or an improvement in sympathovagal balance is often found to result from acupuncture treatment [56,57,58], so a pre-existing ability to relax could facilitate this. The remaining items are all quite straightforward and easy to interpret in the context of acupuncture, where for instance patients less open to new experiences are presumably less likely to procure treatment in the first place. 

The items judged least often to impact outcome included age and gender. Statements are often found in the paediatric acupuncture literature [59,60] to the effect that children tend to respond particularly well, but at the other end of the age spectrum there appears to be little information available on whether elderly patients respond better or worse than those in their middle years, despite the existence of many studies on the use of acupuncture for conditions of old age. This is in sharp contrast with the qualitative findings, where many of the free-text comments regarding patient age and gender emphasised their general significance for treatment response—with younger and female patients perceived as likely to respond better to acupuncture. It is important to qualify this apparently contradictory result by noting that, although both were relatively popular items for free-text commentary, only 23 qualitative responses were given regarding patient age and gender across 114 respondents.

There was considerable agreement that relationship status per se does not have much effect on responsiveness to acupuncture, although arguments could be made that a lack of close satisfactory relationship(s) is likely to affect general health as well as the practitioner-patient relationship [28]. Other general characteristics such as level of education and ethnicity were also thought to have little effect on treatment outcome. It is certainly of interest that 50% of respondents were of the opinion that scepticism does not affect outcome—implying a lack of consensus from a quantitative perspective. The qualitative data help to clarify this by showing distinct schools of thought about the impact of *belief* in the treatment technique, with some mentioning a positive impact, while most respondents acknowledged that patients show different levels of scepticism—whether it is influential on treatment outcomes.

Items about which respondents were least certain included *alexithymia* and *central sensitisation* (which may be unfamiliar or difficult to grasp), *gender issues* (which have only become salient *zeitgeist* issues fairly recently), and *TCM* or *Five-element* pattern. It was unexpected that this last item should appear here, as both models differentiate diagnostic categories by their expected response to treatment: dampness and phlegm are considered relatively difficult to treat in TCM, for instance [61]. Theoretical constructs such as the three major blocks to treatment, namely “possession”, “husband/wife imbalance” and “aggressive energy” [62], further imply that choice of theoretical framework may have an impact on how patient characteristics are expected to influence treatment response.

All in all, only around 20 items appear in Table 3; there was no clear convergence of opinion among respondents about the remaining two-thirds of the questions. The groupings for which the lowest proportions of items were included in the table are shown in Table A20 in Appendix A. Those groupings which produced least consensus (from which fewest items are therefore included in Table 3) were 4 (Trauma), 6 (Beliefs/attitudes), 5 (Social/financial) and particularly 12 (Psychological attitudes 1), whereas those producing the clearest consensus were 1 (Demographic 1), 2 (Demographic 2), 7 (Lifestyle) and 15 (Behavioural attitudes).

It may well be the case that groupings where respondents did not show clear consensus include items which really do have little impact on treatment outcome, and those where they concurred, items which do have an impact. However, it is also possible that acupuncture practitioners in general may not be trained to have sufficient awareness of the impacts on health and recovery of such things as social and financial situations [63] and psychological attitude [64,65]. This gap in knowledge may be pertinent for those who design acupuncture training courses (especially those for continuing professional education).

There was some variation in response patterns between groups of practitioners (Section 3.2.3). For example, younger respondents (i.e., those earlier in their acupuncture careers) were more likely than their elders to discount particular patient characteristics as having a possible impact on treatment outcome, whereas older respondents (those likely to have had more experience of actual practice) were less decided (Age and Years in Practice). This may in part be due to changes in acupuncture education over the years, with younger/more recently trained practitioners placing more emphasis on core protocols and theory; older practitioners, on the other hand, may have had less information-rich instruction and so more of a desire to explore for themselves. However, such an interpretation is conjectural, and would need to be confirmed with further investigation. Furthermore, male respondents appeared slightly less inclined than female respondents to consider particular patient characteristics as having a possible impact on treatment outcome. This gender difference was significant for several specific questions (Gender). Medical doctors (who were mostly male) gave fewer “Yes” responses than the remainder of the respondents, physiotherapists and nurses more; physiotherapists were unanimous in their view that *religious beliefs or practices* might be a factor in treatment response, as against only a third of the acupuncturists (Main Profession); however, this could be a chance finding, the result of small sample size and asking a large number of questions, and should certainly not be taken to imply that physiotherapists have a fundamentally more positive view on the influence of religious beliefs.

The differences in survey responses between BAcC and ATCM members are intriguing (Professional Association Membership). In addition to the observed difference in gender profile in the two groups of respondents, it is worth noting that ATCM members are mostly TCM doctors trained in China. As already discussed, (Western) medical doctors tended to give fewer affirmative responses than non-doctors, but there is perhaps a greater tendency to conformity among Chinese than Western practitioners, which may have influenced their responses [66]. Style of practice (Style of Practice) and prior opinion that patient characteristics might affect treatment response (Prior Opinion That Patient Characteristics Might Affect Treatment Response) did not appear to affect survey responses in any striking or unexpected ways.

Different methods of grouping the questions were attempted (Section 3.2.4), from prima facie to cluster analysis to *χ*^2^ tests. A compromise set of 17 groupings, using three methods, was arrived at, and performed well (using *χ*^2^ tests) against a set of unrelated but consecutive questions in the survey, indicating that the groupings were not simply the result of perseveration of responses over strings of questions (Appendix A). When numbers of “Yes”, “No” and “Don’t know” responses for the groupings were calculated, as a generalisation, respondents appear to have considered Lifestyle, Stress/relaxation and Behavioural attitudes as having more of an impact on treatment outcomes than Early life, Attitudes to religion or nature, basic Demographics and some of the more complex (less behavioural) issues such as *attachment* or *central sensitisation*, which clearly did not strike a chord with many respondents.

This again perhaps suggests a lack of awareness of some less behavioural psychological perspectives on health among acupuncture practitioners.

Three measures of consistency of response were explored (Section 3.2.5), namely Randolph’s free-marginal multi-rater *kappa*, Shannon entropy (SE) and a novel non-parametric equivalent of the coefficient of variation (npCV). As expected from their complementary mathematical basis, *kappa* and SE values showed strong negative correlations.

Surprisingly, given its origin in information theory, SE has rarely been used in the analysis of questionnaire responses, with only two relevant citations found in PubMed [67,68], although the long-term dynamics of questionnaire mood responses have been studied using a similar measure, *approximate entropy* [69]. To our knowledge, this is the first time that an explicit association between SE and *kappa* has been reported, although given that the former is a measure of response variability and the latter of agreement, the negative correlation between them would be expected. Indeed, SE-based measures of consensus and dissention for Likert scale data have been suggested before [70,71], although they do not appear to have been used except by their originators. For both datasets (NOW and THEN), a *kappa* value of 0.4 corresponds to SE of 1.02; thus, an intermediate or better value of *kappa*, using Fleiss’s definitions, corresponds to a SE of 1.02 or less. Conversely, an SE of 1 corresponds to a *kappa* value of 0.41–0.42. This suggests a useful rule of thumb for interrogating questionnaire SE data, and further avenues for exploring relationships between other measures of inter-rater agreement and measures of entropy.

In contrast to the very strong inverse association between *kappa* and SE, correlations between npCV and *kappa* or SE were not significant. Thus, npCV measures a different construct from the other two methods. Further research will be needed to determine if it is useful in the context of analysing questionnaire or survey responses.

Survey fatigue is suggested by the trending of “Yes” and “No” responses over the course of the survey (Section 3.2.6), although numbers of “Don’t know” responses did not increase markedly, as can sometimes occur when respondents experience fatigue [72]. Increases in *kappa*, as well as decreases in SE and (for the most part) npCV, also suggest that participants were fatigued by the end of the survey, perhaps tending to fall into a pattern of repeating responses without giving each question due attention. 

This was indeed a long survey for people to take, and it is not known how many of those targeted started but did not complete it, or did not even contemplate taking the survey because the topic did not interest them. Many of the questions may have seemed irrelevant, strange or even bewildering to some acupuncture practitioners unfamiliar with the vast literature on other therapeutic approaches or the sociology of health. It may indeed be “a waste of time” (as one respondent put it) to try to determine whether individual characteristics or attributes can affect response to acupuncture in general, but results from our other pilot studies are encouraging, and the results of this survey, together with those from Phase B of this project, will be useful in designing Phase C before, we hope, moving on to a prospective study to investigate the question of whether good and poor responders to acupuncture can be predicted in advance from their psychometric or other data.

Looking at the qualitative arm alone, the existence of “good responders” appears confirmed by this survey of opinion—with some areas of consensus as to the nature of that “good response” and an understanding that it can vary in intensity.

The acupuncturists who responded to this survey appear to have clear concepts of the characteristics that contribute to their patients’ response to acupuncture treatment—although probably for a variety of reasons—and found a range of ways to link these characteristics to the core theoretical base of their medicine. Individual variation is a priority. Acupuncture treatment is characterised by these respondents as a collaborative endeavour which builds self-efficacy in a cycle of reinforcement and has an important preventative aspect. The agendas of both individuals and the whole of the profession they represent can be discerned in these texts [40] (p. 686).

## 5. Conclusions

The main conclusions from this study are:

1 If appropriate, reminders sent out a few weeks before a survey is closed could well increase uptake. Authors of acupuncture surveys should not expect enthusiastic uptake unless their survey is of particular relevance to their pool of potential respondents. In addition, members of smaller professional organisations may be more likely to respond than those of larger acupuncture associations.
2 Practitioner age and gender influence how they view the importance of patient characteristics, as do the practitioner’s main profession and potentially their own ethnicity.
3 Attributes most consistently reported to affect treatment outcome were diet, exercise and the ability to relax (Bob Flaws’ “Three frees”), together with general health, self-motivation and a willingness to follow advice.
4 However, a lack of awareness of more complex or difficult psychological and social issues may have skewed the current findings, obscuring the potential importance of some less obvious attributes.
5 Attempts to group characteristics according to item response patterns met with limited success, perhaps relating to the aforementioned skewing.
6 Survey fatigue was observed in terms of numbers of “Yes” and “No” responses, as well as changes in response variability, over the course of completing the survey.
7 Qualitative data may support different and subtler conclusions regarding acupuncturists’ appreciation of factors influencing their practice. A key example here is the varying views on belief and scepticism, which “fall through the net” of the quantitative arm of the study.

### Limitations

This is a relatively small survey with a relatively large number of questions. It is also not known to what extent responses represent those of the population of UK acupuncture practitioners as a whole, or, of course, whether the results of the survey are transferrable to other populations (e.g., chiropractors, or acupuncture practitioners in other countries). Furthermore, respondents were asked about some individual characteristics and attributes about which they may have had little prior knowledge. Results should therefore be interpreted with caution. In addition, qualitative responses were optional and in many cases not given—whereas the picture provided by the quantitative data appears more comprehensive.

A serious problem with the survey—as with any survey—is that the outcomes reflect the respondents’ own characteristics, attributes, and bias. The results should therefore be considered as representing trends in practitioner opinion—a first step towards further confirmatory research, and not necessarily as truly representative of patient characteristics and attributes that may underlie their responsiveness to acupuncture. Nonetheless, we hope that the results of this survey will help practitioners gain insight into their patients’ responses in the clinical setting.

It is also important to note that this paper does not establish true impacts on responsiveness to acupuncture, but it does not intend to; in fact, it is preliminary to other studies which will actually investigate the effects of a subset of the characteristics currently explored.

## Figures and Tables

**Table 1:** Acupuncture Associations and training institutions informed about the survey, showing percentage of association members who completed the survey.

Association/Institution	Membership *M*	Respondents *R*	*R*/*M* (%)
Acupuncture Association of Chartered Physiotherapists (AACP)	6000	5	0.08%
Acupuncture Society (AS)	1000	20	2.0%
Association of Traditional Chinese Medicine (ATCM)	700	10	1.43%
British Academy of Western Medical Acupuncture (BAWMA)	150	12	8.00%
British Acupuncture Council (BAcC)	3000	56	1.87%
British Medical Acupuncture Society (BMAS)	2300	10	0.43%
Chinese Medical Institute and Register (CMIR)	240	1	0.42%
College of Chinese Medicine (CCM)		unknown	n/a
College of Integrated Chinese Medicine (CICM)		unknown	n/a
Northern College of Acupuncture (NCA)		unknown	n/a

Notes: Some membership numbers are taken from Mayor and Bovey 2016 [33], so percentage figures are indicative; 13 respondents stated they were members of “Other” associations, and 12 were concurrently members of two associations.

**Table 2:** Medians and IQRs of “Yes”, “No” and “Don’t know” (DK) answers to the 60 main survey questions, as percentages of total possible counts (60 by respondent, 114 by question).

“Now” or “Then”	How counted	“Yes” responses	“No” responses	“DK” responses
NOW	By respondent	53.3 (35.4–66.7)	25.0 (8.3–38.3)	15.0 (6.7–29.6)
	By question	53.1 (40.6–62.3)	25.4 (19.5–31.8)	16.2 (12.8–26.5)
THEN	By respondent	42.5 (18.8–63.3)	21.7 (5.0–44.2)	13.3 (1.7–47.9)
	By question	43.9 (35.3–49.8)	25.4 (20.8–33.1)	28.9 (23.7–34.2)

Note: “DK” responses THEN calculated as 60—(“Yes” + “No” counts).

**Table 3:** “Yes”, “No” and “Don’t know” (DK) responses in the upper and lower deciles for the 60 main survey questions (numbers of responses in brackets). Items shown in bold red type appear in the same deciles both NOW and THEN; those underlined appear in Most “Yes” responses and Least “No” responses (NOW or THEN), or vice versa.

	NOW		THEN	
	Most responses	Fewest responses	Most responses	Fewest responses
Yes	**Willing to follow advice** (101) **Able to relax** (93) **Self-motivated** (90) Exercise (89) Diet (85) General health (85) Openness (85)	Gender issues (17) **Ethnicity** (21) Alexithymia (21) Education (28) Relnship status (30) (and 4 others, ^a^ tied 31)	**Willing to follow advice** (84) **Exercise** (74) Able to relax (73) **General health** (73) **Self-motivated** (72) Diet (71)	**Ethnicity** (13) Alexithymia (15) **Gender issues** (16) Character when young (22) Birth/prenatal (23) Child poverty (26)
No	Gender (75) **Ethnicity** (74) **Education** (69) **Relnship status** (60) Age (59) Sceptical (57)	**Willing to follow advice** (9) Central sensitisn (13) Able to relax (14) Alexithymia (15) Psychotic (15) **Self-motivated** (15)	**Ethnicity** (73) Gender (68) Education (56) Relnship status (53) Gender issues (50) Age (47)	**Willing to follow advice** (7) Commitment (14) General health (14) Openness (16) **Self-motivated** (16) Exercise (17)
DK	Alexithymia (78) Gender issues (52) Central sensitsn (51) TCM pattern (47) Child poverty (45) Character when young (44)	Willing to follow advice (5) Able to relax (6) Age (6) Negativity (7) Gender (7) Self-motivated (8)		

^a^ *gender*, *character when young*, *childhood poverty* and *extraverted/introverted*. Two of these also appear under “Fewest responses (THEN)”.

**Table 4:** Median numbers of “Yes”, “No” and “Don’t know” responses for female and male respondents.

	NOW			THEN		
Gender	Yes	No	DK	Yes	No	DK
Female	33 (22–42)	15 (4–21)	10 (4–17)	31 * (15–40)	16 (3–27)	6 * (1–21)
Male	27 (20–36)	15 (6–30)	8 (3–21)	20 * (0–34)	8 (0–27)	19 * (2–59)

Note: “DK” responses THEN calculated as 60—(“Yes” + “No” counts); significant differences indicated with (*).

**Table 5:** Yes-to-No count ratios NOW and THEN, by profession, showing medians and IQRs.

Profession	Yes-To-No Count Ratios NOW	Yes-To-No Count Ratios THEN
Acupuncturists	1.7 (0.9–5.1)	1.4 (0.6–4.9)
Medical doctors	2.3 (2–2.7)	2.4 (1.3–2.5)
Physiotherapists	6.2 (1.8–17.6)	2.9 (1.7–4.1)
Nurses (and midwives)	4.8 (1.3–12.5)	3.5 (2.5–5.1)
Others	2.4 (1.5–2.9)	2.1 (1.0–3.1)

Note: Cases with no “No” responses are omitted, and as this only left one chiropractor, this category was removed from analysis.

**Figure A1:**
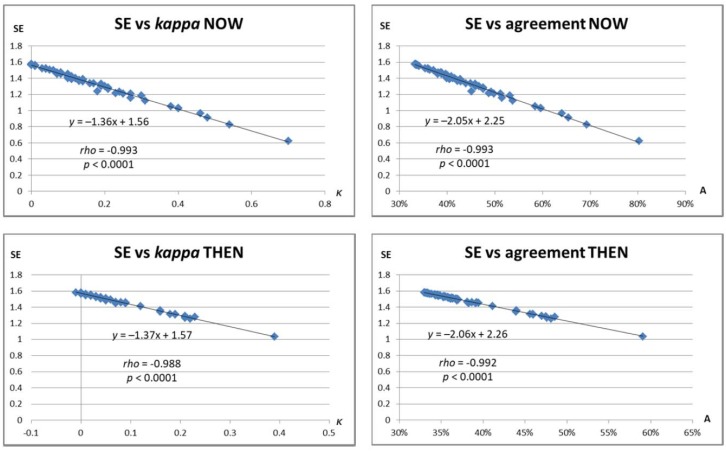
Scatter plots of Shannon entropy (SE) vs. free-marginal *kappa* and overall agreement for the 60 main survey questions.

## Supplementary Materials

The original data from the survey are available online, anonymised, as “Individual responsiveness to acupuncture-original data.xlsx” at http://www.mdpi.com/2305-6320/5/3/85/s1.

## Appendix A

Groupings of the 60 main questions in the survey.

Seventeen groupings were selected based on strong *χ^2^* associations (low *p* values) among “Yes”, “No” and “Don’t know” responses for the measures within them. However, despite adjustments to maximise *p* values, some questions remain uneasy bedfellows, such as the items in the “Trauma” grouping (Table A4), whereas others could be included in several different groupings (see, for example, the Note to Table A14, “Psychological characteristics”). The corresponding agglomeration stage for each NOW association was assessed from a dendrogram of a nine-stage confirmatory cluster analysis (using the hierarchical method, Ward’s method and squared Euclidean distances), and is indicated in each of Table A1, Table A2, Table A3, Table A4, Table A5, Table A6, Table A7, Table A8, Table A9, Table A10, Table A11, Table A12, Table A13, Table A14, Table A15, Table A16 and Table A17 by an asterisk and the stage number for each association.

**Table A1:** Demographic 1.

**NOW**	**Gender**	**Ethnicity**	**Education**
Gender		<0.0001 *^1^	<0.0001 *^2^
Ethnicity			<0.0001 *^2^
Education			
**THEN**	**Gender**	**Ethnicity**	**Education**
Gender		<0.0001	<0.0001
Ethnicity			<0.0001
Education			

*^1^: Agglomeration stage 1; *^2^: Agglomeration stage 2.

**Table A2:** Demographic 2.

**NOW**	**Age**	**General Health**
Age		0.001 *^3^
General health		
**THEN**	**Age**	**General Health**
Age		<0.0001
General health		

*^3^: Agglomeration stage 3.

**Table A3:** Early life.

**NOW**	**Birth/Prenatal**	**Characteristics**	**Health**	**Family Health**	**Poverty**
Birth/prenatal		<0.0001 *^3^	<0.0001 *^2^	<0.0001 *^2^	<0.0001 *^1^
Characteristics			<0.0001 *^3^	<0.0001 *^3^	<0.0001 *^3^
Health				<0.0001 *^1^	<0.0001 *^2^
Family health					<0.0001 *^2^
Poverty					
**THEN**	**Birth/Prenatal**	**Characteristics**	**Health**	**Family Health**	**Poverty**
Birth/prenatal		<0.0001	<0.0001	<0.0001	<0.0001
Characteristics			<0.0001	<0.0001	<0.0001
Health				<0.0001	<0.0001
Family health					<0.0001
Poverty					

*^1^: Agglomeration stage 1; *^2^: Agglomeration stage 2; *^3^: Agglomeration stage 3.

**Table A4:** Trauma.

**NOW**	**Early Trauma**	**Later Trauma**	**Past Invasive Med**
Early trauma		<0.0001 *^1^	0.023 *^4^
Later trauma			0.045 *^4^
Past invasive med			
**THEN**	**Early Trauma**	**Later Trauma**	**Past Invasive Med**
Early trauma		<0.0001	<0.0001
Later trauma			<0.0001
Past invasive med			

*^1^: Agglomeration stage 1; *^4^: Agglomeration stage 4.

**Table A5:** Social/financial.

**NOW**	**Relationship**	**Soc Support**	**Housing**	**Work**	**Finances**
Relationship		<0.0001 *^4^	<0.0001 *^4^	<0.0001 *^4^	<0.0001 *^4^
Soc support			<0.0001 *^2^	<0.0001 *^2^	<0.0001 *^2^
Housing				<0.0001 *^2^	<0.0001 *^2^
Work					<0.0001 *^1^
Finances					
**THEN**	**Relationship**	**Soc Support**	**Housing**	**Work**	**Finances**
Relationship		<0.0001	<0.0001	<0.0001	<0.0001
Soc support			<0.0001	<0.0001	<0.0001
Housing				<0.0001	<0.0001
Work					<0.0001
Finances					

*^1^: Agglomeration stage 1; *^2^: Agglomeration stage 2; *^4^: Agglomeration stage 4.

**Table A6:** Beliefs/attitudes.

**NOW**	**Religion**	**Nature/Technology**
Religion		<0.0001 *^2^
Nature/technology		
**THEN**	**Religion**	**Nature/Technology**
Religion		<0.0001
Nature/technology		

*^2^: Agglomeration stage 2.

**Table A7:** Lifestyle.

**NOW**	**Nutrition**	**Exercise**
Nutrition		<0.0001 *^1^
Exercise		
**THEN**	**Nutrition**	**Exercise**
Nutrition		<0.0001
Exercise		

*^1^: Agglomeration stage 1.

**Table A8:** Hypothalamic–pituitary–adrenal axis 1: Stress and relaxation.

**NOW**	**SensStress**	**Anxiety**	**RelaxAbil**
SensStress		<0.0001 *^1^	0.002 *^4^
Anxiety			<0.0001 *^4^
RelaxAbil			
**THEN**	**SensStress**	**Anxiety**	**RelaxAbil**
SensStress		<0.0001	<0.0001
Anxiety			<0.0001
RelaxAbil			

*^1^: Agglomeration stage 1; *^4^: Agglomeration stage 4.

**Table A9:** Hypothalamic–pituitary–adrenal axis 2: “Central sensitisation” and biochemistry.

**NOW**	**CentrSens**	**Neuroch**
CentrSens		<0.0001 *^1^
Neuroch		
**THEN**	**CentrSens**	**Neuroch**
CentrSens		<0.0001
Neuroch		

*^1^: Agglomeration stage 1.

**Table A10:** Somatisation (MUS), catastrophising, hypochondria and psychosis.

**NOW**	**MUS**	**Catastr**	**Hypoch**	**Psychosis ^a^**
MUS		<0.0001 *^4^	0.001 *^4^	0.002 *^4^
Catastr			<0.0001 *^1^	<0.0001 *^2^
Hypoch				<0.0001 *^2^
Psychosis				
**THEN**	**MUS**	**Catastr**	**Hypoch**	**Psychosis**
MUS		<0.0001	<0.0001	<0.0001
Catastr			<0.0001	<0.0001
Hypoch				<0.0001
Psychosis				

^a^ This may not appear the most likely grouping to include Psychosis, but, although it fits naturally in “Psychological characteristics” (#14 below), *p* is high at 0.010 for its association with Extravert/introvert (*p* is low, <0.0001, for the associations of Extravert/introvert with Depressive and Emotionally unstable). *^1^: Agglomeration stage 1; *^2^: Agglomeration stage 2; *^4^: Agglomeration stage 4.

**Table A11:** Attachment, addiction and identity.

**NOW**	**Attachment**	**Addiction**	**Doctor Shopping**	**Gender Issues**
Attachment		0.007 *^3^	<0.0001 *^3^	<0.0001 *^5^
Addiction			0.007 *^1^	0.001 *^5^
Doctor shopping				0.007 *^5^
Gender issues				
**THEN**	**Attachment**	**Addiction**	**Doctor shopping**	**Gender issues**
Attachment		<0.0001	<0.0001	<0.0001
Addiction			<0.0001	<0.0001
Doctor shopping				<0.0001
Gender issues				

*^1^: Agglomeration stage 1; *^3^: Agglomeration stage 3; *^5^: Agglomeration stage 5.

**Table A12:** Psychological attitudes 1.

**NOW**	**LifeSatis**	**Incontrol**	**S/Esteem**	**S/Efficacy**	**Resilience**	**Optim**	**Valency**	**S/Regul**
LifeSatis		<0.0001 *^1^	<0.0001 *^1^	<0.0001 *^5^	<0.0001 *^2^	<0.0001 *^4^	<0.0001 *^4^	<0.0001 *^5^
InControl			<0.0001 *^1^	<0.0001 *^5^	<0.0001 *^2^	<0.0001 *^4^	<0.0001 *^4^	<0.0001 *^5^
S/Esteem				<0.0001 *^5^	<0.0001 *^2^	<0.0001 *^4^	<0.0001 *^4^	<0.0001 *^5^
S/Efficacy					<0.0001 *^5^	<0.0001 *^5^	<0.0001 *^5^	<0.0001 *^1^
Resilience						<0.0001 *^4^	<0.0001 *^4^	<0.0001 *^5^
Optim							<0.0001 *^1^	<0.0001 *^5^
Valency								<0.0001 *^5^
S/Regul								
**THEN**	**LifeSatis**	**InControl**	**S/ESteem**	**S/Efficacy**	**Resilience**	**Optim**	**Valency**	**S/Regul**
LifeSatis		<0.0001	<0.0001	<0.0001	<0.0001	<0.0001	<0.0001	<0.0001
InControl			<0.0001	<0.0001	<0.0001	<0.0001	<0.0001	<0.0001
S/ESteem				<0.0001	<0.0001	<0.0001	<0.0001	<0.0001
S/Efficacy					<0.0001	<0.0001	<0.0001	<0.0001
Resilience						<0.0001	<0.0001	<0.0001
Optim							<0.0001	<0.0001
Valency								<0.0001
S/Regul								

*^1^: Agglomeration stage 1; *^2^: Agglomeration stage 2; *^4^: Agglomeration stage 4; *^5^: Agglomeration stage 5.

**Table A13:** Psychological attitudes 2, including placebo responsiveness.

**NOW**	**Defensive**	**Open**	**Suggestible**	**Sceptical**	**Trusting**	**Placebo**
Defensive		<0.0001 *^5^	<0.0001 *^1^	<0.0001 *^6^	<0.0001 *^4^	0.001 *^2^
Open			<0.0001 *^5^	0.005 *^5^	<0.0001 *^3^	0.003 *^5^
Suggestible				<0.0001 *^6^	<0.0001 *^4^	<0.0001 *^4^
Sceptical					<0.001 *^4^	<0.001 *^5^
Trusting						0.001 *^4^
Placebo						
**THEN**	**Defensive**	**Open**	**Suggestible**	**Sceptical**	**Trusting**	**Placebo**
Defensive		<0.0001	<0.0001	<0.0001	<0.0001	<0.0001
Open			<0.0001	<0.0001	<0.0001	<0.0001
Suggestible				<0.0001	<0.0001	<0.0001
Sceptical					<0.0001	<0.0001
Trusting						<0.0001
Placebo						

*^1^: Agglomeration stage 1; *^2^: Agglomeration stage 2; *^3^: Agglomeration stage 3; *^4^: Agglomeration stage 4; *^5^: Agglomeration stage 5; *^6^: Agglomeration stage 6.

**Table A14:** Psychological characteristics.

**NOW**	**Depressive**	**Unstable**	**Extrav/Introv**
Depressive		<0.0001 *^2^	<0.0001 *^5^
Unstable			<0.0001 *^5^
Extrav/Introv ^a^			
**THEN**	**Depressive**	**Unstable**	**Extrav/Introv**
Depressive		<0.0001	<0.0001
Unstable			<0.0001
Extrav/Introv ^a^			

a. Extravert/Introvert also demonstrated *p*-values < 0.0001 for groupings #11 and #13, for example. *^2^: Agglomeration stage 2; *^5^: Agglomeration stage 5.

**Table A15:** Behavioural attitudes.

**NOW**	**Self-Motivated**	**Follows Advice**	**Commitment**
Self-motivated		<0.0001 *^1^	<0.001 *^3^
Follows advice			<0.0001 *^3^
Commitment			
**THEN**	**Self-Motivated**	**Follows Advice**	**Commitment**
Self-motivated		<0.0001	<0.0001
Follows advice			<0.0001
Commitment			

*^1^: Agglomeration stage 1; *^3^: Agglomeration stage 3.

**Table A16:** Self-awareness.

**NOW**	**Bodily Aware**	**Emotionally Aware**	**Alexithymic**
Bodily aware		<0.0001 *^1^	<0.0001 *^5^
Emotionally aware			<0.0001 *^5^
Alexithymic			
**THEN**	**Bodily Aware**	**Emotionally Aware**	**Alexithymic**
Bodily aware		<0.0001	<0.0001
Emotionally aware			<0.0001
Alexithymic			

*^1^: Agglomeration stage 1; *^5^: Agglomeration stage 5.

**Table A17:** TCM.

NOW	*Qi* Strong/Weak	TCM/5E Diagnosis
*Qi* strong/weak		<0.0001
TCM/5E diagnosis		

Some of the *χ*^2^ groupings appeared as consecutive questions in the original survey, so this may have swayed respondents to answer similarly. To test this, associations between eight consecutive but unrelated questions were also explored (Table A18).

**Table A18:** Associations between consecutive survey questions from different groupings. Shown in **bold** are the *χ*^2^ associations from Table A17.

NOW	Religion	Nature/Tech	Health	Neurochem	Central Sens	Nutrition	Exercise	Ability to Relax
Religion		**<0.0001**	<0.0001	0.005	0.006	n.s.	n.s.	n.s.
Nature/Tech			<0.001	0.006	<0.001	0.034	n.s.	0.010
Health				<0.001	0.042	**<0.0001**	**<0.0001**	n.s.
Neurochem					**<0.0001**	n.s.	n.s.	n.s.
Central Sens						n.s.	n.s.	0.039
Nutrition							**<0.0001**	0.001
Exercise								0.014
Ability to Relax								

n.s.: not significant.

Thus, the *χ*^2^ associations between closely *consecutive* items does not necessarily imply that they are answered in the same manner.

Table A19 shows values of *kappa*, median SE and npCV for the 17 groupings. Table A20 shows the numbers of items from each grouping included and not included in Table 3.

**Table A19:** Values of *kappa*, median SE and npCV for the 17 groupings.

Grouping	NOW				THEN			
	*Kappa*	Median SE	npCV Yes	npCV No	*Kappa*	Median SE	npCV Yes	npCV No
1	0.22	1.29	18.97	4.05	0.14	1.36	32.14	12.50
2	0.29	1.13	27.41	51.28	0.14	1.37	21.31	54.10
3	0.01	1.57	45.45	7.89	0.02	1.54	69.23	29.27
4	0.13	1.40	3.03	23.08	0.04	1.52	4.72	23.08
5	0.09	1.43	40.38	24.00	0.02	1.55	41.45	15.91
6	0.03	1.54	6.38	2.38	0.00	1.58	11.11	13.16
7	0.43	1.00	1.71	8.57	0.21	1.30	1.37	5.56
8	0.33	1.22	14.38	32.14	0.14	1.41	13.11	16.00
9	0.11	1.40	9.91	10.34	0.01	1.55	8.43	9.09
10	0.15	1.37	14.18	21.79	0.04	1.51	10.85	38.64
11	0.06	1.46	42.38	41.94	0.03	1.52	59.15	47.83
12	0.14	1.38	14.45	14.29	0.03	1.54	12.87	19.40
13	0.17	1.43	28.75	32.35	0.07	1.51	4.85	25.00
14	0.15	1.31	30.28	31.03	0.06	1.47	28.33	26.79
15	0.52	0.91	8.79	36.67	0.26	1.29	9.59	32.14
16	0.22	1.30	35.71	25.86	0.09	1.50	37.27	14.00
17	0.07	1.48	30.69	4.92	0.02	1.54	25.00	16.13

**Table A20:** Numbers of items from each grouping included and not included in Table 3.

Grouping	Included	Not Included	% Included
1	3	0	100
2	2	0	100
3	3	2	60
4	0	3	0
5	1	4	20
6	0	2	0
7	2	0	100
8	1	2	33
9	1	1	50
10	1	3	25
11	1	3	25
12	1	7	12.5
13	2	4	33
14	1	2	33
15	3	0	100
16	1	2	33
17	1	1	50

Table A21 shows the individual questions in the upper and lower deciles for SE and *kappa* scores. Note that *all* questions for which *kappa* ≥ 0.4 are included in this table, and that *kappa* for none of the THEN questions reached 0.4.

**Table A21:** Survey questions in the upper and lower deciles for SE and *kappa* scores. As in Table 3, items shown in bold red type appear in the same deciles both NOW and THEN.

	Shannon Entropy (SE)			Free-Marginal *Kappa*	
	NOW	THEN		NOW	THEN
SE Q3	Birth/prenatal Char when young ^a^ **Child poverty** ^a^ **Housing** Attachment style Extravert/introvert	Religious beliefs Attitude to nature/t Self-efficacy Attachment style Self-regulation TCM diagnosis	*κ* Q1	Birth/prenatal **Character when young** ^a^ **Child poverty** ^a^ Housing Attachment style TCM diagnosis	Family health young Religious beliefs Attitude to nature/t Self-efficacy Self-regulation TCM diagnosis
SE Q1	General health ^b,d^ **Diet** ^b,d^ **Exercise** ^b,d^ **Ability to relax** ^b,d^ **Self-motived** ^b,d^ **Will follow advice** ^b,d^	**Ethnicity** ^c^ **General health** ^b^ **Exercise** ^b^ Ability to relax ^b^ **Self-motivated** ^b^ **Will follow advice** ^b^	*κ* Q3	**Diet** ^b,d^ **Exercise** ^b,d^ **Ability to relax** ^b,d^ **Self-motivated** ^b,d^ Openness ^b,d^ **Will follow advice** ^b,d^	**Ethnicity** ^c^ **General health** ^b^ **Exercise** ^b^ **Self-motivated** ^b^ **Will follow advice** ^b^ Commitment ^c^

Notes: ^a^ “DK” in Table 3; ^b^ “Yes” in Table 3; ^c^ “No” in Table 3; ^d^ *kappa* ≥ 0.4.

Comparing Table A21 and Table 3, there is considerable agreement between questions for which there was greatest inter-rater agreement (or lowest SE) and those for which most responses were “Yes” (either NOW or THEN), as well as for Ethnicity, for which most responses were “No”, and also for Commitment, for which the fewest responses were “No”. Only two questions with lowest inter-rater agreement (or greatest SE) appeared in Table 3, both under “DK” responses.

Values of Randolph’s free-marginal multi-rater *kappa* can range from −1.0 (complete disagreement, below chance) to 1.0 (complete agreement, above chance), with 0.0 indicating agreement equal to chance. Values of *kappa* less than 0.40 are considered poor, values from 0.40 to 0.75 intermediate to good, and values above 0.75 excellent [37]. “Overall agreement”, a slightly more sensitive measure derived from *kappa*, can range from around 33% (equivalent to *kappa* = 0) to 100% (for *kappa* = 1).

Values of SE can range from 0 (no variability or informativity), through 1 (which would result from repeated tosses using an unbiased coin [73]) to > 2.6 for some questionnaires (Body Awareness Questionnaire, data in course of analysis).

For the 60 main survey questions here, SE varies between 0.62 and 1.57, *kappa* between 0 and 0.7.

When SE was plotted against *kappa* and overall agreement for each of the 60 question responses, very strong correlations were found between them (Figure A1).

These correlations are seemingly quite dramatic, but may in fact be trivial. They will be explored in a future paper.
