# Supplementary material for: Individual Differences in Responsiveness to Acupuncture: An Exploratory Survey of Practitioner Opinion
Source: Medicines (Basel). 2018 Aug 6;5(3):85. doi: 10.3390/medicines5030085 (PMC6163768; doi:10.3390/medicines5030085)
Supplement: Supplementary file 1 [file medicines-05-00085-s001.zip › Supplementary material for 7-zip/Supplementary materials I.pdf]

Individual differences in responsiveness to acupuncture: an exploratory survey of practitioner opinion

David F. Mayor [1,*], Lara S. McClure [2] and J. Helgi Clayton McClure [2]

[1] Department of Allied Health Professions and Midwifery, School of Health and Social Work, University of Hertfordshire, Hatfield AL10 9AB, UK

[2] Northern College of Acupuncture, York YO1 6LJ, UK; LaraMcClure@chinese-medicine.co.uk (L.S.M.); helgi.claytonmcclure@oxon.org (J.H.C.M.)

* Correspondence: davidmayor@welwynacupuncture.co.uk; Tel.: +44-1707-320782

Received: date; Accepted: date; Published: date

Supplementary Materials I. Survey questions and results, in summary

(Note that these results do not include those from the four members of the focus group who completed the survey before the official start date.)

Survey on patients' response to acupuncture (revised)

Showing 110 of 110 responses

Showing **all** responses

Showing **all** questions

Response rate: 22%

1 I have read and understood the text above, confirm that the data I provide can be used for the stated purposes of this project, and that I consent to take part in the survey voluntarily.

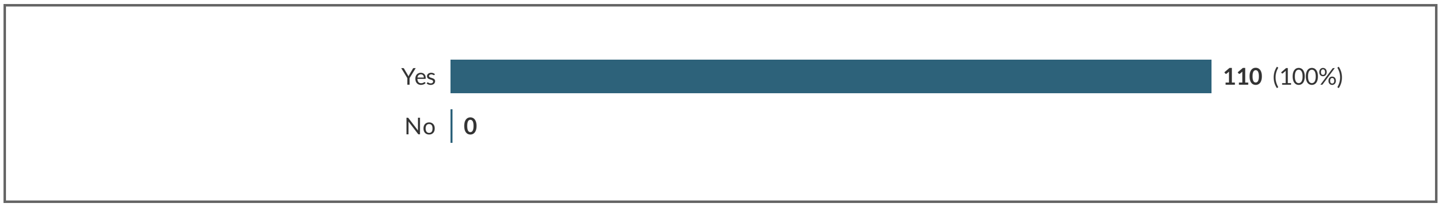

2 Your age (in years)

Showing 5 of 110 responses	
28	298348-298340-26107614
52	298348-298340-26269325
36	298348-298340-26471564
57	298348-298340-26471588
59	298348-298340-26471554

3 Your gender

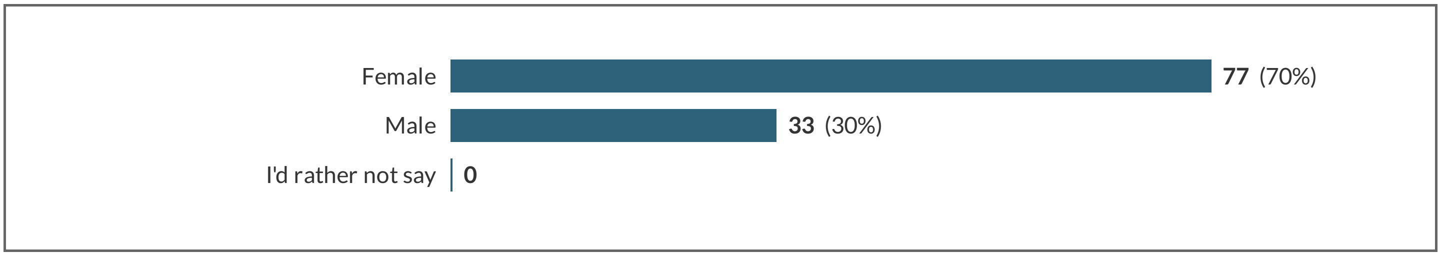

4 What do you consider as your main profession?

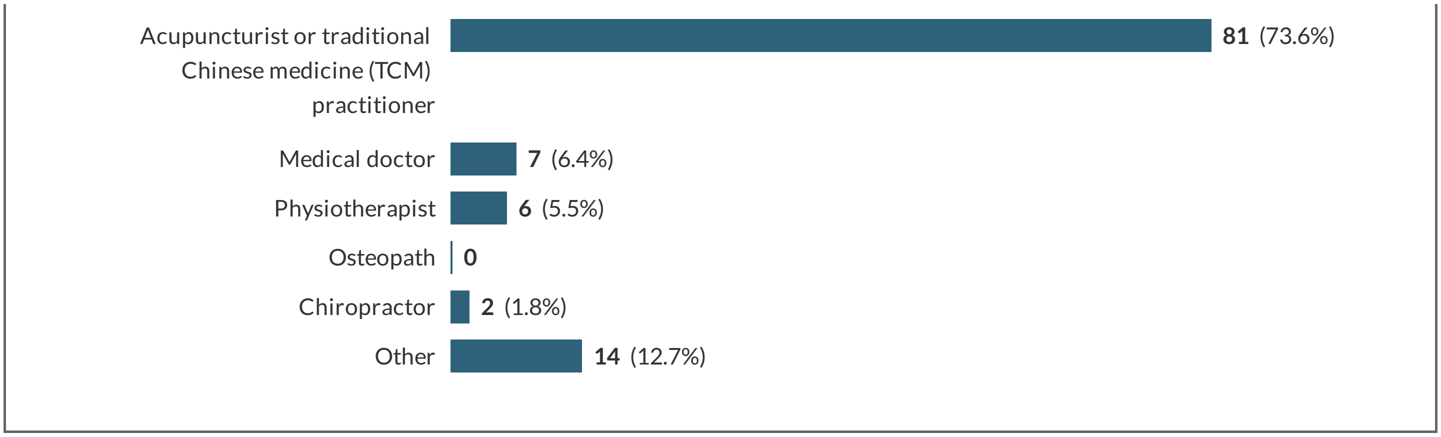

4.a If you selected 'Other', please specify.

**Showing 5 of 16 responses**	
Physical therapist/Acupuncturist	298348-298340-26489299
Dental Hygienist	298348-298340-26681817
Nurse	298348-298340-26774925
Sister in Pain Management	298348-298340-26786544
Advanced Nurse Practitioner/Acupuncturist	298348-298340-27443875

5 With which organisation did you do most of your acupuncture training?

**Showing 5 of 109 responses**	
The Academy of Oriental Medicine	298348-298340-26107614
Acupuncture Academy	298348-298340-26269325
AACP	298348-298340-26471564
BMAS	298348-298340-26471588
CICM	298348-298340-26471554

6 Which of these UK professional acupuncture or Chinese Medicine associations do you belong to? (You may indicate more than one.)

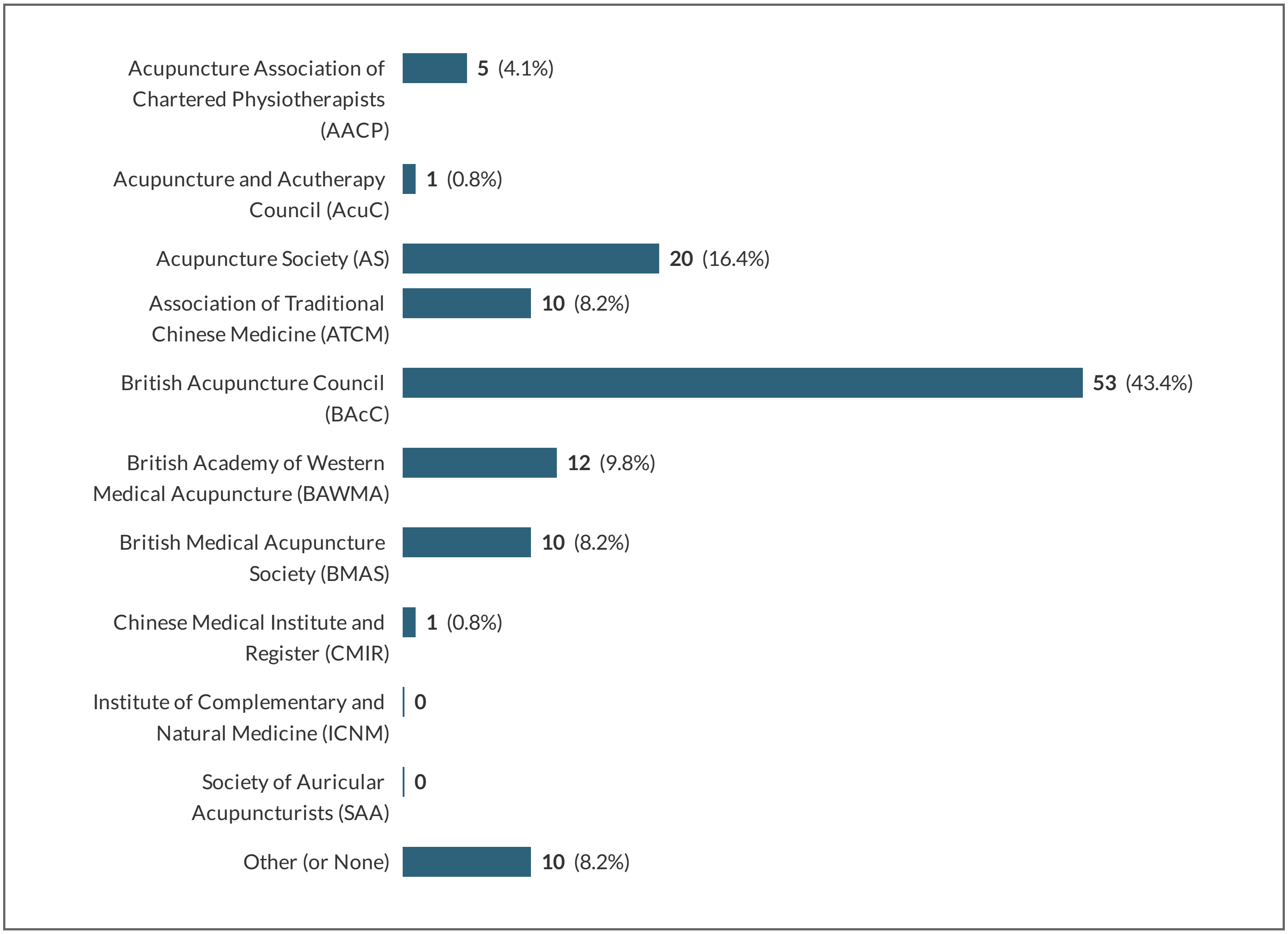

6.a If you selected 'Other (or None)', please specify.

**Showing 5 of 10 responses**	
BRCP - British Register of Complementary Practicioners	298348-298340-26107614
Federation of Holistic Therapists (FHT)	298348-298340-26489299
I have continued to do training with Yuan clinic	298348-298340-26774925
CNHC	298348-298340-26932256
ATCM	298348-298340-29253227

7 What style/s of acupuncture do you use most? (You may indicate more than one.)

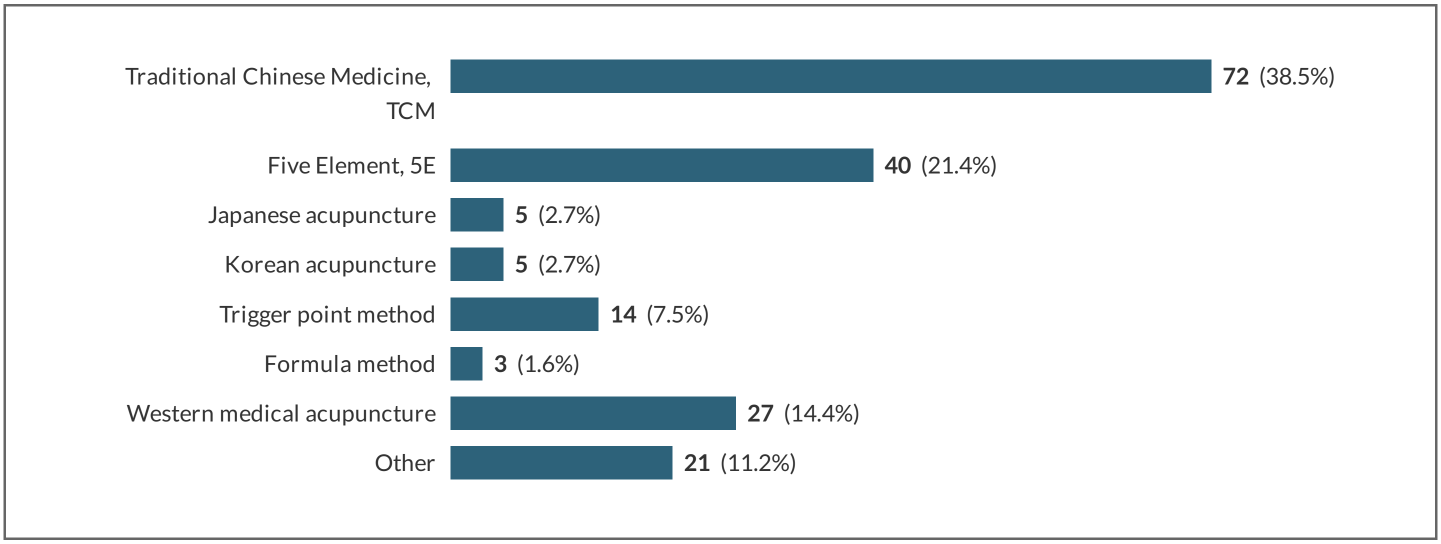

7.a If you selected 'Other', please specify.

Showing 5 of 22 responses	
Si Yuan / Dr Tan Balance Method Acupuncture	298348-298340-26107614
FSN	298348-298340-26471554
Neijing channel	298348-298340-26910831
DNA (Bob Doane)	298348-298340-26924099
Dr Tan Balanced acupuncture	298348-298340-26948161

8 How many years have you used acupuncture in your practice?

Showing 5 of 110 responses	
2	298348-298340-26107614
2	298348-298340-26269325
10	298348-298340-26471564
17	298348-298340-26471588
18	298348-298340-26471554

9 Before you knew about this survey, did you ever consider that patient characteristics (such as their temperament or personality traits) might affect their response to treatment?

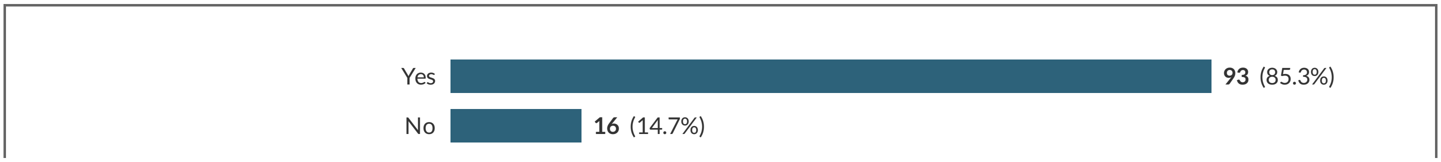

9.a If you answered 'Yes', please give details:

Showing 5 of 89 responses	
Greater results have been observed for patients with expectation of a positive outcome and who enjoy the session	298348-298340-26107614
Having read the john Hamwee book last year, I'd thought it may be connected to the patient CF (make up) but not explored more than that.	298348-298340-26269325
Too broad to give a specific answer here.	298348-298340-26471554
I had considered this carefully. but come to no obvious conclusion.	298348-298340-26472615
Must be willing to have acupuncture. Belief is irrelevant.	298348-298340-26488000

Particular patient characteristics for you to consider

10 Particular patient characteristics, attitudes or experience that you think may contribute to how well (or poorly) they respond to acupuncture. (Information on those that are asterisked can be found under 'More Info', above.)

10.1 Gender

10.1.a Gender - What you THINK NOW affects response

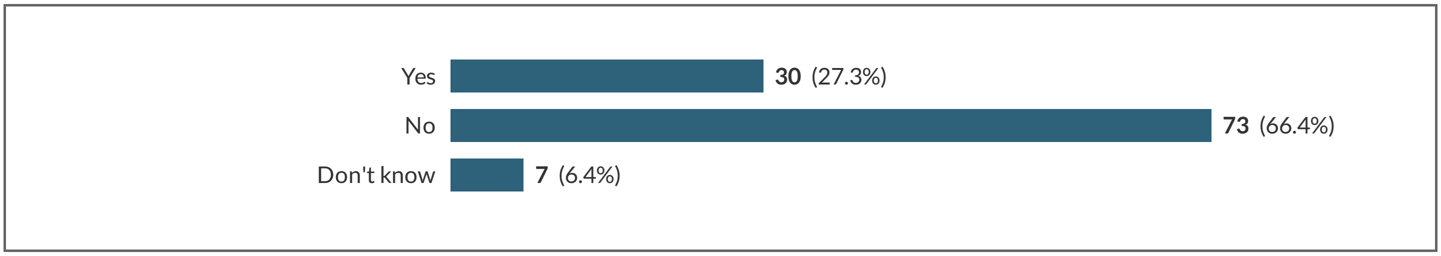

10.1.b Gender - What you THOUGHT THEN (in clinic) affected response

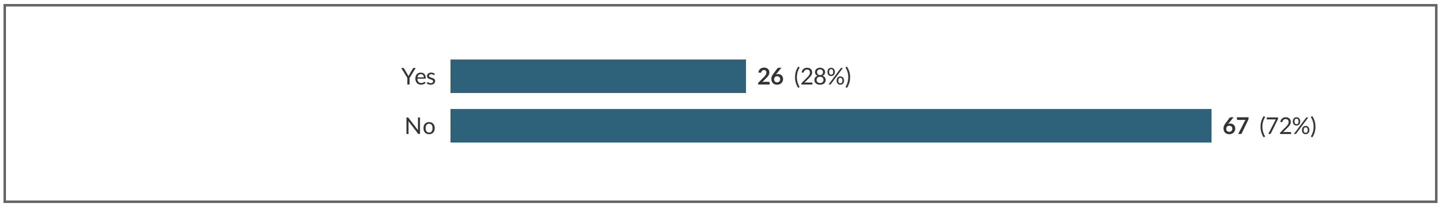

10.1.c Gender - Space for your comments

Showing 5 of 21 responses	
I have always believed male and female have the same chance of successful acupuncture	298348-298340-26786544
Men more often look for direct results relating to presenting condition. Women see other holistic benefits.	298348-298340-26922494
statistics show that women are more likely to seek treatment anyway, so that will possibly show some bias in response.	298348-298340-27190466
to answer this with yes and no seems illconceived to me. I do find women respond better, but only if they are more openminded and less 'locked in' emotionally, which not all women are	298348-298340-26999243
It's not that gender affects the treatment, more that I see less men in clinic so it biases the sample in terms of how well men versus women respond to acupuncture	298348-298340-28415546

10.2 Age

10.2.a Age - What you THINK NOW affects response

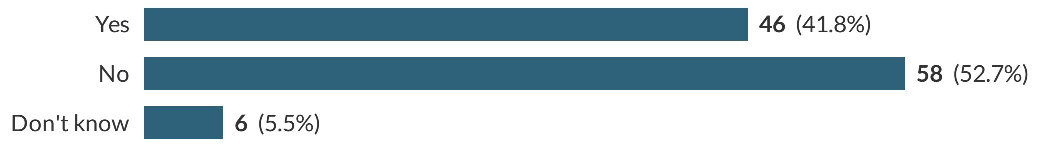

10.2.b Age - What you THOUGHT THEN (in clinic) affected response

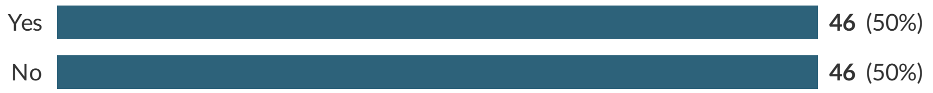

10.2.c Age - Space for your comments

Showing 5 of 22 responses	
Younger patients tend to have less complex conditions to treat so tend to respond more quickly to treatment	298348-298340-26546219
Someone who is very elderly and in a very poor state of health may not respond so well.	298348-298340-26670536
Older people, over 70's, seem less open to change and if on much medication may slow down response time to treatment.	298348-298340-26922494
I find the older generation harder to commit to treatment especially if their doctor is also involved. They tend to want to stop and see what happens with the Western approach.	298348-298340-26997929
multiple comorbidities and multiple medications are probably a factor in response to treatment in elderly.	298348-298340-27190466

10.3 Ethnicity

10.3.a Ethnicity - What you THINK NOW affects response

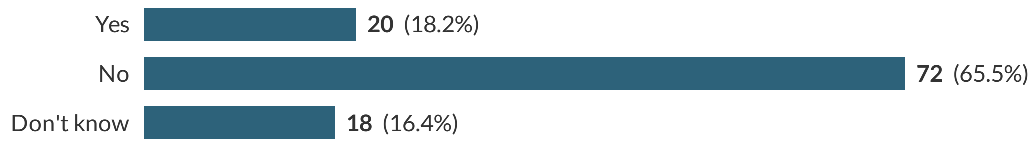

10.3.b Ethnicity - What you THOUGHT THEN (in clinic) affected response

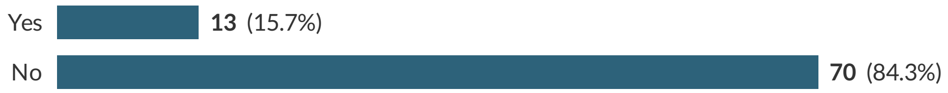

10.3.c Ethnicity - Space for your comments

Showing 5 of 11 responses	
Caucasians seem keener	298348-298340-26971511
Generally Asian over 70 females being cared for by family. Looking for sympathy and attention. If they get better they loose some connection with their carer	298348-298340-26997929
because if my work in the NHS I have treated a variety of nationalities and have not noticed a strong ethnic bias in response.	298348-298340-27190466
I now have a greater understanding of how the concept of privilege impacts on BMAE population.	298348-298340-28048449
Some cultures seem more open to treatment and seek acupuncture for assistance.	298348-298340-29037670

10.4 Birth and prenatal experience

10.4.a Birth and prenatal experience - What you THINK NOW affects response

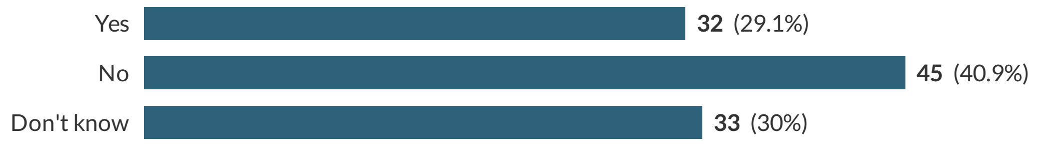

10.4.b Birth and prenatal experience - What you THOUGHT THEN (in clinic) affected response

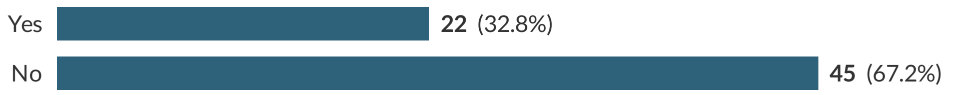

10.4.c Birth and prenatal experience - Space for your comments

Showing 5 of 13 responses	
Except potentially for pre-natal Jing deficiency, acupuncture might not be the best method as could be draining, it would also take a lot of sessions to see significant results.	298348-298340-26107614
Situations such as where the parents are old and the patients Jing is compromised may not respond as well.	298348-298340-26670536
Many had in labour	298348-298340-26971511
many difficult-to-resolve blockages are probably affected by birth and prenatal experiences	298348-298340-26999243
In theory at first re: from jing upwards and condition of parents but more so now after seeing children of older parents and condition of lung and kidney qi on children of some difficult IVF or supported fertility programs	298348-298340-29225890

10.5 Character as a baby, child or adolescent

10.5.a Character as a baby, child or adolescent - What you THINK NOW affects response

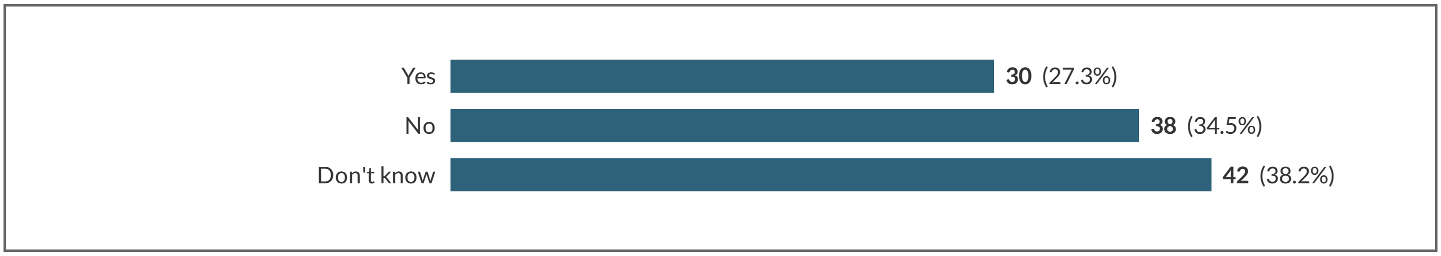

10.5.b Character as a baby, child or adolescent - What you THOUGHT THEN (in clinic) affected response

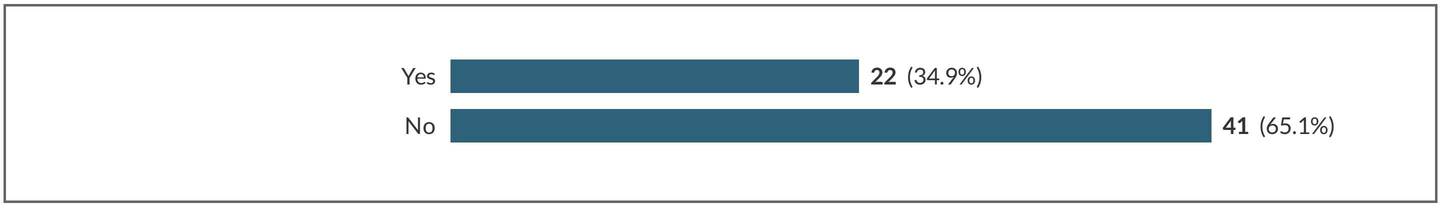

10.5.c Character as a baby, child or adolescent - Space for your comments

Showing 5 of 8 responses	
see above	298348-298340-26999243
may enhance response or make hypersensitive	298348-298340-27904971
Some people seem more open to complementary therapies.	298348-298340-29037670
I think once I have explained to the patient how acupuncture works they are likely to try it. I often ask for feed back using mymop forms. Feedback is more people should know about how acupuncture works!	298348-298340-29668534
N/A as I don't treat babies, children or adolescents in my clinic with Acupuncture	298348-298340-29709659

10.6 Childhood health

10.6.a Childhood health - What you THINK NOW affects response

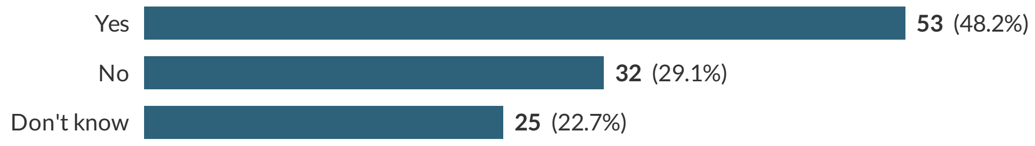

10.6.b Childhood health - What you THOUGHT THEN (in clinic) affected response

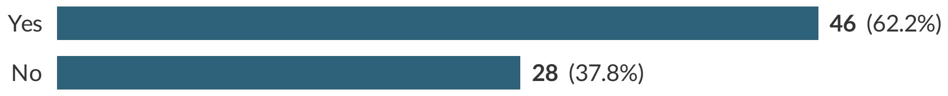

10.6.c Childhood health - Space for your comments

Showing 5 of 12 responses	
Can impact the constitution and health but should not impact the response to the treatment.	298348-298340-26107614
If people have had chronic health problems for a long time their response to treatment is generally not as good	298348-298340-26546219
Patients in poor health as a child and coming to clinic in poor health can need much more long term treatment	298348-298340-26670536
Depending on values, illnesses foe example childhood injury can still bear affect of acupuncture	298348-298340-26786544
see above	298348-298340-26999243

10.7 Family's health in patient's childhood

10.7.a Family's health in patient's childhood - What you THINK NOW affects response

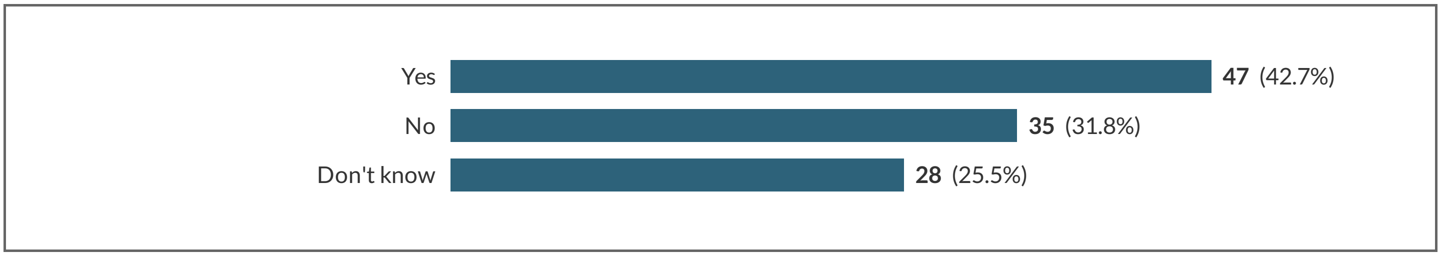

10.7.b Family's health in patient's childhood - What you THOUGHT THEN (in clinic) affected response

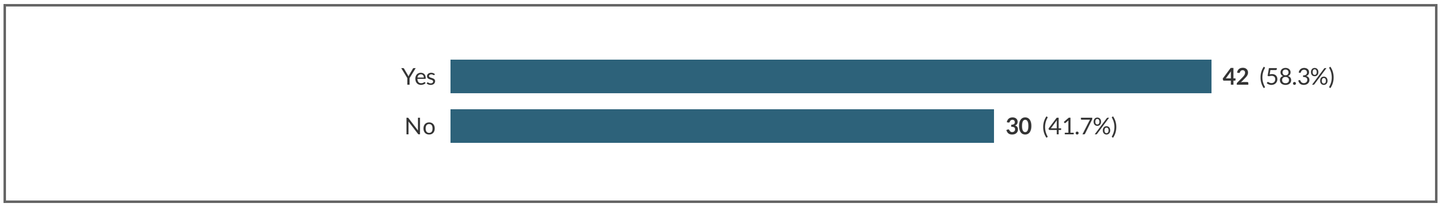

10.7.c Family's health in patient's childhood - Space for your comments

Showing 5 of 12 responses	
Can impact the constitution and health but should not impact the response to the treatment.	298348-298340-26107614
From working in a paediatric acupuncture clinic I can see how the families' health can impact on a childs	298348-298340-26546219
As above	298348-298340-26786544
not sure I would factor that in to my treatment but often it is the effect this can have on their mental health can be more significant tha on their physical health.	298348-298340-27190466
another fuzzy question. If you mean genetic weaknesses, then certainly. If you mean family dynamics, then that affects the treatment outcome also, but for very different reasons	298348-298340-26999243

10.8 Childhood poverty

10.8.a Childhood poverty - What you THINK NOW affects response

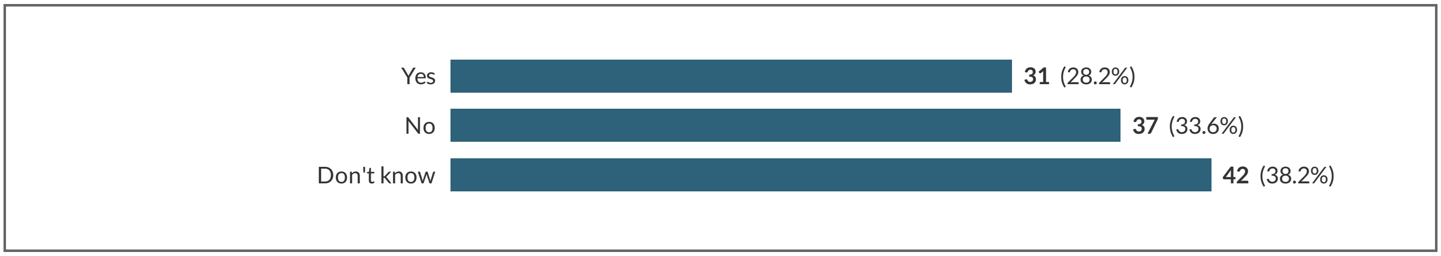

10.8.b Childhood poverty - What you THOUGHT THEN (in clinic) affected response

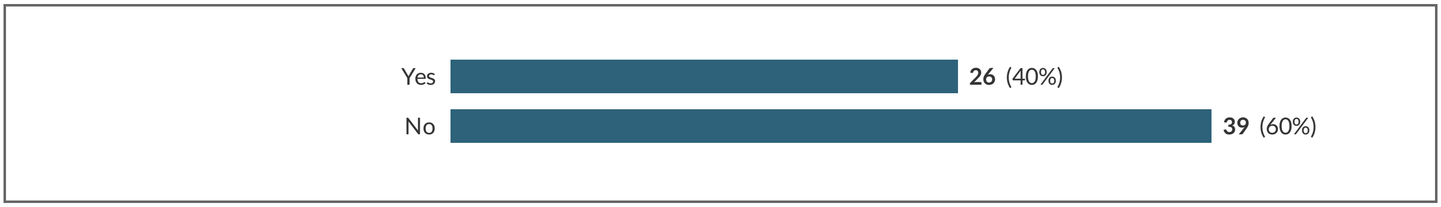

10.8.c Childhood poverty - Space for your comments

Showing 5 of 6 responses	
Can impact the constitution and health but should not impact the response to the treatment.	298348-298340-26107614
pretty obvious as disease factors, but do they affect the response to acupuncture. I repeat: a patient with a healthy, balanced qi will respond better to acupuncture treatment and vice versa .	298348-298340-26999243
Acupuncture often seen as a luxury treatment.	298348-298340-29720447
I haven't dealt with anyone in this category	298348-298340-29772908
I have not made any connection.	298348-298340-30079001

10.9 Childhood abuse, neglect, trauma or bereavement

10.9.a Childhood abuse, neglect, trauma or bereavement - What you THINK NOW affects response

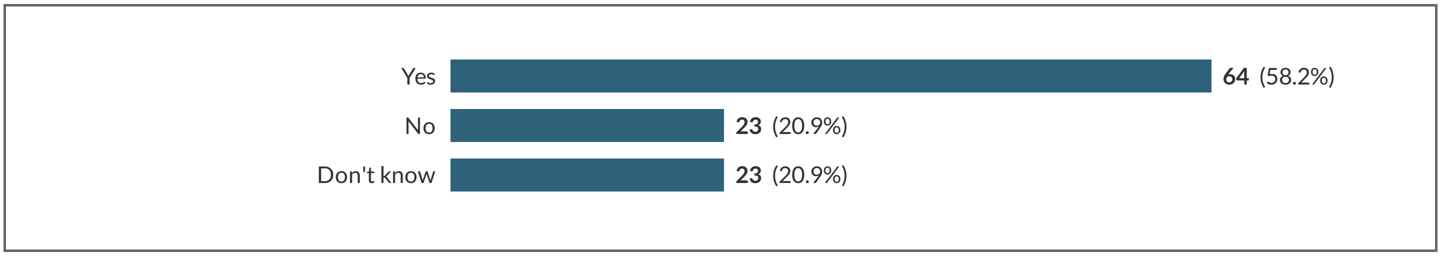

10.9.b Childhood abuse, neglect, trauma or bereavement - What you THOUGHT THEN (in clinic) affected response

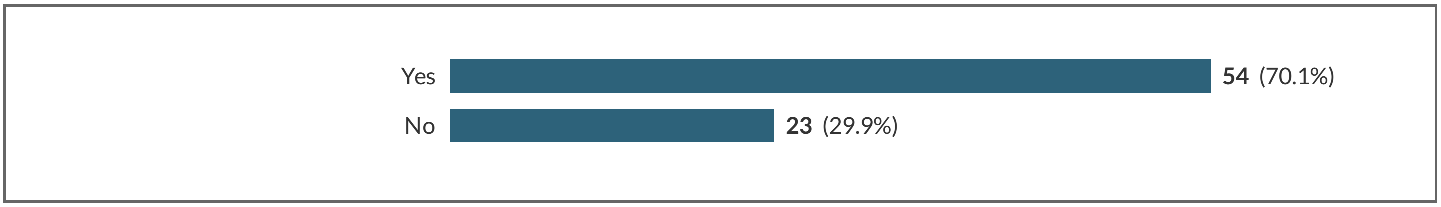

10.9.c Childhood abuse, neglect, trauma or bereavement - Space for your comments

Showing 5 of 14 responses	
Can impact the constitution and health but should not impact the response to the treatment.	298348-298340-26107614
A high proportion of patients I see have had a form of abuse as a child and since I see these in high numbers I felt it was linked to their poor health later on	298348-298340-26546219
Tra	298348-298340-26670536
At times this can be a challenge and can take longer to attain acupuncture benefit	298348-298340-26786544
There's a difference between a predictor for bad response and a reason for a bad response	298348-298340-26910831

10.10 Later abuse or trauma

10.10.a Later abuse or trauma - What you THINK NOW affects response

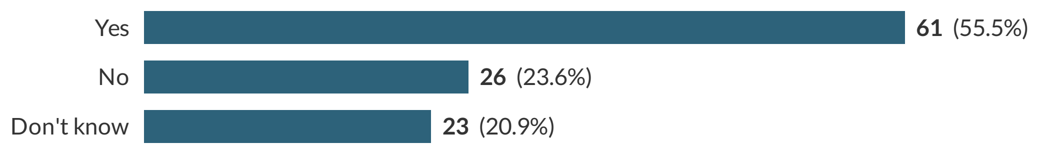

10.10.b Later abuse or trauma - What you THOUGHT THEN (in clinic) affected response

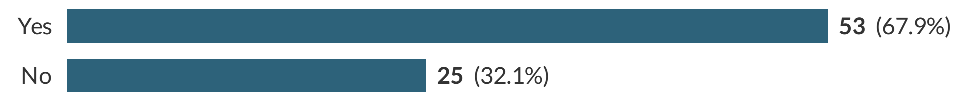

10.10.c Later abuse or trauma - Space for your comments

Showing 5 of 10 responses	
Can impact the constitution and health but should not impact the response to the treatment.	298348-298340-26107614
As above	298348-298340-26546219
Some situations partners control over the patient can affect outcome.	298348-298340-26670536
all types of abuse and trauma, including PTSD have a huge effect on the patients ability to cope with pain/illness, as shown by my work in a Chronic Pain unit..	298348-298340-27190466
pretty obvious, plus see above	298348-298340-26999243

10.11 Level of education

10.11.a Level of education - What you THINK NOW affects response

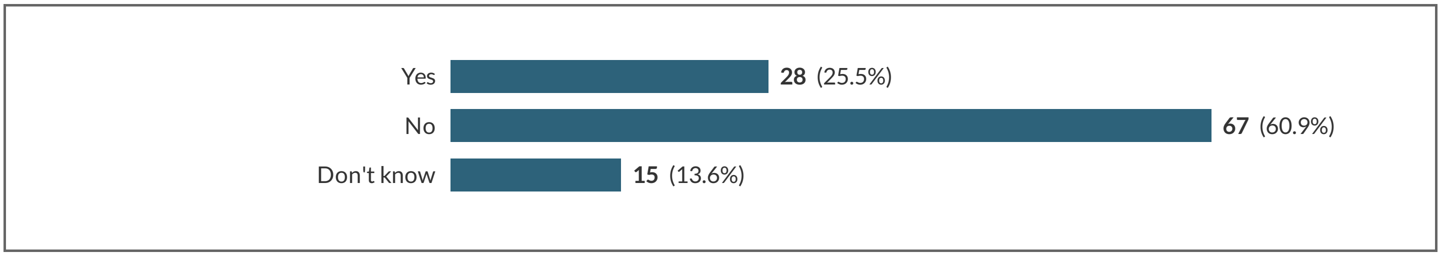

10.11.b Level of education - What you THOUGHT THEN (in clinic) affected response

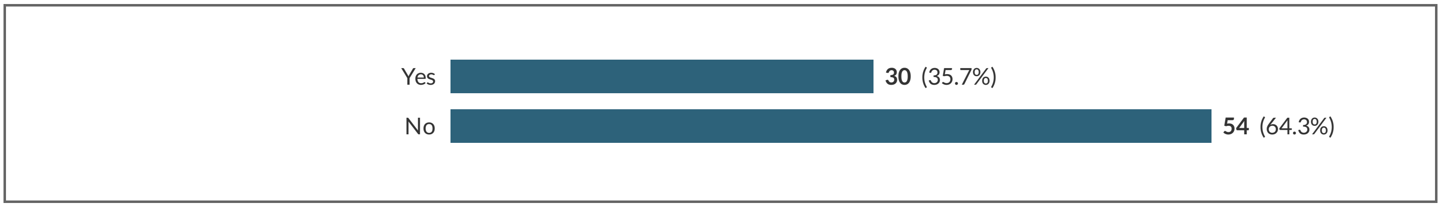

10.11.c Level of education - Space for your comments

**Showing 5 of 12 responses**	
The acupuncturist should be able to explain the treatment to all levels of education. Negative perception of the treatment or confusion could lead to poor results.	298348-298340-26107614
Highly educated people are skeptical of traditional medicines and seem to be less appreciate of caring and kindness	298348-298340-26471554
I believe education level can affect the ability to understand ourselves	298348-298340-26924099
again research shows that patients of low educational level are more likely to suffer chronic pain and other illnesses. some respond well but some are unable to understand the deeper influences on their physical + mental health and continue to seek an answer in medications or other treatment like that, even when all treatments they have had up to now have failed..	298348-298340-27190466
Stephen Hawkins is well educated, does that mean he responds to acupuncture better?	298348-298340-26999243

10.12 Relationship/marital status

10.12.a Relationship/marital status - What you THINK NOW affects response

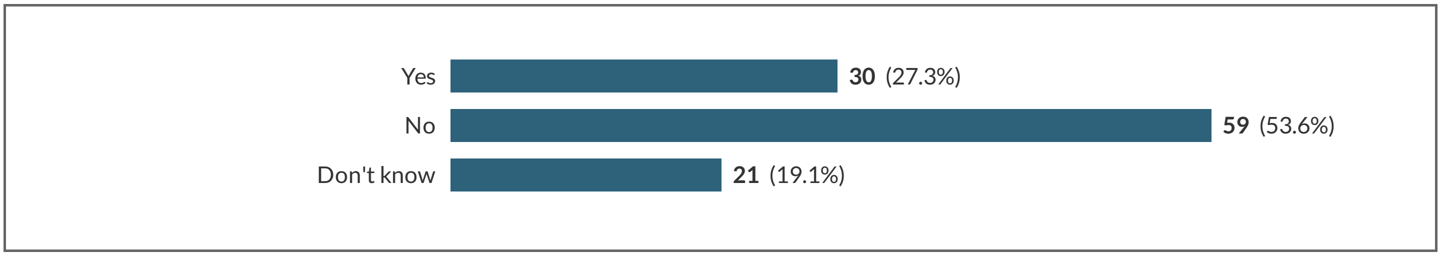

10.12.b Relationship/marital status - What you THOUGHT THEN (in clinic) affected response

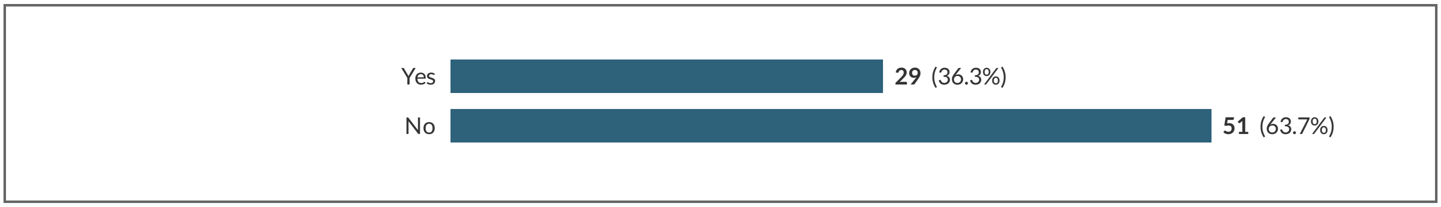

10.12.c Relationship/marital status - Space for your comments

**Showing 5 of 6 responses**	
Can impact the emotions and health but should not impact the response to the treatment.	298348-298340-26107614
It's not the actual status but how they are coping with any changes	298348-298340-27190466
if you are in a bad relationship, this affects your qi and how you respond to acupuncture, marital status is a quite different issue	298348-298340-26999243
Happiness and stress level	298348-298340-29691597
Generally, people in happy relationships respond better to treatments.	298348-298340-30079001

10.13 Social network and/or support

10.13.a Social network and/or support - What you THINK NOW affects response

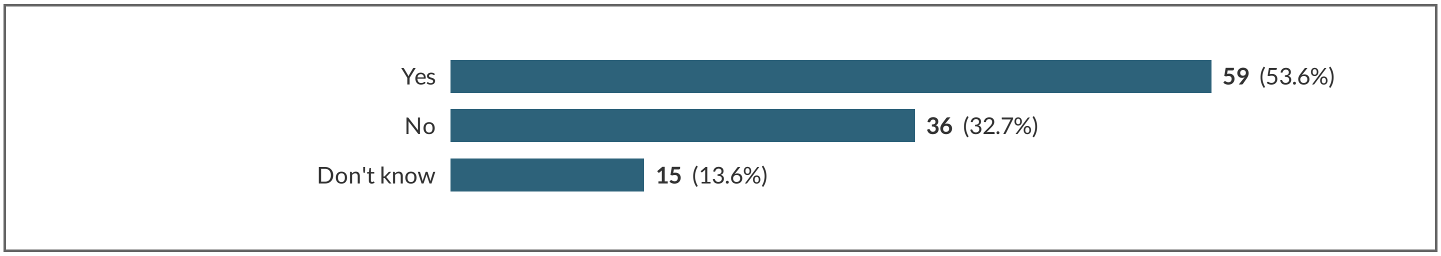

10.13.b Social network and/or support - What you THOUGHT THEN (in clinic) affected response

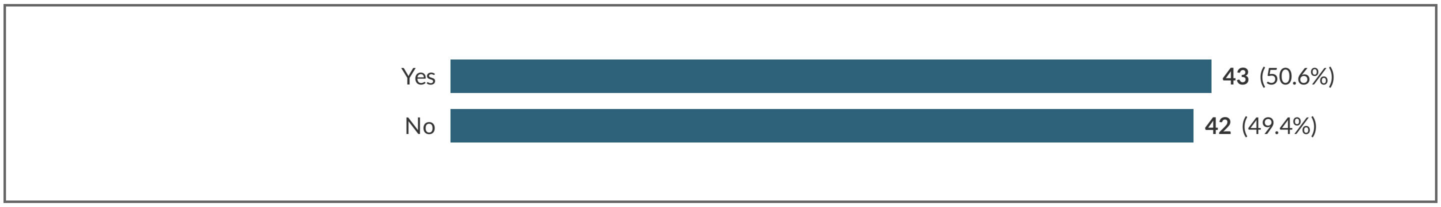

10.13.c Social network and/or support - Space for your comments

**Showing 5 of 10 responses**	
From experience patients who have poor social circles cope less well in general life which can then impact on their health	298348-298340-26546219
Example of problems of smoking, drug, gambling and alcohol addiction.	298348-298340-26670536
People with a strong support around them tend to fare better	298348-298340-26924099
research supports that this is a factor in people's ability to cope with any health problems and helps their mental health and experience with patients backs this up. chronic pain patients often have poor support systems from family and generally.	298348-298340-27190466
enhances likely good response	298348-298340-27904971

10.14 Housing situation

10.14.a Housing situation - What you THINK NOW affects response

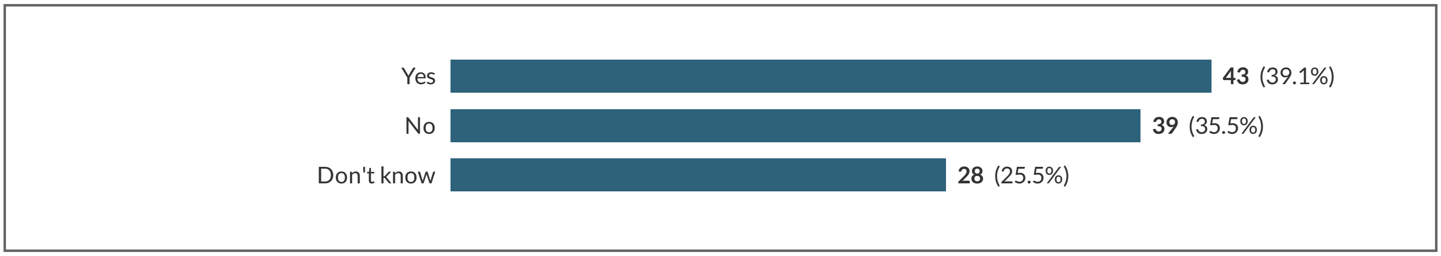

10.14.b Housing situation - What you THOUGHT THEN (in clinic) affected response

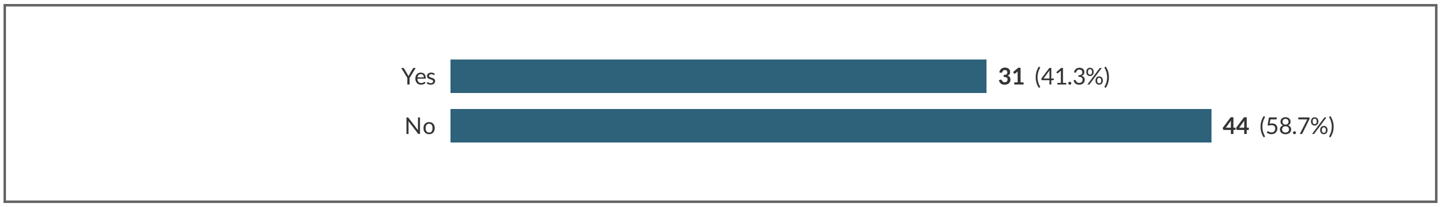

10.14.c Housing situation - Space for your comments

Showing 5 of 8 responses	
Can impact the constitution and health but should not impact the response to the treatment.	298348-298340-26107614
It is not unusual to find th patients main problem is poor conditions. e.g. Serious Damp issues in the home.	298348-298340-26670536
this can be a huge stress, either the quality of the accommodation, the neighbours, or physically unable to cope with issues like stairs.	298348-298340-27190466
but only if they affect the state of the persons qi	298348-298340-26999243
People with a difficult or uncertain housing situation seem to find it hard to find stability which has an impact on all areas of their life and health. I notice this particularly with younger people and students.	298348-298340-29161230

10.15 Work situation

10.15.a Work situation - What you THINK NOW affects response

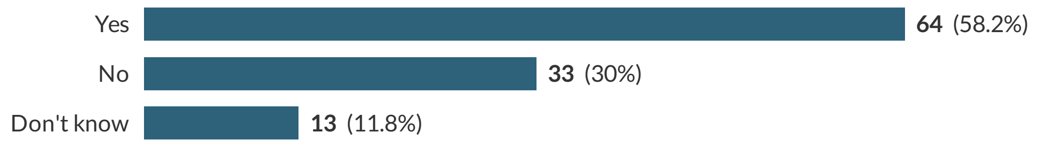

10.15.b Work situation - What you THOUGHT THEN (in clinic) affected response

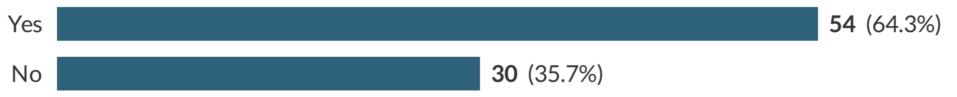

10.15.c Work situation - Space for your comments

Showing 5 of 13 responses	
Can impact the constitution and health but should not impact the response to the treatment.	298348-298340-26107614
It affects their resources - both financially and time available for treatment/life changes.	298348-298340-26489299
I have treated a number of patients whose stressful jobs seem to have contributed to their ill health	298348-298340-26546219
Inability to improve conditions of overwork and stress if it is cause of the main complaint.	298348-298340-26670536
Uncertainty over work income affects stress levels and treatment outcomes	298348-298340-26924099

10.16 Financial situation

10.16.a Financial situation - What you THINK NOW affects response

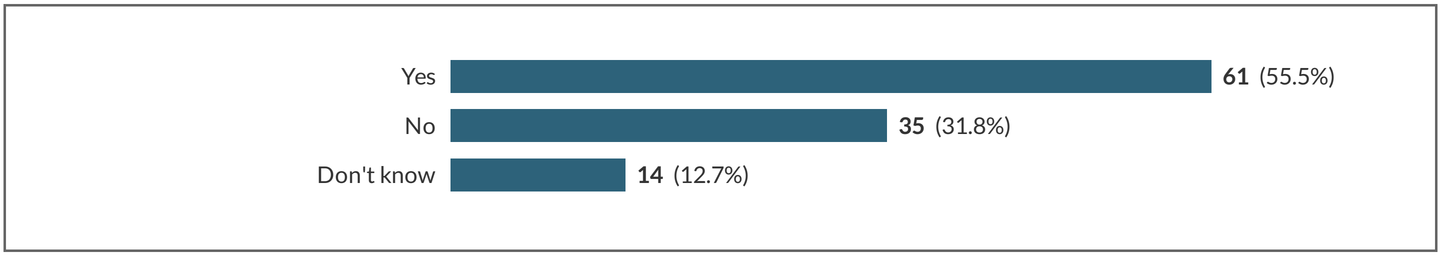

10.16.b Financial situation - What you THOUGHT THEN (in clinic) affected response

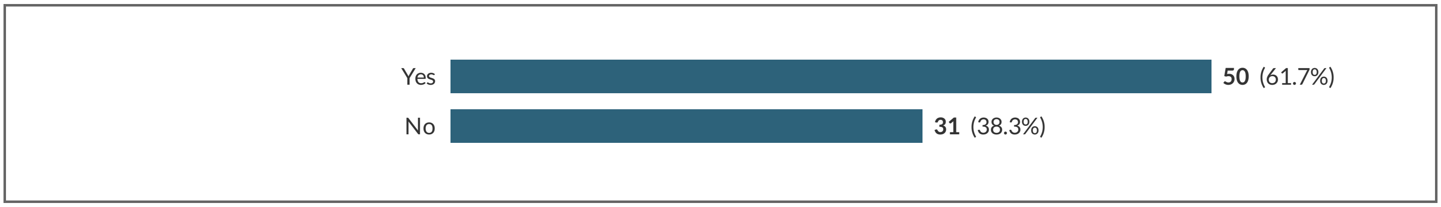

10.16.c Financial situation - Space for your comments

Showing 5 of 12 responses	
Can impact the constitution and health but should not impact the response to the treatment.	298348-298340-26107614
Of course.	298348-298340-26489299
Can’t afford treatment. Different issue?	298348-298340-26910831
Affects ability to continue treatment after initial relief (usually stop too soon)	298348-298340-26924099
people are often working more than 1 job or long hours to earn enough to support their family, so again physical and mental stress. also benefits and litigation often hidden factors that affect people's progress - either the stress of going through the process or secondary gains.	298348-298340-27190466

10.17 Religious beliefs or practices

10.17.a Religious beliefs or practices - What you THINK NOW affects response

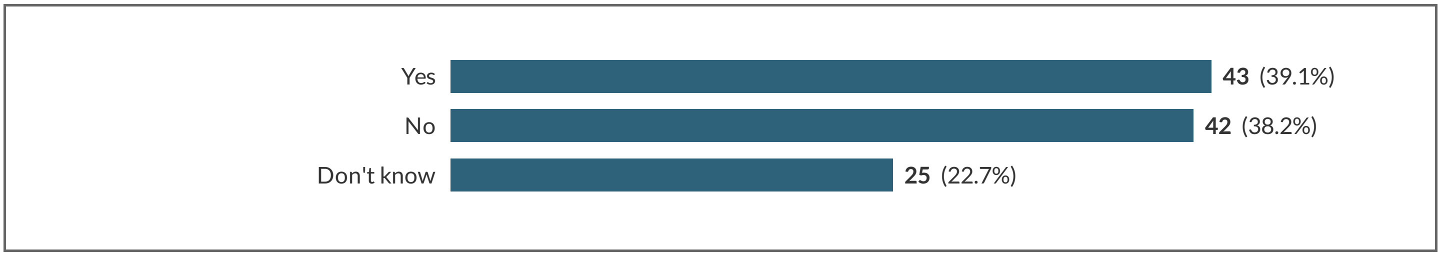

10.17.b Religious beliefs or practices - What you THOUGHT THEN (in clinic) affected response

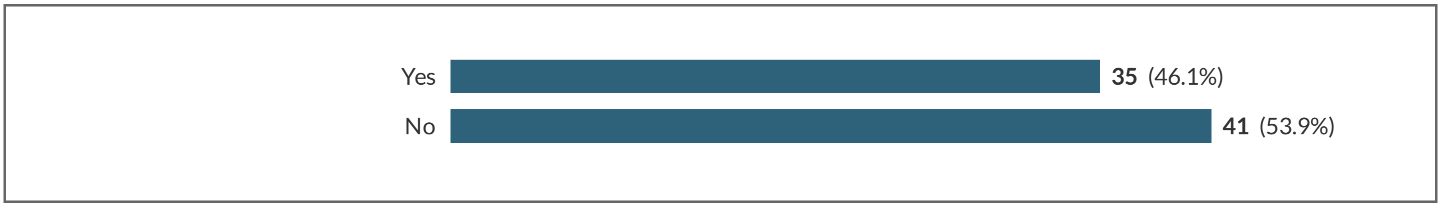

10.17.c Religious beliefs or practices - Space for your comments

Showing 5 of 10 responses	
Unless leading to negative perception of the treatment which could lead to poor results.	298348-298340-26107614
I think this is more likely to affect patient's decision to attend in the first place.	298348-298340-26489299
Faith affects everything	298348-298340-26910831
have had people who say that they area concerned about the negative influence acupuncture could potentially have, usually Christians rather than other belief systems.	298348-298340-27190466
may reduce response	298348-298340-27904971

10.18 Attitude to nature &/or technology

10.18.a Attitude to nature &/or technology - What you THINK NOW affects response

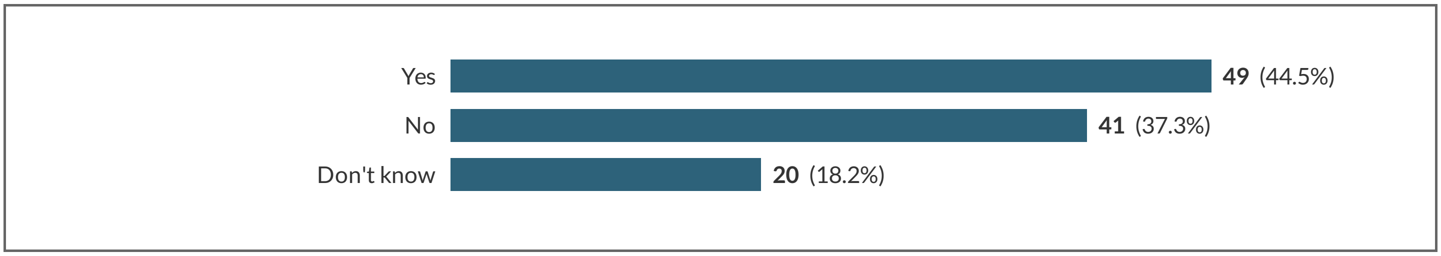

10.18.b Attitude to nature &/or technology - What you THOUGHT THEN (in clinic) affected response

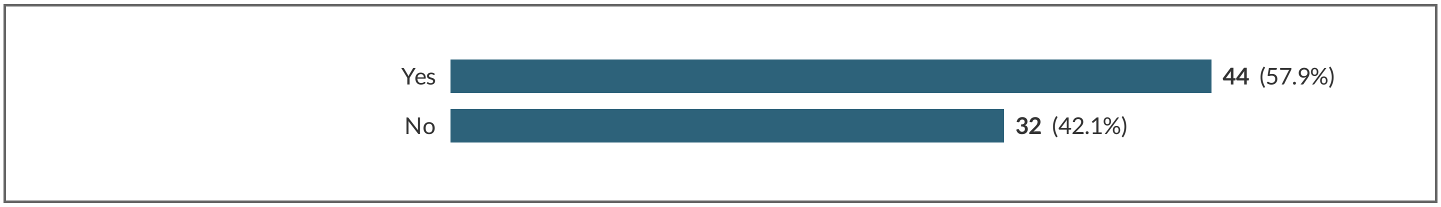

10.18.c Attitude to nature &/or technology - Space for your comments

Showing 5 of 10 responses	
Working with nature is essential	298348-298340-26910831
how well (or poorly) they respond to acupuncture is obviously a factor in the context of them understanding nature as a dynamic state of qi. What that has to do with their attitude to technology, I am not sure.	298348-298340-26999243
may enhance response	298348-298340-27904971
A patient's attitude to nature (where it reflects Chinese medicine philosophy) seems to positively affect treatment outcome	298348-298340-28721109
People who are more linked to nature are more open to acupuncture. Also preferring Natural Medicine and being willing to consider lifestyle changes.	298348-298340-29037670

10.19 General state of health

10.19.a General state of health - What you THINK NOW affects response

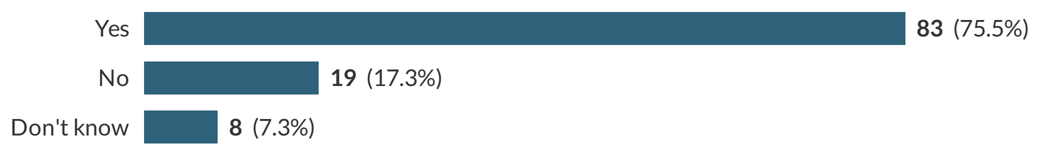

10.19.b General state of health - What you THOUGHT THEN (in clinic) affected response

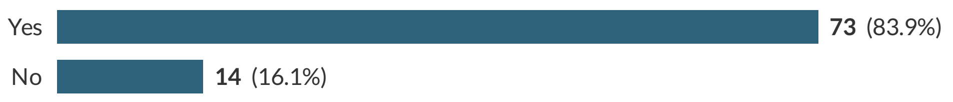

10.19.c General state of health - Space for your comments

Showing 5 of 13 responses	
Unless patient has severely damaged their Jing, acupuncture might not be the best method as could be draining, it would also take a lot of sessions to see significant results.	298348-298340-26107614
Difficult to separate this from presenting symptom(s)	298348-298340-26488000
It is likely to take several sessions to fully understand the state of health of the patient - so in clinic you deal with what you know initially.	298348-298340-26489299
Similar to first question's answer if very elderly and in extremely poor state of health.	298348-298340-26670536
if people aren't making the choices in their lifestyle that supports good health then it is much harder for any treatment to have a sustained effect	298348-298340-27190466

10.20 Neurochemical or hormonal levels* (e.g. ease of neurotransmitter ‘expression’)

10.20.a Neurochemical or hormonal levels* (e.g. ease of neurotransmitter ‘expression’) - What you THINK NOW affects response

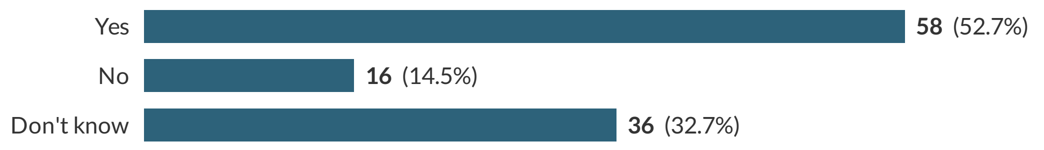

10.20.b Neurochemical or hormonal levels* (e.g. ease of neurotransmitter ‘expression’) - What you THOUGHT THEN (in clinic) affected response

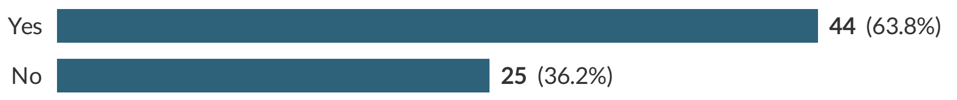

10.20.c Neurochemical or hormonal levels* (e.g. ease of neurotransmitter ‘expression’) - Space for your comments

Showing 5 of 12 responses	
Not in my experience as have seen a case of patient with punctured spinal cord responding well to acupuncture and regaining sensitivity. It is a long process however.	298348-298340-26107614
Don't understand this	298348-298340-26488000
I'm doing an MSc in pain management so have learnt a lot about this recently	298348-298340-26546219
obviously	298348-298340-26999243
Some patients have stronger reactions, it could be linked to this.	298348-298340-29037670

10.21 Central sensitisation*

10.21.a Central sensitisation* - What you THINK NOW affects response

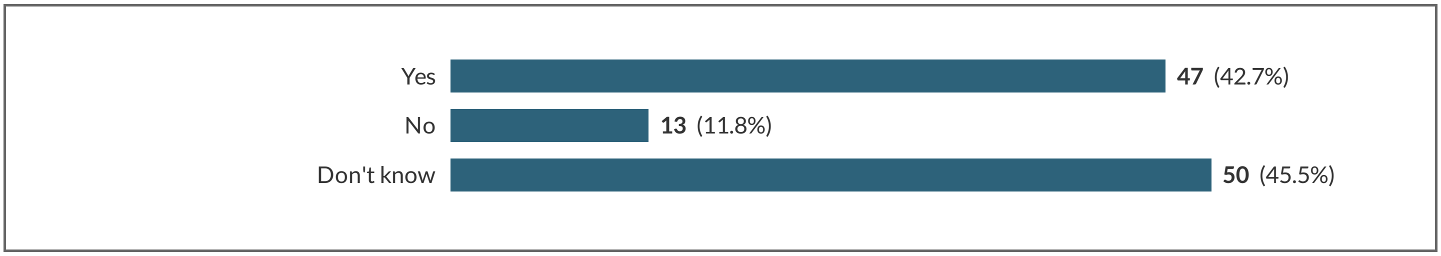

10.21.b Central sensitisation* - What you THOUGHT THEN (in clinic) affected response

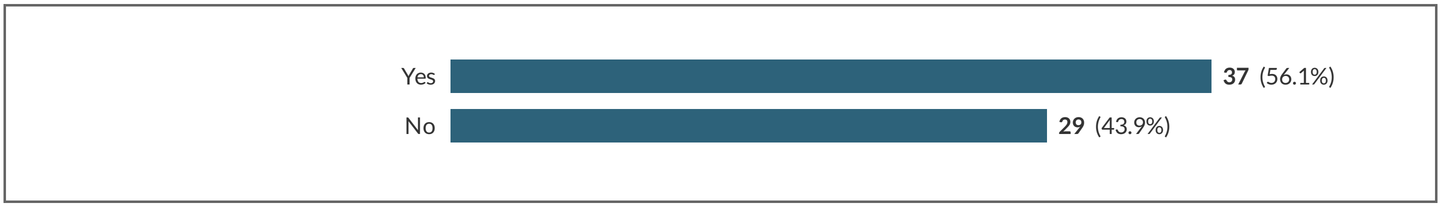

10.21.c Central sensitisation* - Space for your comments

Showing 5 of 11 responses	
Fibromyalgia patients respond well to acupuncture, especially SiYuan BMA.	298348-298340-26107614
As above	298348-298340-26546219
what is this? If this is sensitisation then no	298348-298340-26670536
If I understand, this affects the ability to receive needles	298348-298340-26910831
Don't know	298348-298340-26948161

10.a Space for any additional comments you may wish to make:

Showing 5 of 12 responses	
Some questions are difficult to answer as patients may not be honest about their responses as they wish to please - or be difficult.	298348-298340-26489299
I did a lecture on this to the local BAcC group. I explained the effects on the nervous system, Flag system, special questions to ask about mood, sleep, differential diagnosis of MSK problems, identifying trigger points, Hypermobility(a significant percentage of our younger patients are undiagnosed Hypermobile people which often explains a lot of their health problems), Pain Management principles, Acceptance + Commitment therapy + Mindfulness, as well as acupuncture approaches that may be useful.	298348-298340-27190466
Feel that there should have been a box for possibly.	298348-298340-27443875
neurotransmitter 'expression' and central sensitisation are important factors regarding the response to acupuncture, but please don't reduce it to just that	298348-298340-26999243
I do an audit of all my acupuncture patients annually, covering basic patient characterisitcs. hence age gender eethnicty repos based on this	298348-298340-27654180

11 Particular patient characteristics, attitudes or experience that you think may contribute to how well (or poorly) they respond to acupuncture. (Information on those that are asterisked can be found under 'More Info', above.)

11.1 Diet and nutritional status

11.1.a Diet and nutritional status - What you THINK NOW affects response

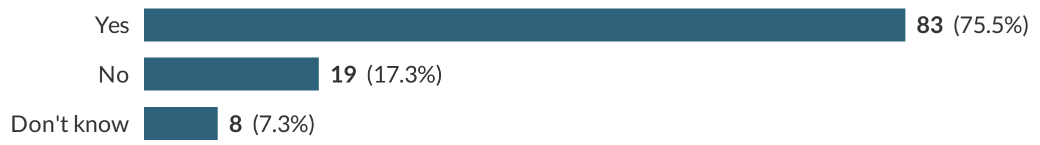

11.1.b Diet and nutritional status - What you THOUGHT THEN (in clinic) affected response

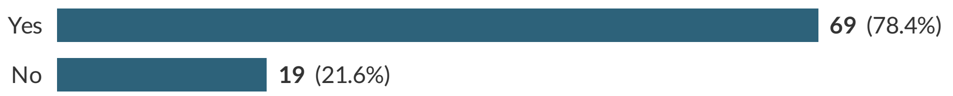

11.1.c Diet and nutritional status - Space for your comments

Showing 5 of 14 responses	
This can have an impact on health or constitution.	298348-298340-26107614
Need blood to receive treatment	298348-298340-26910831
Blood deficiency	298348-298340-26951369
if the person is not giving their body what it needs to build and repair, their nervous system and local tissues will not have the resources needed to improve. also there are often mental/emotional aspects to this issue that may be difficult to change.	298348-298340-27190466
all actions (external, internal, emotional, etc.) that affect the state of our qi, blood, jing, shen, etc. will have an effect on how we function, both in the language of CM or that of biomedical science, which you seem to favour. The more balanced we are, the more favourably we will respond to acupuncture treatment in my opinion.	298348-298340-26999243

11.2 Exercise taken

11.2.a Exercise taken - What you THINK NOW affects response

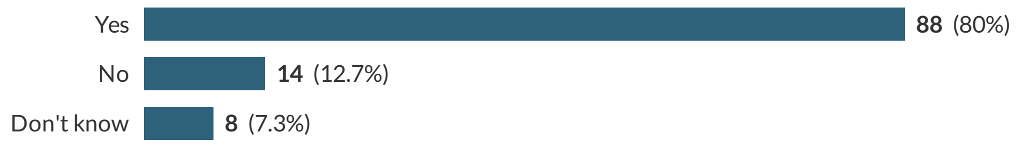

11.2.b Exercise taken - What you THOUGHT THEN (in clinic) affected response

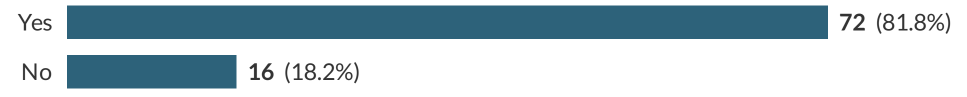

11.2.c Exercise taken - Space for your comments

Showing 5 of 13 responses	
Could marginally obtain better response as active people should have their Qi and Blood flowing more freely.	298348-298340-26107614
Exercise inappropriate to specific MSK condition will prevent improvement	298348-298340-26951369
the issue can be too much, too little or incorrect for their particaulr problem. unfortunately most acupuncturists are not equipped to properly assess this or give a suitable exercise programme. even less experienced physios miss things or take an unsuitable approach that I end up having to correct.	298348-298340-27190466
see under diet and nutrition	298348-298340-26999243
People/any trace of life it's stay alive cause of the movement	298348-298340-27644723

11.3 Ability to relax

11.3.a Ability to relax - What you THINK NOW affects response

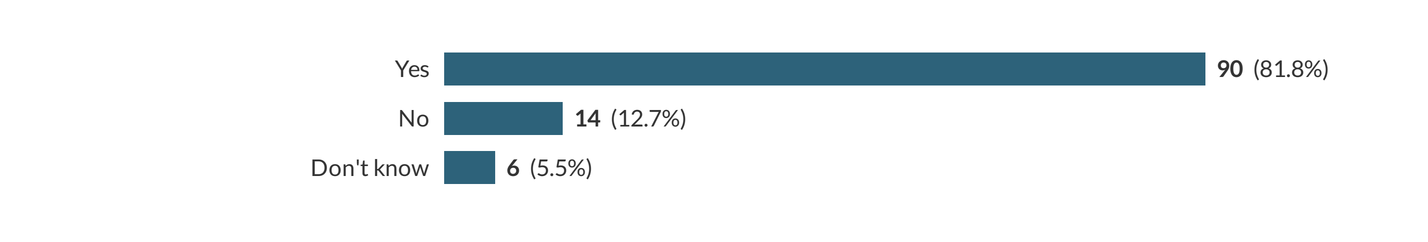

11.3.b Ability to relax - What you THOUGHT THEN (in clinic) affected response

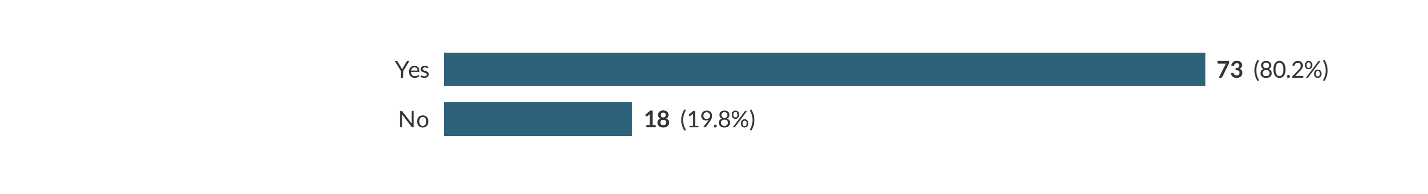

11.3.c Ability to relax - Space for your comments

Showing 5 of 13 responses	
Ability to relax is tied in with the perception of the treatment.	298348-298340-26107614
Alarm state in nervous system is a barrier	298348-298340-26910831
some people do gradually learn to relax as they listen to a tape during their treatment that talks them through a technique aimed at chronic pain patients. however with some mental health issues, letting go means that thoughts they are trying to suppress may come to the fore, which they don't like. as we are not a Mental Health unit, we would discus this before considering some patients for acupuncture.	298348-298340-27190466
see under diet and nutrition	298348-298340-26999243
It's hard to do an accurate SLR most of the time because people don't know to control their muscle/relax	298348-298340-27644723

11.4 Overall satisfaction with life

11.4.a Overall satisfaction with life - What you THINK NOW affects response

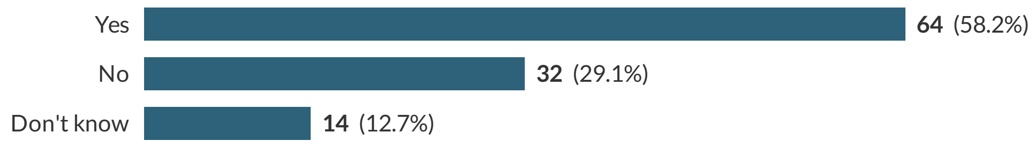

11.4.b Overall satisfaction with life - What you THOUGHT THEN (in clinic) affected response

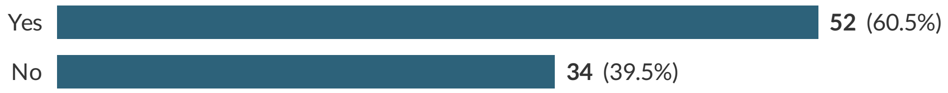

11.4.c Overall satisfaction with life - Space for your comments

Showing all 5 responses	
Dissatisfied (with their life) patient refusing to change their situation for the better will lose benefit of treatment quickly as emotions are an aggravating factor, will finally end up not responding at all.	298348-298340-26107614
see under diet and nutrition	298348-298340-26999243
depends if this is a symptom/ being treated	298348-298340-29496506
general mood of this patient was pessimistic and remained the same after acupuncture treatment.	298348-298340-29668534
Yes, the positive attitude helps.	298348-298340-30079001

11.5 Feeling in control of life

11.5.a Feeling in control of life - What you THINK NOW affects response

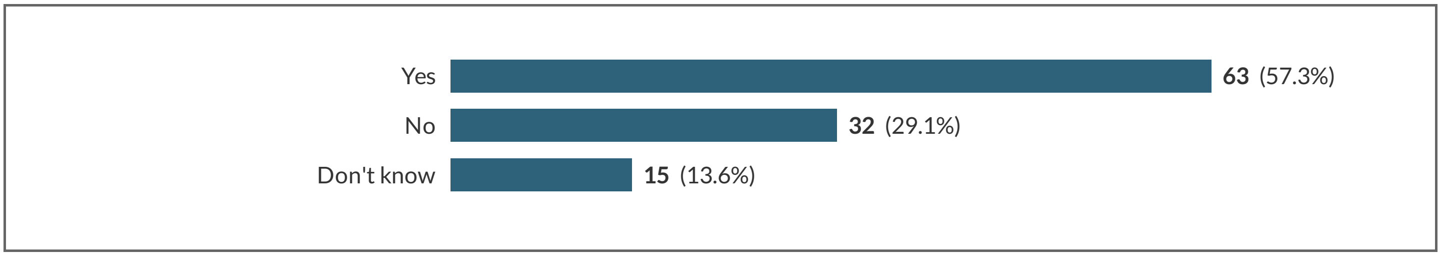

11.5.b Feeling in control of life - What you THOUGHT THEN (in clinic) affected response

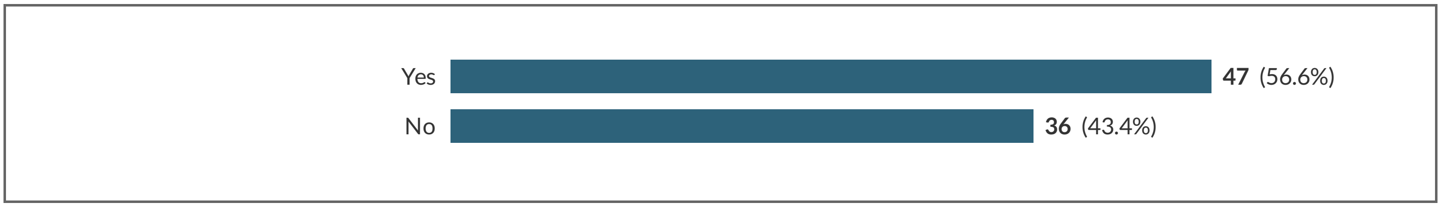

11.5.c Feeling in control of life - Space for your comments

Showing 5 of 8 responses	
Similar to above.	298348-298340-26107614
how many people in the whole population feel in control of all aspects of their life anyway?	298348-298340-27190466
Feeling in control of life, what exactly is that? For this life's destiny I follow the signs that I can detect, that is not the same as being in control of it. Am I in control of myself , the acupuncturist, the husband, the father, etc. Yes, that will affect my health via the 7 emotions and how I react to acupuncture	298348-298340-26999243
depends if this is a symptom/ being treated	298348-298340-29496506
Leads to positive attitude.	298348-298340-29682718

11.6 Self-motivation

11.6.a Self-motivation - What you THINK NOW affects response

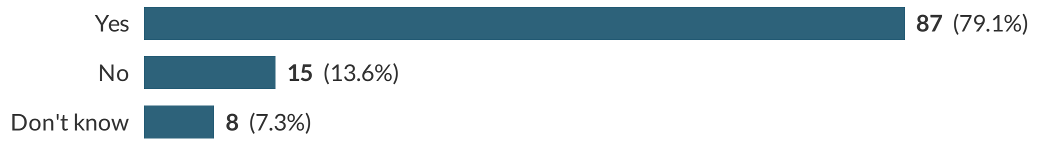

11.6.b Self-motivation - What you THOUGHT THEN (in clinic) affected response

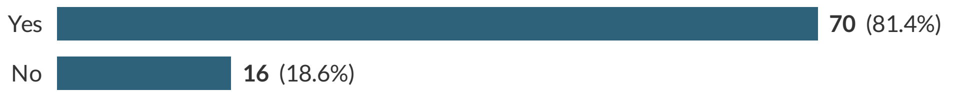

11.6.c Self-motivation - Space for your comments

Showing 5 of 9 responses	
Similar to above.	298348-298340-26107614
Motivational interviewing can be very helpful in bringing out a persons readiness for change. as most people with chronic pain need to change something, this can be useful in sussing out who may be at the right stage to make the necessary changes. for some acupuncture may help get them to this stage, but for others mental/emotional/social factors may be a block. we would still try and work with some patient to see if we can overcome these blocks.	298348-298340-27190466
see under diet and nutrition	298348-298340-26999243
Motivation to attend sessions.	298348-298340-29037670
depends if this is a symptom/ being treated	298348-298340-29496506

11.7 Sense of self-esteem

11.7.a Sense of self-esteem - What you THINK NOW affects response

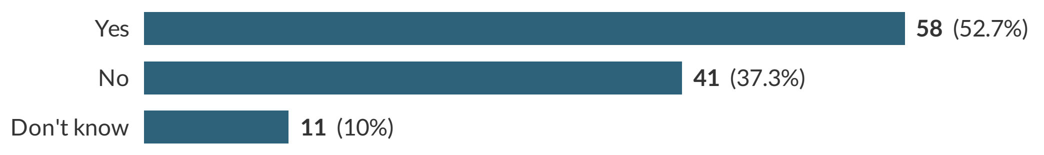

11.7.b Sense of self-esteem - What you THOUGHT THEN (in clinic) affected response

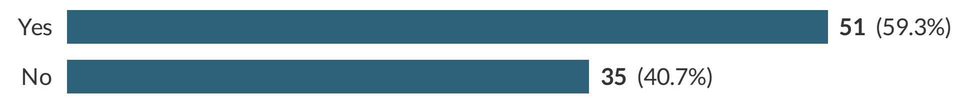

11.7.c Sense of self-esteem - Space for your comments

Showing 5 of 7 responses	
doesn't necessarily influence treatment but may affect their willingness/ability to change. often there are points of discussion that can help, but some may need further help from a Counsellor or similar.	298348-298340-27190466
see under diet and nutrition	298348-298340-26999243
depends if this is a symptom/ being treated	298348-298340-29496506
people I have found appear to be generally happier after acupuncture and feel very relaxed.	298348-298340-29668534
As above	298348-298340-29682718

11.8 Self-efficacy*

11.8.a Self-efficacy* - What you THINK NOW affects response

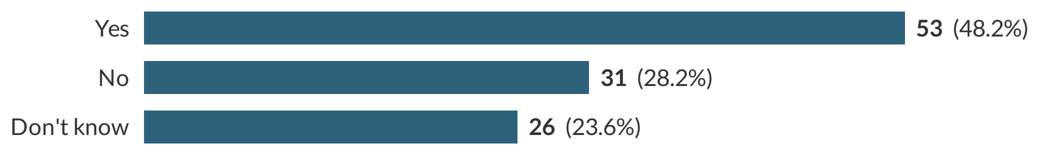

11.8.b Self-efficacy* - What you THOUGHT THEN (in clinic) affected response

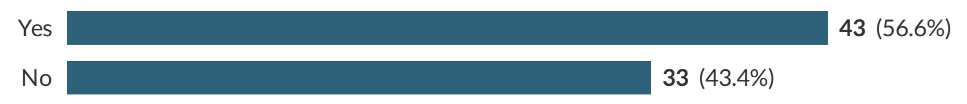

11.8.c Self-efficacy* - Space for your comments

Showing 5 of 6 responses	
what is this?	298348-298340-26488000
again something that we often measure before and after in Chronic Pain Clinics as it is a well known predictor for tendency to chronic pain.	298348-298340-27190466
see under diet and nutrition	298348-298340-26999243
Some patients respond well who do not appear to have high self efficacy.	298348-298340-29037670
depends if this is a symptom/ being treated	298348-298340-29496506

11.9 General sense of 'resilience'*

11.9.a General sense of 'resilience'* - What you THINK NOW affects response

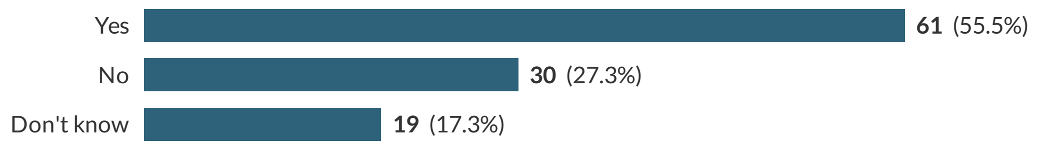

11.9.b General sense of 'resilience'* - What you THOUGHT THEN (in clinic) affected response

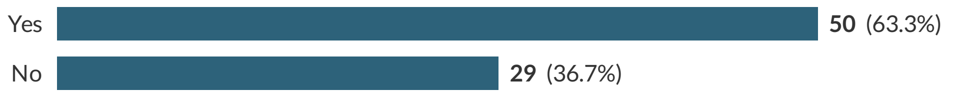

11.9.c General sense of 'resilience'* - Space for your comments

Showing all 3 responses	
see under diet and nutrition	298348-298340-26999243
depends if this is a symptom/ being treated	298348-298340-29496506
A positive trait that helps but does not impede the treatment.	298348-298340-30079001

11.10 Attachment style* ('secure' or not)

11.10.a Attachment style* ('secure' or not) - What you THINK NOW affects response

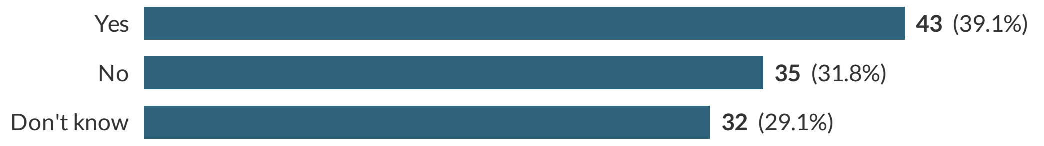

11.10.b Attachment style* ('secure' or not) - What you THOUGHT THEN (in clinic) affected response

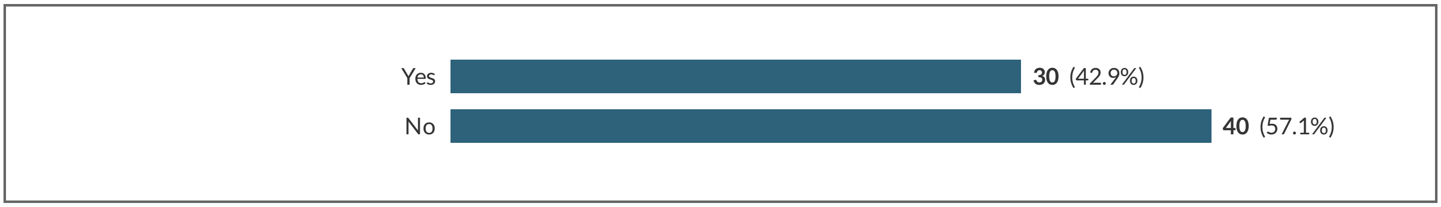

11.10.c Attachment style* (‘secure’ or not) - Space for your comments

Showing all 4 responses	
not necessarily a factor in outcome but may be important in the therapeutic relationship.	298348-298340-27190466
see under diet and nutrition	298348-298340-26999243
depends if this is a symptom/ being treated	298348-298340-29496506
Yes, may delay the response to treatment, for example people who are dependent emotionally on others. I would not distinguish any difference in the three attachment types.	298348-298340-30079001

11.11 Issues with gender identity or sexual orientation

11.11.a Issues with gender identity or sexual orientation - What you THINK NOW affects response

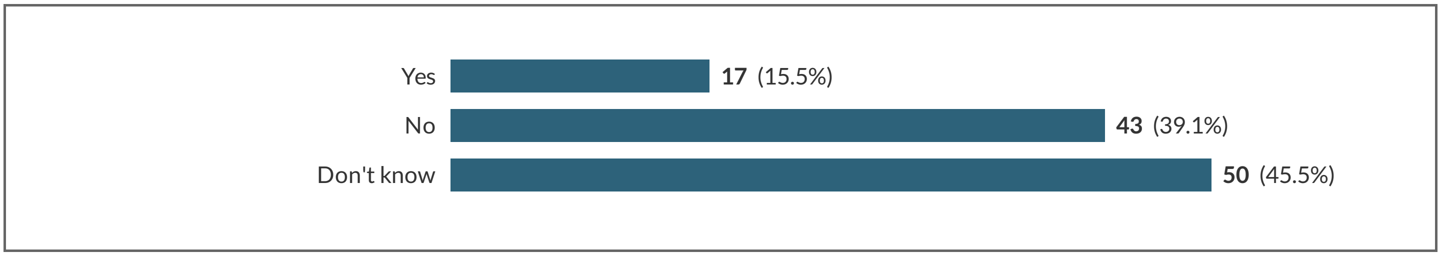

11.11.b Issues with gender identity or sexual orientation - What you THOUGHT THEN (in clinic) affected response

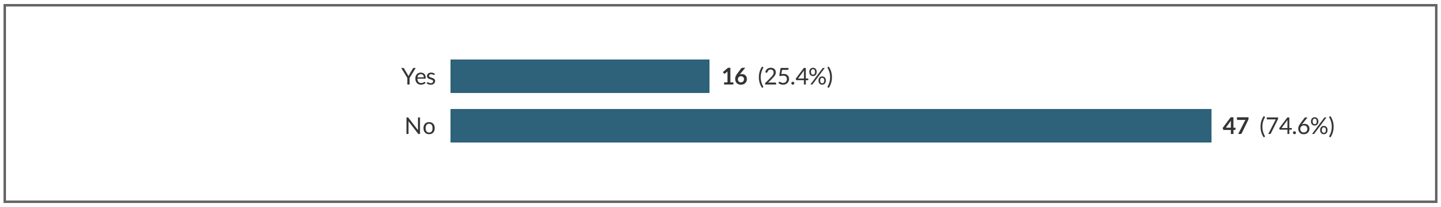

11.11.c Issues with gender identity or sexual orientation - Space for your comments

Showing 5 of 6 responses	
the patients we have seen in the Chronic Pain Clinic have had a lot of mental/emotional issues around this change, but then we don't get to see the ones who are coping well.	298348-298340-27190466
as a heterosexual male, being happy with who I am, I am uncertain how to answer this question or why and how it would influence my reaction to acupuncture	298348-298340-26999243
Haven't seen enough of thes people to comment	298348-298340-29297475
depends if this is a symptom/ being treated	298348-298340-29496506
Not enough experience to comment fairly	298348-298340-29749243

11.12 Optimism

11.12.a Optimism - What you THINK NOW affects response

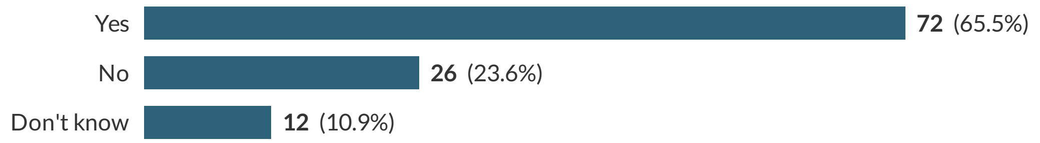

11.12.b Optimism - What you THOUGHT THEN (in clinic) affected response

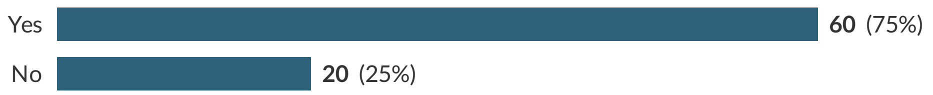

11.12.c Optimism - Space for your comments

Showing 5 of 7 responses	
Linked to treatment and post-treatment results perception. If patient believes nothing will change after the session, possibility that patient block flow of their Qi and are unable to relax.	298348-298340-26107614
see under diet and nutrition	298348-298340-26999243
I think if the therapist is optimistic this helps the patient.	298348-298340-29037670
The patients general outlook.	298348-298340-29668534
Some people believe the treatment will help, and for them it often does.	298348-298340-29682718

11.13 Emotion or mood most often positive (or negative)

11.13.a Emotion or mood most often positive (or negative) - What you THINK NOW affects response

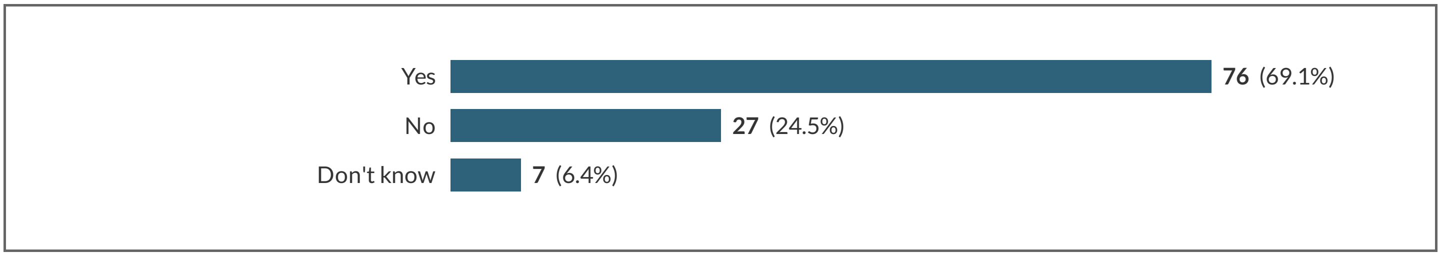

11.13.b Emotion or mood most often positive (or negative) - What you THOUGHT THEN (in clinic) affected response

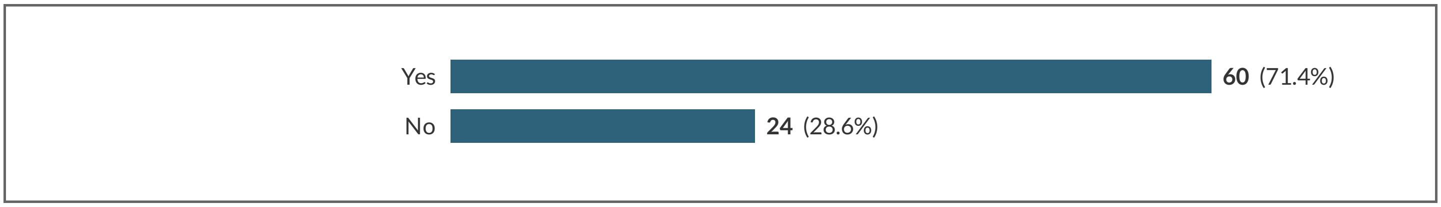

11.13.c Emotion or mood most often positive (or negative) - Space for your comments

**Showing 5 of 8 responses**	
Linked to treatment and post-treatment results perception. If patient believes nothing will change after the session, possibility that patient block flow of their Qi and are unable to relax.	298348-298340-26107614
none of the chronic pain patients would have treatment if this was true.	298348-298340-27190466
see under diet and nutrition	298348-298340-26999243
Emotion varies for each patient & each session.	298348-298340-29037670
Positive people seem to look for improvements, while other people don't seem to see them.	298348-298340-29228864

11.14 Emotional instability

11.14.a Emotional instability - What you THINK NOW affects response

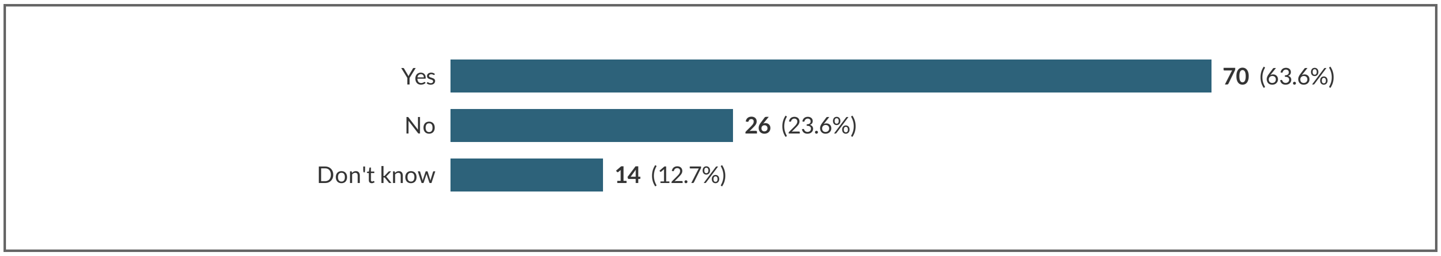

11.14.b Emotional instability - What you THOUGHT THEN (in clinic) affected response

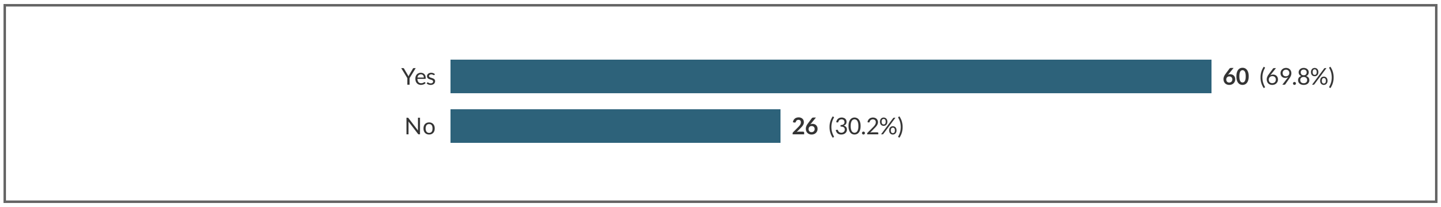

11.14.c Emotional instability - Space for your comments

Showing 5 of 7 responses	
again it's down to degrees. patients with a mental health disorder may not be able or willing to make any changes or be able to relax enough during the treatment.	298348-298340-27190466
see under diet and nutrition	298348-298340-26999243
Sometimes unstable people have trouble committing to treatment and stop too soon.	298348-298340-29228864
depends if this is a symptom/ being treated	298348-298340-29496506
Yes, it is like the wind and changes so can delay the response to treatment.	298348-298340-30079001

11.15 Self-regulation*

11.15.a Self-regulation* - What you THINK NOW affects response

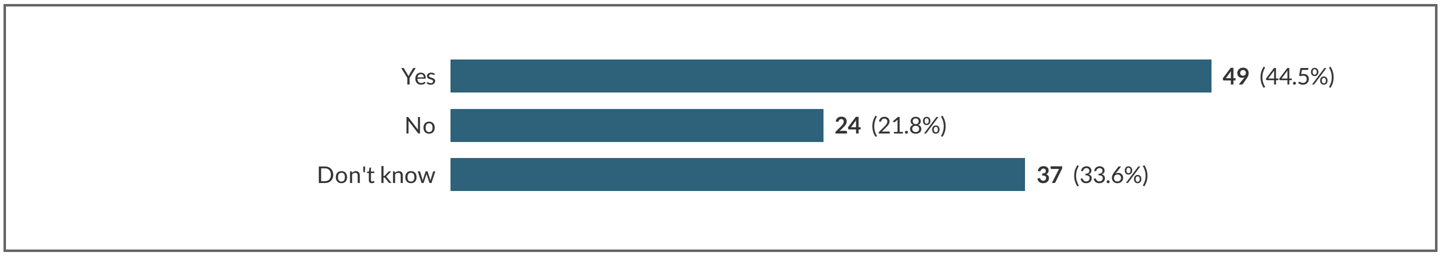

11.15.b Self-regulation* - What you THOUGHT THEN (in clinic) affected response

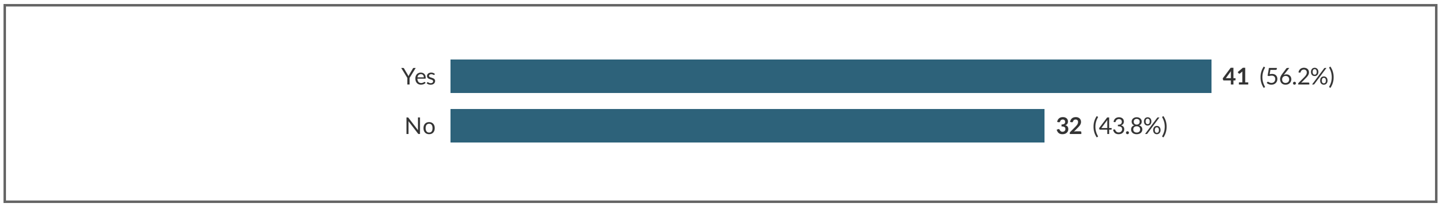

11.15.c Self-regulation* - Space for your comments

Showing all 3 responses	
see under diet and nutrition	298348-298340-26999243
Difficult to say, it depends if balanced.	298348-298340-30079001
Some people don't have this, it can make treatment outcomes slower	298348-298340-30475628

11.16 Sensitivity to stress

11.16.a Sensitivity to stress - What you THINK NOW affects response

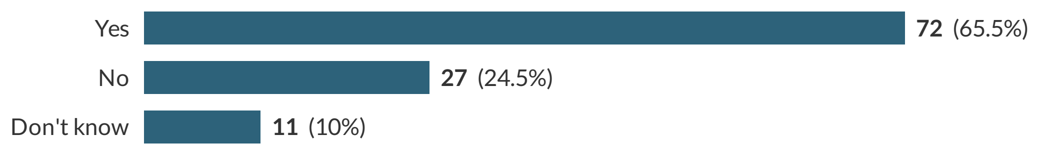

11.16.b Sensitivity to stress - What you THOUGHT THEN (in clinic) affected response

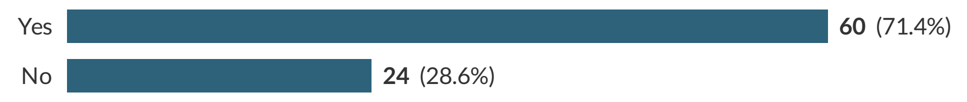

11.16.c Sensitivity to stress - Space for your comments

Showing all 4 responses	
see under diet and nutrition	298348-298340-26999243
depends if this is a symptom/ being treated	298348-298340-29496506
Immediate relief usual for these clients	298348-298340-29668534
Difficult to say, we are all sensitive but it depends on how we deal with stress.	298348-298340-30079001

11.17 Frequently or generally anxious

11.17.a Frequently or generally anxious - What you THINK NOW affects response

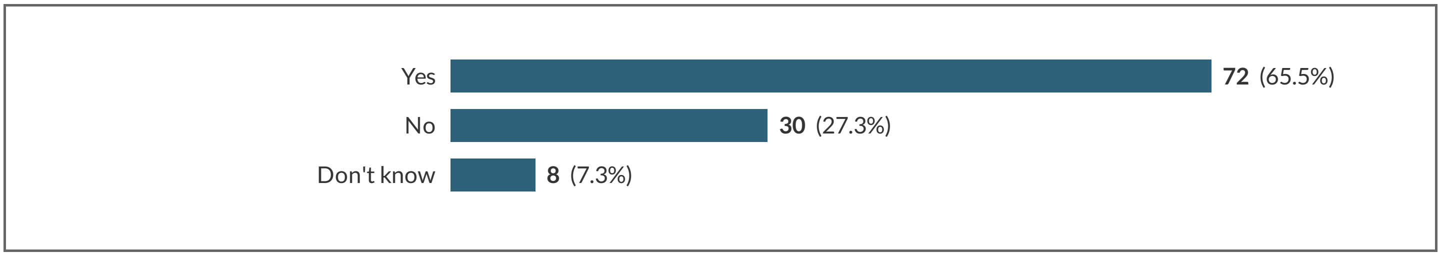

11.17.b Frequently or generally anxious - What you THOUGHT THEN (in clinic) affected response

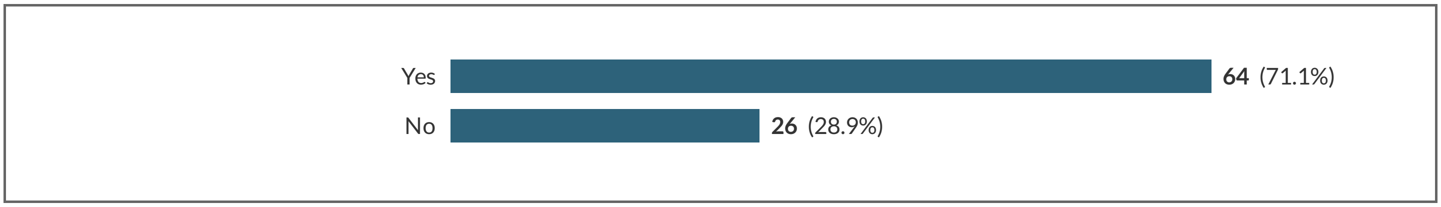

11.17.c Frequently or generally anxious - Space for your comments

Showing 5 of 8 responses	
someone with a proper anxiety disorder may be more difficult to help in the long term.	298348-298340-27190466
see under diet and nutrition	298348-298340-26999243
I think acupuncture can greatly help anxiety and if patients understand it will need a few treatments they will see results.	298348-298340-29037670
depends if this is a symptom/ being treated	298348-298340-29496506
Helps with anxiety sometimes results are realised a few hours after the treatment.	298348-298340-29668534

11.18 Depressive

11.18.a Depressive - What you THINK NOW affects response

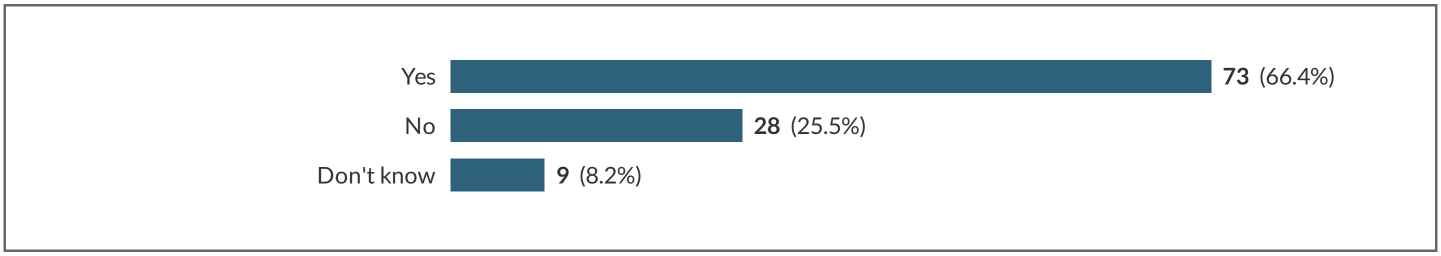

11.18.b Depressive - What you THOUGHT THEN (in clinic) affected response

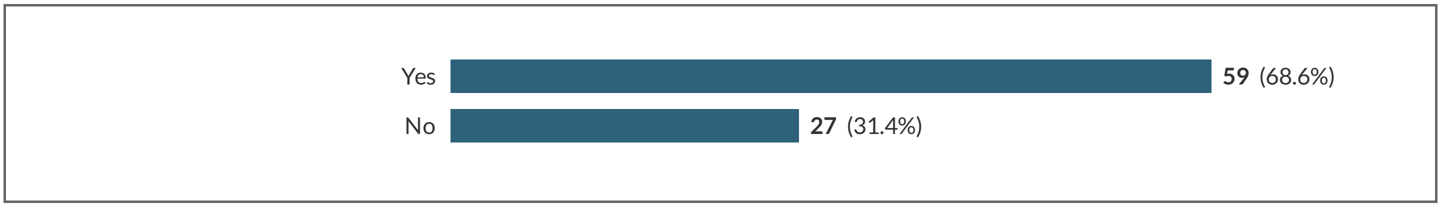

11.18.c Depressive - Space for your comments

Showing 5 of 10 responses	
Depends on degree	298348-298340-26488000
Depends on the cause of depression. If the patient has serious psychosis e.g. bipolar it can be difficult to treat.	298348-298340-26670536
have had people with bipolar disorder on treatment, once there stable on their meds.	298348-298340-27190466
see under diet and nutrition	298348-298340-26999243
I have patients who have had really good results with depressive symptoms.	298348-298340-29037670

11.19 Psychotic

11.19.a Psychotic - What you THINK NOW affects response

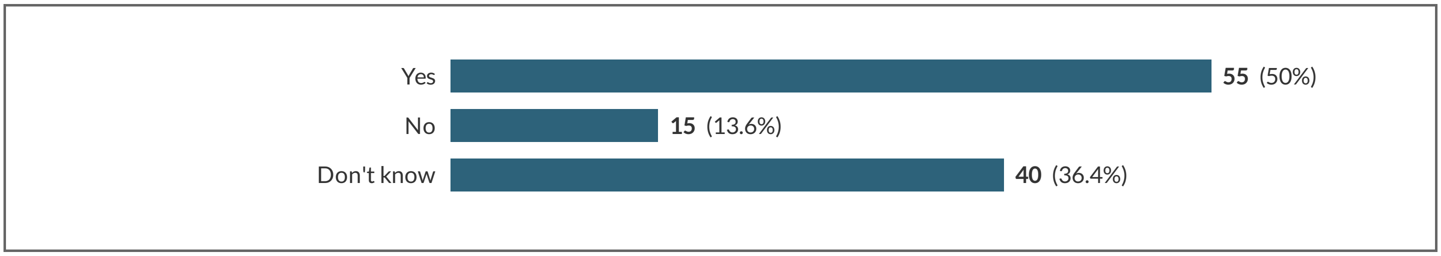

11.19.b Psychotic - What you THOUGHT THEN (in clinic) affected response

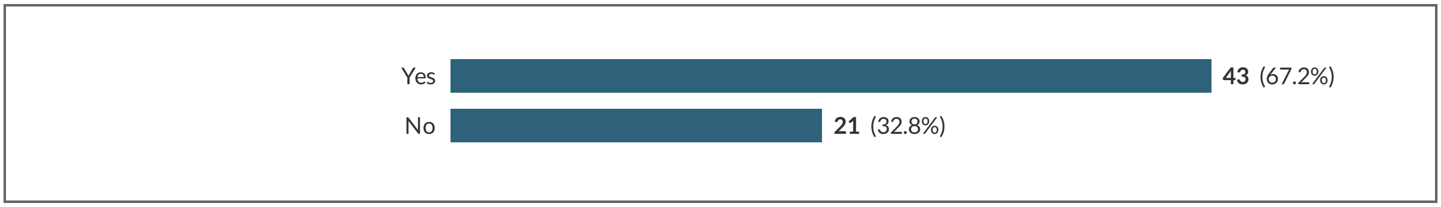

11.19.c Psychotic - Space for your comments

Showing 5 of 12 responses	
Generally unsuitable in psychosis	298348-298340-26488000
In serious cases of psychosis it can be very difficult to treat with acupuncture.	298348-298340-26670536
Not stable enough for treatment	298348-298340-26910831
Very little experience here and tend to guide very serious mental health to other professionals as I practice alone	298348-298340-26948161
would not treat someone with this disorder as too unpredictable. would need a special mental Health Unit to supervise.	298348-298340-27190466

11.20 Hypochondriacal

11.20.a Hypochondriacal - What you THINK NOW affects response

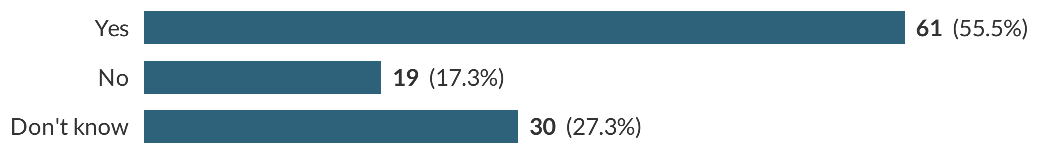

11.20.b Hypochondriacal - What you THOUGHT THEN (in clinic) affected response

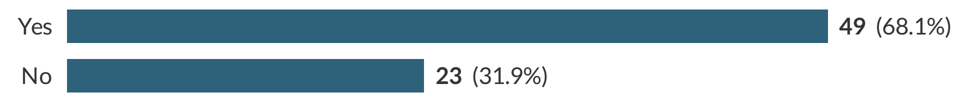

11.20.c Hypochondriacal - Space for your comments

Showing 5 of 9 responses	
Difficult to answer this	298348-298340-26488000
Patient with this issue was never satisfied and also reported feeling worse pain levels due to acupuncture treatment	298348-298340-26924099
Hypochondriacs may not want to get well	298348-298340-26971511
again depends on the degree. if they have a specific health anxiety, can be very difficult to change their beliefs in the long term.	298348-298340-27190466
see under diet and nutrition	298348-298340-26999243

11.21 Tending to magnify or catastrophise pain or other distress

11.21.a Tending to magnify or catastrophise pain or other distress - What you THINK NOW affects response

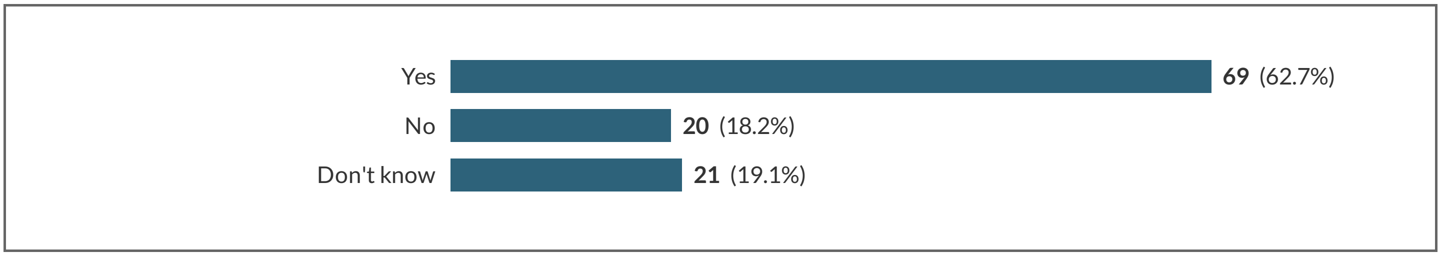

11.21.b Tending to magnify or catastrophise pain or other distress - What you THOUGHT THEN (in clinic) affected response

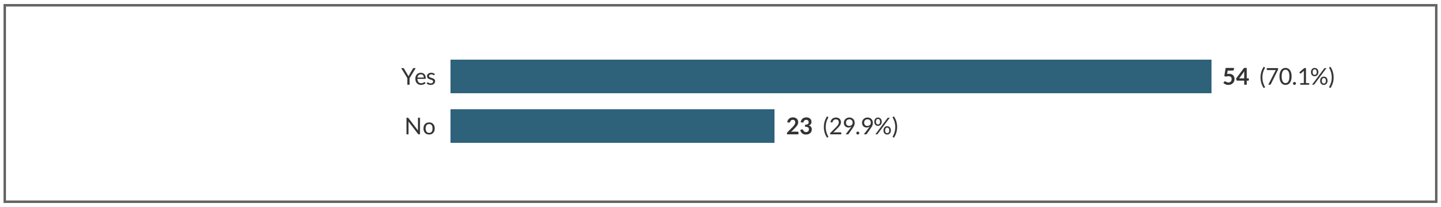

11.21.c Tending to magnify or catastrophise pain or other distress - Space for your comments

Showing all 5 responses	
One of my patients with this tendency seemed to not get much better, but when I questioned symptoms in detail there was definitely improvement. She seemed to almost found it hard to admit improvements and was anxious that she wouldn't be taken seriously if she admitted that she had less pain for example.	298348-298340-27059499
we deal with this a lot in the chronic pain unit and do a lot of work with them to help them understand what is actually going on and address their beliefs/concerns. by the time they have the acupuncture they're hopefully more open to change. if they're not we would probably get them more mental health input rather than acupuncture.	298348-298340-27190466
see under diet and nutrition	298348-298340-26999243
As above	298348-298340-29682718
Yes, usually who is very sensitive to pain has a slower response to treatment.	298348-298340-30079001

11.a Space for any additional comments you may wish to make:

Showing 5 of 13 responses	
Main cases I have seen for poor response are people who feel stuck in their lives and decide they cannot do anything about it. Response to treatment will decrease until very little response is observed. My understanding is that the emotions are trapped inside, leading to Qi stagnation BUT where the patient themselves consciously / unconsciously fight against the acupuncture treatment leading to an "energetic lockdown".	298348-298340-26107614
Difficult to answer some questions as a successful outcome depends at least 50% on how the therapist responds to the patient's difficulties which are not necessarily related to that particular visit.	298348-298340-26489299
I work in an NHS hospital where all of the patients I see have chronic health problems. Patients who do stuff to self-manage their condition tend to get more benefit from treatment where as those who don't do anything to self manage have more short term benefit. Patients who have problems with their mental health seem to be less able to make positive changes and therefore see less benefit	298348-298340-26546219
Some patients hold their illnesses very dearly to themselves and don't want to be without it. However they do value being listened to, touched, and cared for in some cases. Others are on a merry go round of trying loads of different therapies although they are not really serious about being better. Presumably the need to be seen as 'definitely ill' or 'incurable' helps them cope in their life at an unconscious level (so their minds would have them believe).	298348-298340-26920171
I work in a multidisciplinary team, where many patients have input from several heath professionals, all of whom have many years of experience in the field, all working together. Even so, there's a small percentage of patients that prove difficult to work with, despite our best efforts. This makes me feel that other practitioners that don't have that support and experience, could be vulnerable to being drawn in to a difficult situation or end up in a cycle of treatment going nowhere they can't get out of. Acupuncture can be a wonderful thing but it can't do the whole change on its own. if the patient is not correctly diagnosed from a Western perspective, especially MSK issues, then they won't be able to do what's needed to get their body in the best condition to help their health. also there are a lot of people with undiagnosed or untreated mental health issues that the patient does not recognise as being connected to their physical health problem and neither may the practitioner. We have long discussions with the Mental Health team about their referrals of patients to the Chronic Pain Clinic as to which needs to be dealt with1st. as most acupuncturists are sole practitioners who are unlikely to have the breadth of knowledge and experience in the team I work with, and so I feel that some people don't know what they don't know and could end up becoming dispirited from negative experiences. I hope this isn't the case but I suspect that it is.	298348-298340-27190466

12 Particular patient characteristics, attitudes or experience that you think may contribute to how well (or poorly) they respond to acupuncture. (Information on those that are asterisked can be found under 'More Info', above.)

12.1 Past surgery or other medical procedures considered ‘invasive’

12.1.a Past surgery or other medical procedures considered ‘invasive’ - What you THINK NOW affects response

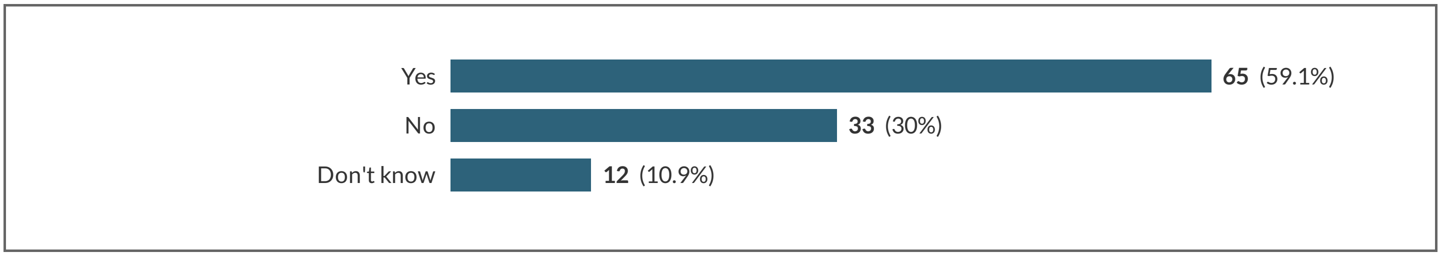

12.1.b Past surgery or other medical procedures considered 'invasive' - What you THOUGHT THEN (in clinic) affected response

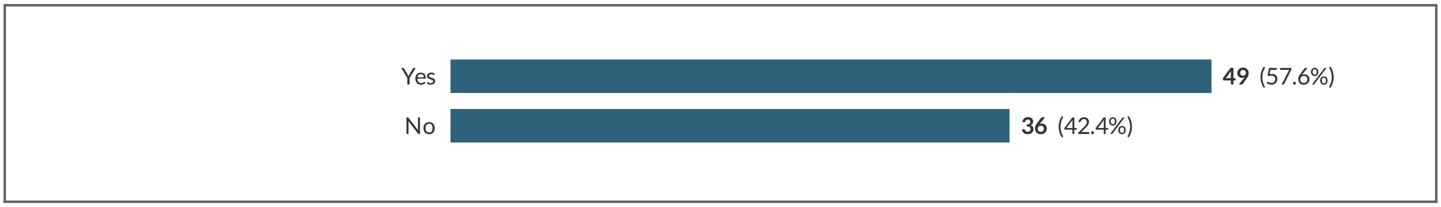

12.1.c Past surgery or other medical procedures considered 'invasive' - Space for your comments

Showing 5 of 9 responses	
In case of very significant structural damage e.g. bone tumor following surgery.	298348-298340-26107614
Depends on the surgery	298348-298340-26670536
Care needed with needle technique and choice of points	298348-298340-26951369
gastric banding/bypass -partly because of the problems thye have absorbing nutrient as a result of the surgery, partly because there are often a mental/emotional issues as a factor.	298348-298340-27190466
surgery and medical interventions alter the state of qi and hence the response to acupuncture	298348-298340-26999243

12.2 Previous 'doctor shopping'*

12.2.a Previous 'doctor shopping'* - What you THINK NOW affects response

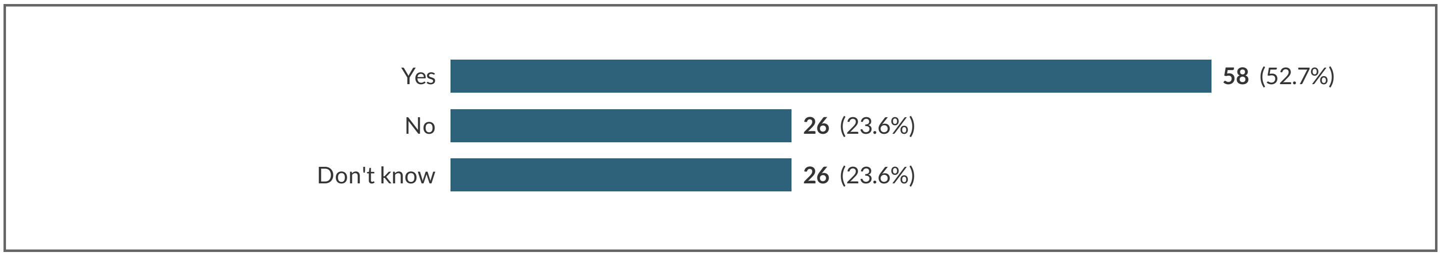

12.2.b Previous 'doctor shopping'* - What you THOUGHT THEN (in clinic) affected response

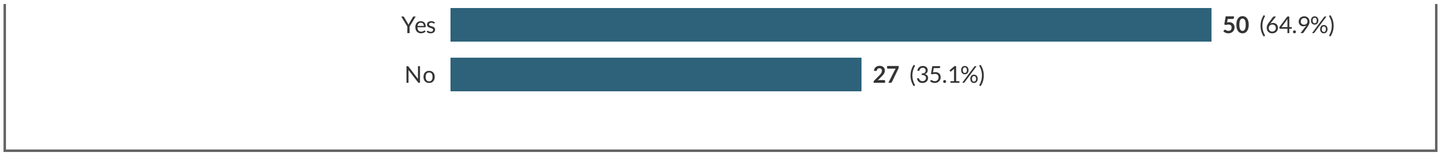

12.2.c Previous ‘doctor shopping’* - Space for your comments

Showing 5 of 10 responses	
Unrealistic expectations	298348-298340-26910831
Patient tended to shop around different professions seeking an answer to her problems while all the time knowing that the root of her issue was her addiction to sugar and psychological problems	298348-298340-26924099
May not really know what they are looking for and not positive about treatment.	298348-298340-26997929
we see this all the time in the Chronic Pain Clinic. doesn't preclude them from treatment, unless they persist in wanting a specific treatment.	298348-298340-27190466
'doctor shoppers' tend to be less responsive to anything other than the outcome they want. My guess is that their response to acupuncture is less than average	298348-298340-26999243

12.3 Multiple symptoms (co-morbidities) or somatisation

12.3.a Multiple symptoms (co-morbidities) or somatisation - What you THINK NOW affects response

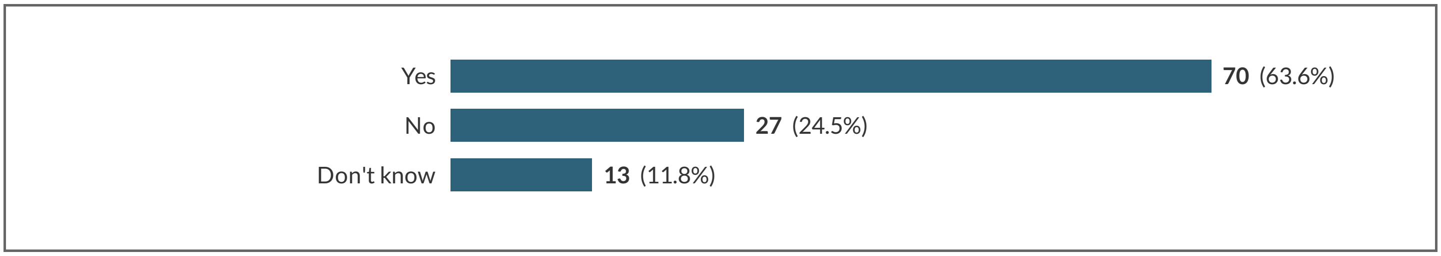

12.3.b Multiple symptoms (co-morbidities) or somatisation - What you THOUGHT THEN (in clinic) affected response

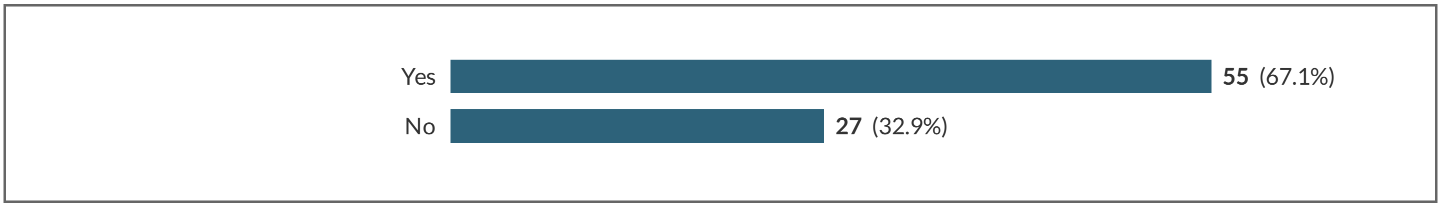

12.3.c Multiple symptoms (co-morbidities) or somatisation - Space for your comments

Showing 5 of 12 responses	
Takes some time to de-layer the pattern but the response is there.	298348-298340-26107614
It deoends what they are	298348-298340-26488000
most of my patients have complex health conditions and many co-morbidities and so it's often difficult to get long term benefitical results	298348-298340-26546219
Patient's who have several symptoms at once, sometimes come and add another so until practitioner delves deeper it's hard to see that old symptoms are improving, perhaps creating space for the patient to analyse new ones.	298348-298340-27066176
doesn't preclude treatment and some people who were considered poor responders do very well. However the more health problems someone ahs will affect their body's ability to respond.	298348-298340-27190466

12.4 Problems with addiction

12.4.a Problems with addiction - What you THINK NOW affects response

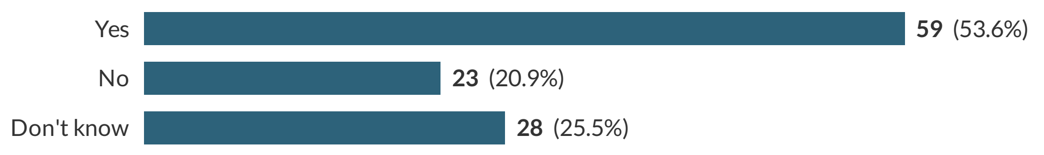

12.4.b Problems with addiction - What you THOUGHT THEN (in clinic) affected response

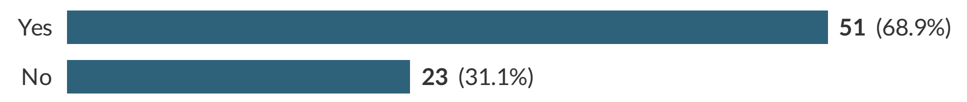

12.4.c Problems with addiction - Space for your comments

Showing 5 of 7 responses	
Response will be there if for a short time as addiction is an aggravating factor.	298348-298340-26107614
apart from the known effects on their physical health and often have associated mental health problems, the physiological effects on the brain can mean that their pain receptors and pain centres can no longer respond as they need to. even if they have been clean for along time, this damage can be irreversible	298348-298340-27190466
I find that their response to acupuncture is less	298348-298340-26999243
Yes as patients has come to realise that cravings are reduced they feel more relaxed and sleep better when they have acupuncture compared to when they do not attend clinic regularly.	298348-298340-29668534
Haven't met this situation.	298348-298340-29682718

12.5 Defensive*

12.5.a Defensive* - What you THINK NOW affects response

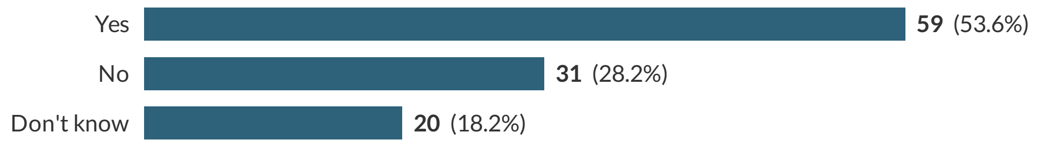

12.5.b Defensive* - What you THOUGHT THEN (in clinic) affected response

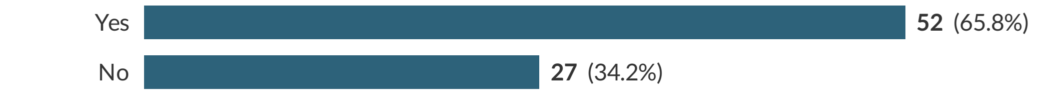

12.5.c Defensive* - Space for your comments

Showing all 5 responses	
Linked to treatment perception, could generate and energetic lockdown.	298348-298340-26107614
whether they are open to experience, or defensive, or any of the following circumstances will clearly effect, how they react to acupuncture	298348-298340-26999243
If the patient feels defensive, I would not treat as there is no thrust.	298348-298340-29682718
Yes, if experiencing treatment as an invasion.	298348-298340-30079001
Can make changes appear more slowly	298348-298340-30475628

12.6 Open to experience*

12.6.a Open to experience* - What you THINK NOW affects response

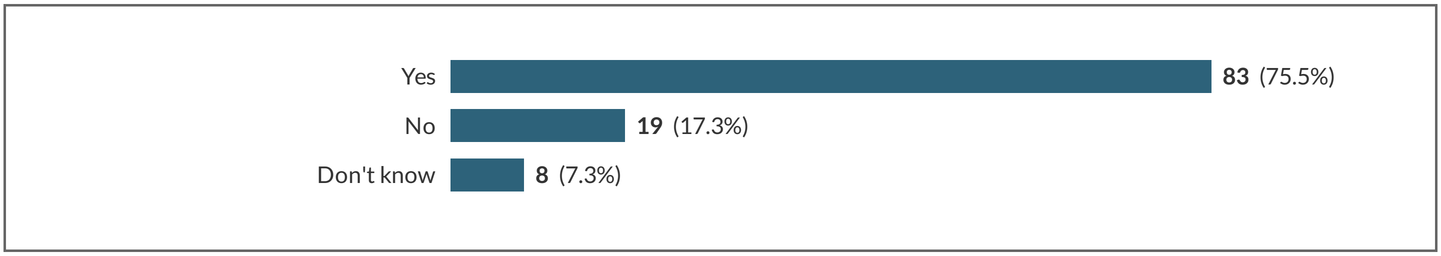

12.6.b Open to experience* - What you THOUGHT THEN (in clinic) affected response

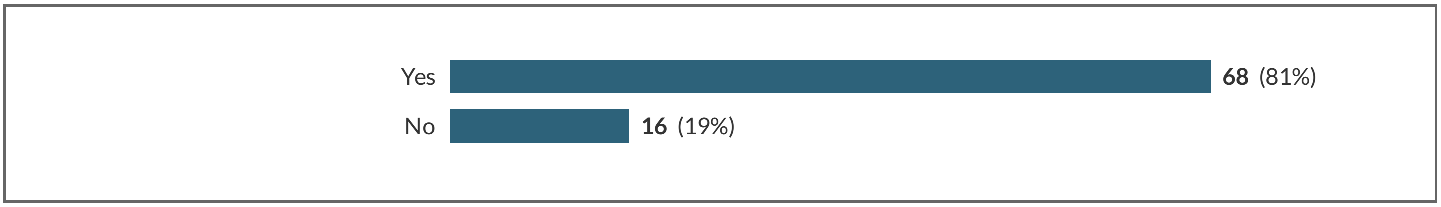

12.6.c Open to experience* - Space for your comments

Showing 5 of 7 responses	
Potentially greater response as patient is ready to embrace the flow of change.	298348-298340-26107614
Positive	298348-298340-26910831
ibid	298348-298340-26999243
Some people have an immediate response and therefore do not maintain treatments and return after a month when symptoms return.	298348-298340-29668534
Some patient just want to try to see if their back pain would ease. I would offer the the first treatment for free, for them to experience.	298348-298340-29682718

12.7 Suggestible

12.7.a Suggestible - What you THINK NOW affects response

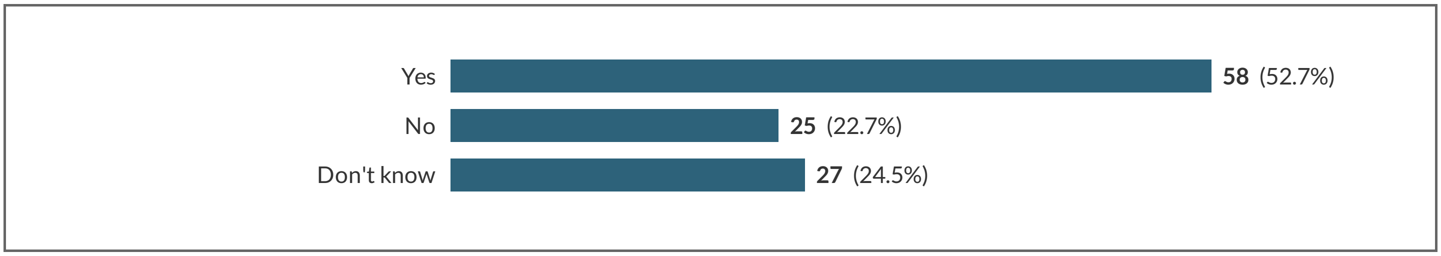

12.7.b Suggestible - What you THOUGHT THEN (in clinic) affected response

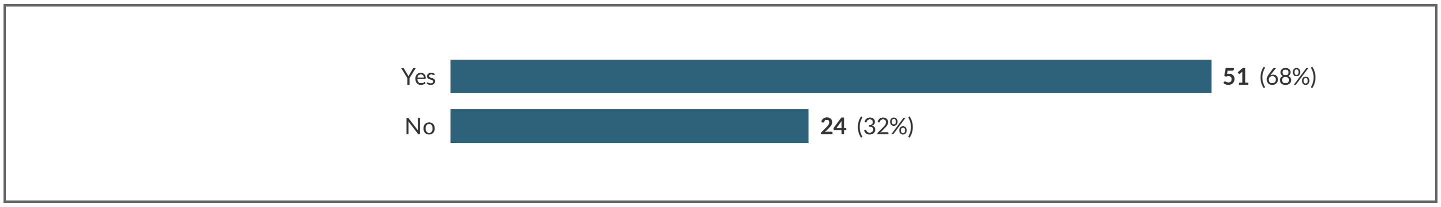

12.7.c Suggestible - Space for your comments

Showing all 3 responses	
Just better long term result if patient is ready to make some changes within their lifestyle.	298348-298340-26107614
ibid	298348-298340-26999243
Do not understand the implication.	298348-298340-30079001

12.8 Sceptical

12.8.a Sceptical - What you THINK NOW affects response

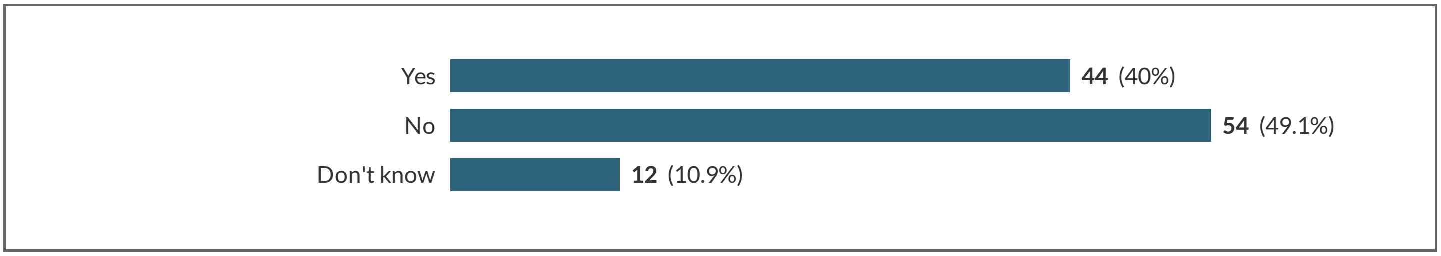

12.8.b Sceptical - What you THOUGHT THEN (in clinic) affected response

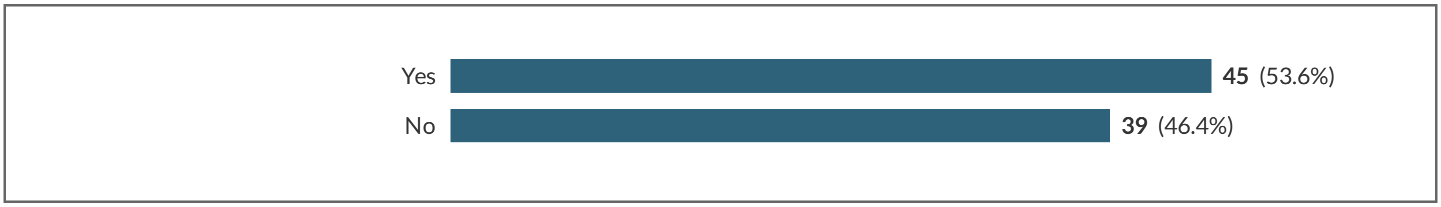

12.8.c Sceptical - Space for your comments

Showing 5 of 11 responses	
Scepticism about acupuncture makes no difference	298348-298340-26488000
DNA acupuncture removes pain immediately, this removes skepticism	298348-298340-26924099
ibid	298348-298340-26999243
Sceptical patients- great when treatments go well with good response as they can't deny it. However they will always play it down. Saying they never feel any different after treatment- but also saying symptoms have improved!	298348-298340-29037670
A lot of people are sceptical at first.	298348-298340-29228864

12.9 Placebo responsive*

12.9.a Placebo responsive* - What you THINK NOW affects response

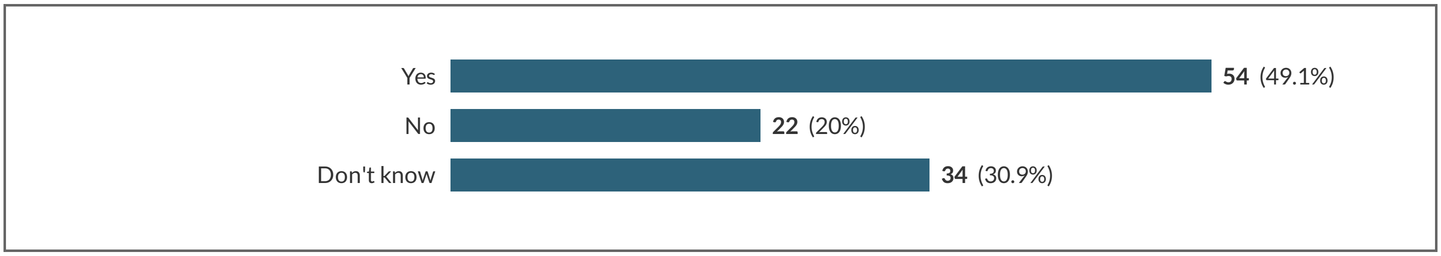

12.9.b Placebo responsive* - What you THOUGHT THEN (in clinic) affected response

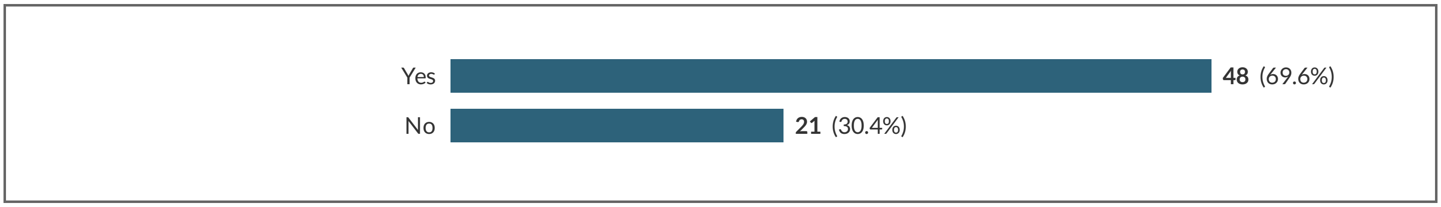

12.9.c Placebo responsive* - Space for your comments

Showing 5 of 8 responses	
Obviously a central issue in acupuncture!	298348-298340-26488000
ibid	298348-298340-26999243
what?	298348-298340-29496506
Some people may be responsive to this situation.	298348-298340-29682718
Yes but only in the very short term.	298348-298340-29749243

12.10 Able to trust others

12.10.a Able to trust others - What you THINK NOW affects response

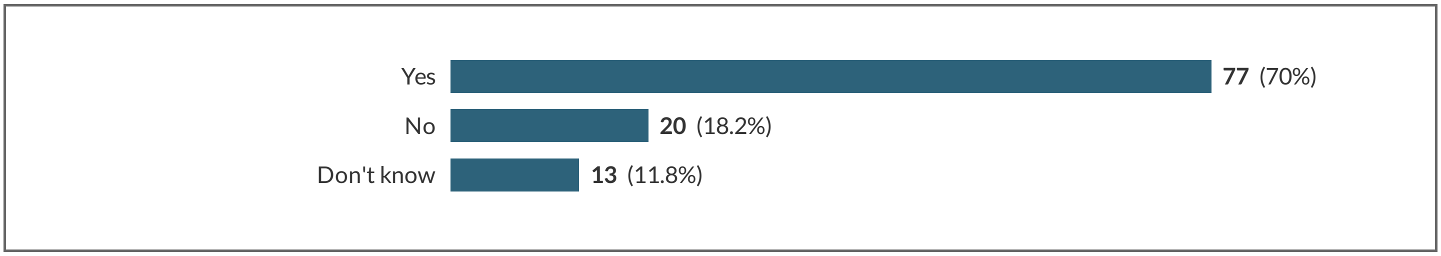

12.10.b Able to trust others - What you THOUGHT THEN (in clinic) affected response

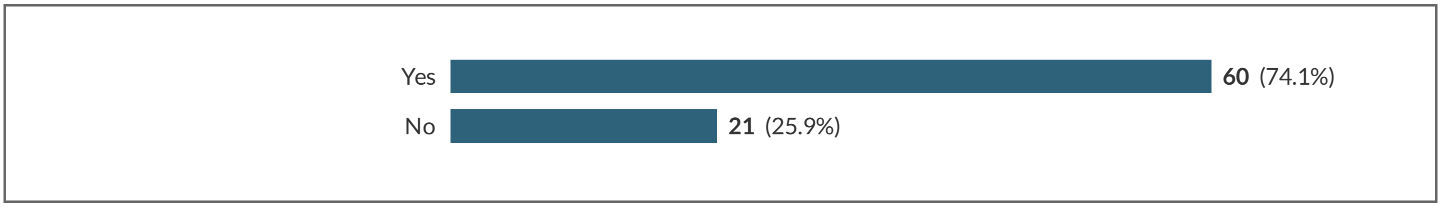

12.10.c Able to trust others - Space for your comments

Showing 5 of 7 responses	
Linked to treatment perception, could generate and energetic lockdown if open distrust.	298348-298340-26107614
Not being open about symptoms and outcome.	298348-298340-26670536
establishing trust is generally key to successful treatment, sometimes it takes longer to achieve for some patients	298348-298340-26948161
ibid	298348-298340-26999243
but needed to have any threatment	298348-298340-29496506

12.11 Willing to follow advice or instructions

12.11.a Willing to follow advice or instructions - What you THINK NOW affects response

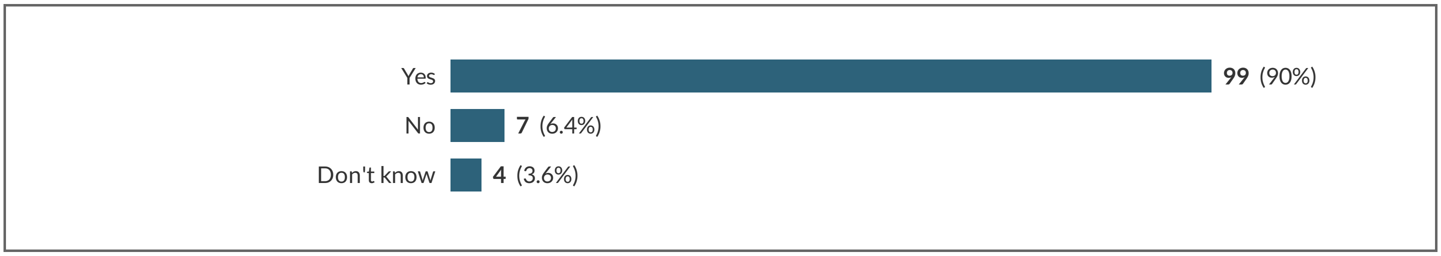

12.11.b Willing to follow advice or instructions - What you THOUGHT THEN (in clinic) affected response

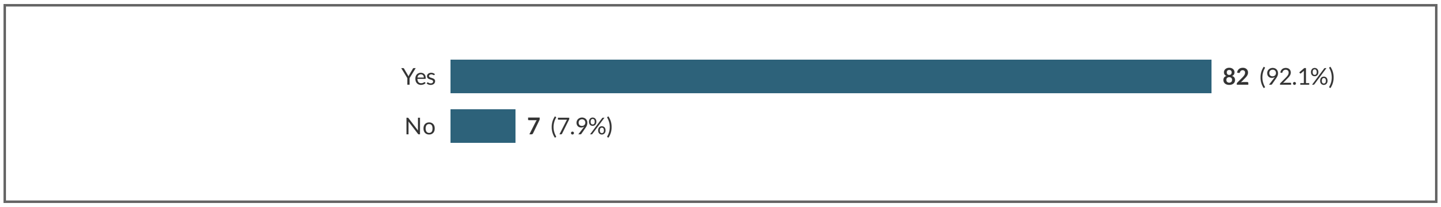

12.11.c Willing to follow advice or instructions - Space for your comments

Showing 5 of 8 responses	
Just better long term result if patient is ready to make some changes within their lifestyle.	298348-298340-26107614
We are only part of the healing, engaged motivated patients do well	298348-298340-26948161
ibid	298348-298340-26999243
give right level of advice	298348-298340-29496506
More dietary advice.	298348-298340-29668534

12.12 Committed to long-term treatment

12.12.a Committed to long-term treatment - What you THINK NOW affects response

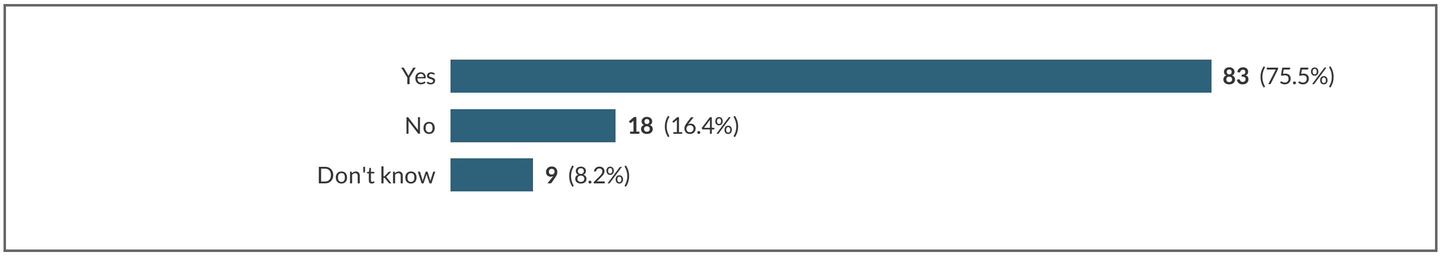

12.12.b Committed to long-term treatment - What you THOUGHT THEN (in clinic) affected response

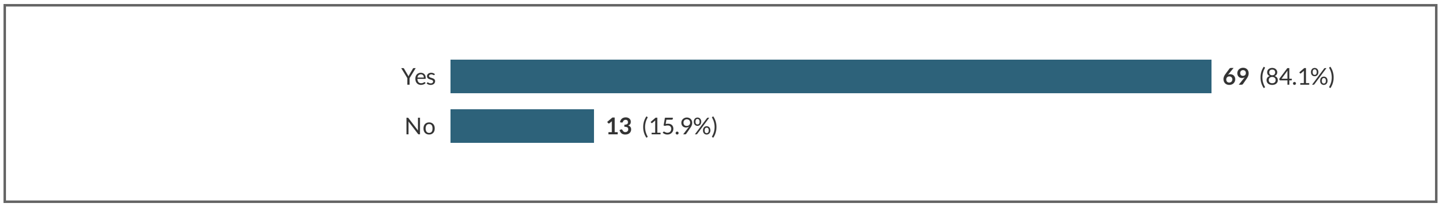

12.12.c Committed to long-term treatment - Space for your comments

Showing 5 of 10 responses	
Most times, greatest response comes from first treatment as patient's Qi and emotions have been restrained for some time.	298348-298340-26107614
Not normally relevant - can't predict this initially	298348-298340-26488000
I always give patients the control to come back 'when they feel they are ready for another treatment' as opposed to a forced set of treatments - one a week for the next 8 weeks....	298348-298340-26489299
unfortunately some people are committed to long term treatment without wanting to change and may use it as an excuse not to change.	298348-298340-27190466
ibid	298348-298340-26999243

12.13 Tending to arousal (extraverted) or avoidance (introverted)

12.13.a Tending to arousal (extraverted) or avoidance (introverted) - What you THINK NOW affects response

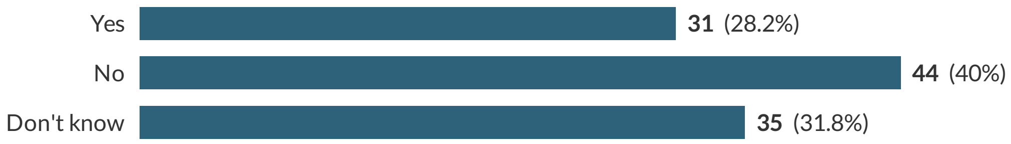

12.13.b Tending to arousal (extraverted) or avoidance (introverted) - What you THOUGHT THEN (in clinic) affected response

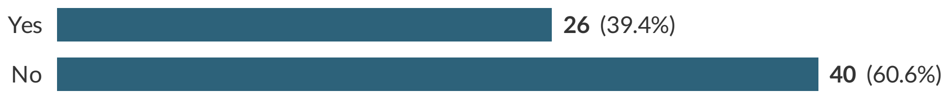

12.13.c Tending to arousal (extraverted) or avoidance (introverted) - Space for your comments

Showing 1 response	
ibid	298348-298340-26999243

12.14 Bodily aware or sensitive

12.14.a Bodily aware or sensitive - What you THINK NOW affects response

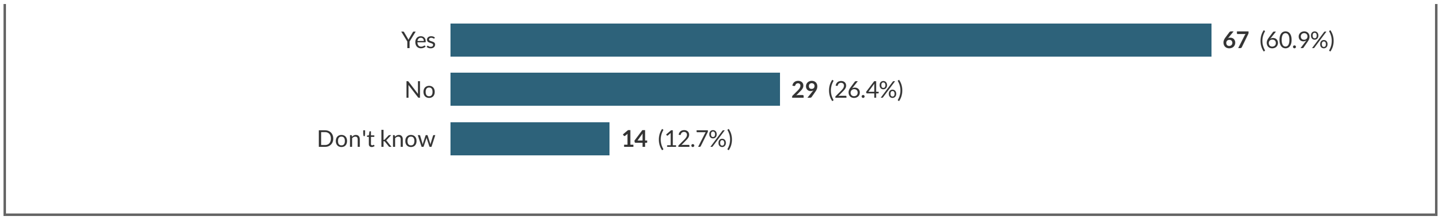

12.14.b Bodily aware or sensitive - What you THOUGHT THEN (in clinic) affected response

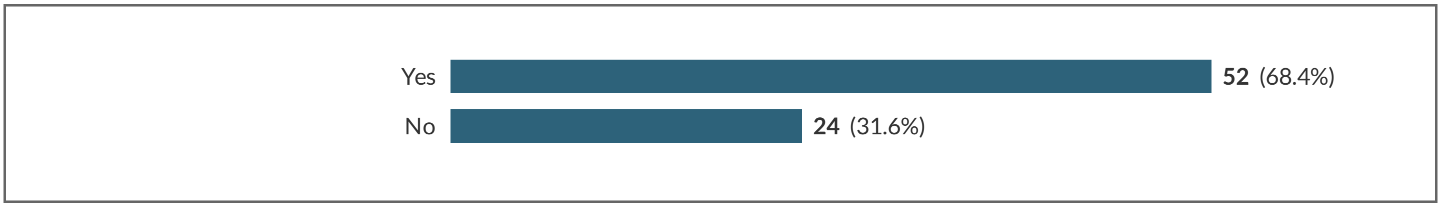

12.14.c Bodily aware or sensitive - Space for your comments

Showing 5 of 6 responses	
You can get patients who don’t want you to needle in certain places that can affect treatment	298348-298340-26997929
ibid	298348-298340-26999243
Better able to communicate the changes rather than better responders	298348-298340-28415546
Regular attendees develop sensitivity to needles on some occasions.	298348-298340-29668534
Yoga teachers I find very sensitive and describe energy moving around their bodies with acupuncture	298348-298340-29749243

12.15 Emotionally aware or sensitive

12.15.a Emotionally aware or sensitive - What you THINK NOW affects response

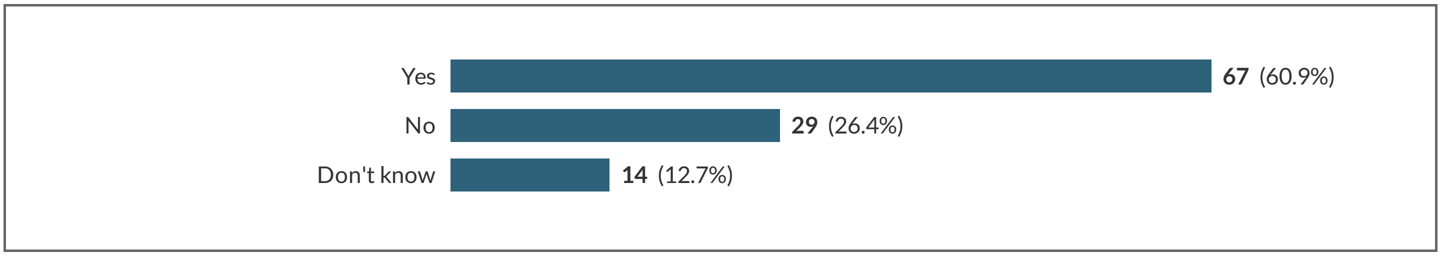

12.15.b Emotionally aware or sensitive - What you THOUGHT THEN (in clinic) affected response

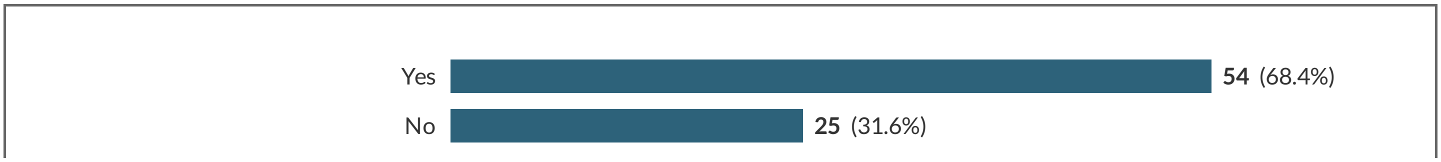

12.15.c Emotionally aware or sensitive - Space for your comments

Showing all 4 responses	
ibid	298348-298340-26999243
Yes - because these patients are already aware how their emotional health affects the rest of them and are able to express themselves extremely honestly and I think this helps sometimes with finding the root of a problem and therefore relief from the symptom.	298348-298340-29749243
Yes, it helps but it this can also improve with the treatment.	298348-298340-30079001
Helps	298348-298340-30475628

12.16 Alexithymic*

12.16.a Alexithymic* - What you THINK NOW affects response

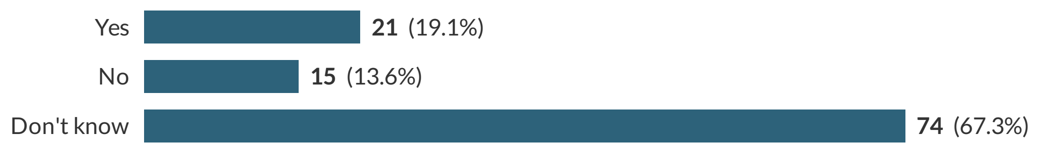

12.16.b Alexithymic* - What you THOUGHT THEN (in clinic) affected response

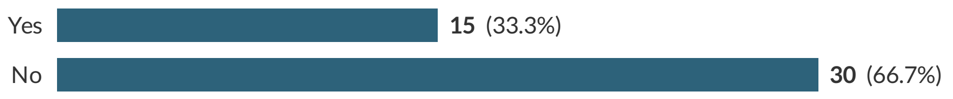

12.16.c Alexithymic* - Space for your comments

Showing 5 of 6 responses	
Very difficult to tell if a patient is alexithymic - can only respond to how the patient presents - nonjudgmental.	298348-298340-26489299
I find trust is a key component in effective treatment	298348-298340-26948161
ibid	298348-298340-26999243
Only because I'm really not sure what this means despite reading the explanation...	298348-298340-29749243
Not sure what this means. Feelings are by definition 'bodily sensations' (am I Alexithymic, if so, how would I know)	298348-298340-29870129

12.17 Qi strong or weak

12.17.a Qi strong or weak - What you THINK NOW affects response

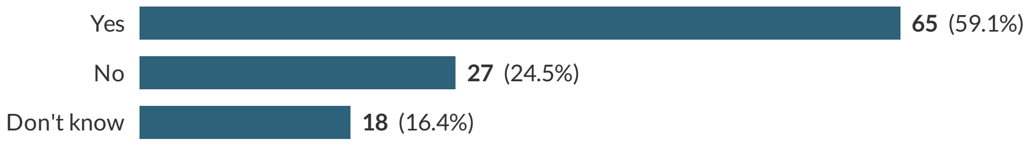

12.17.b Qi strong or weak - What you THOUGHT THEN (in clinic) affected response

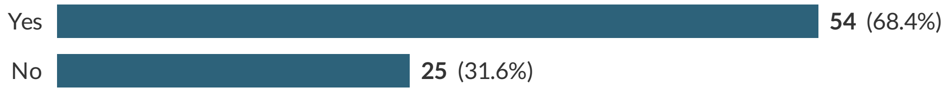

12.17.c Qi strong or weak - Space for your comments

Showing 5 of 13 responses	
Same as comments for Jing deficiency. However there will be some good response as the Qi is built up, generally around the 2nd or 3rd treatment.	298348-298340-26107614
Don't use this terminology	298348-298340-26488000
Some patients are not so aware. You can only try to illicit deqi for a short time for some patients as they can become distressed. Treatments work even when deqi is not felt. Patients who seem to have strong qi sometimes have lack of sensitivity.	298348-298340-26489299
Weak pulses show deficient conditions which take longer to get beneficial results from	298348-298340-26546219
There usually is some sort of improvement.Whatever the state of Qi	298348-298340-26670536

12.18 Particular TCM/5E diagnostic patterns (please specify)

12.18.a Particular TCM/5E diagnostic patterns (please specify) - What you THINK NOW affects response

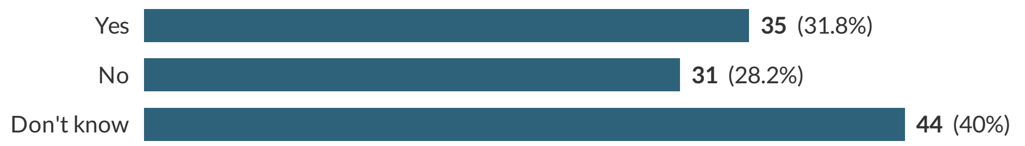

12.18.b Particular TCM/5E diagnostic patterns (please specify) - What you THOUGHT THEN (in clinic) affected response

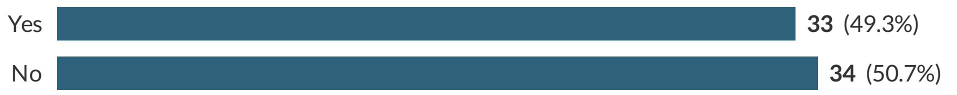

12.18.c Particular TCM/5E diagnostic patterns (please specify) - Space for your comments

Showing 5 of 32 responses	
LI channel especially	298348-298340-26471588
Chronic complaints with deep emotional roots are difficult to treat.	298348-298340-26471554
Some conditions get better responses than others. For example I would expect to get better results for someone with liver qi stagnation over kidney yin deficiency	298348-298340-26546219
HW or ID/Eds can be amazingly effective	298348-298340-26670536
I follow certain patterns and ‘recipes’ adding in various points when necessary	298348-298340-26786544

12.a Space for any additional comments you may wish to make:

Showing 5 of 6 responses	
.	298348-298340-26488000
the answer to you questions seem obvious to me. I am amazed, if not bewildered, that anyone thought that that would not be so	298348-298340-26999243
All factors of all people affect the outcome of acupuncture.	298348-298340-29225890
I find that some patients may be sceptical or alexithymic for example but with treatment this changes	298348-298340-29743878
Pointless	298348-298340-29782088

13 Do you think that some patients respond consistently well or poorly to acupuncture, almost regardless of other factors such as the condition treated, their state of health at the time, etc.?

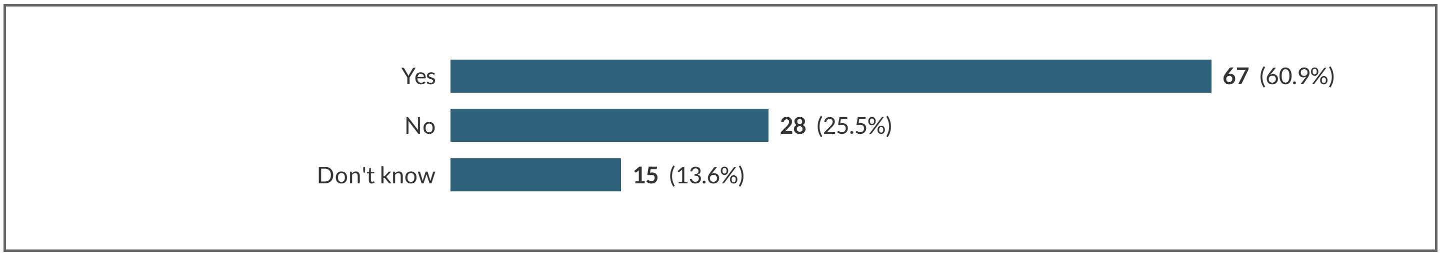

14 Do you find that an immediate response to treatment (e.g. a change in relaxation or a sense of wellbeing) is likely to indicate that a patient will experience longer-term changes as well?

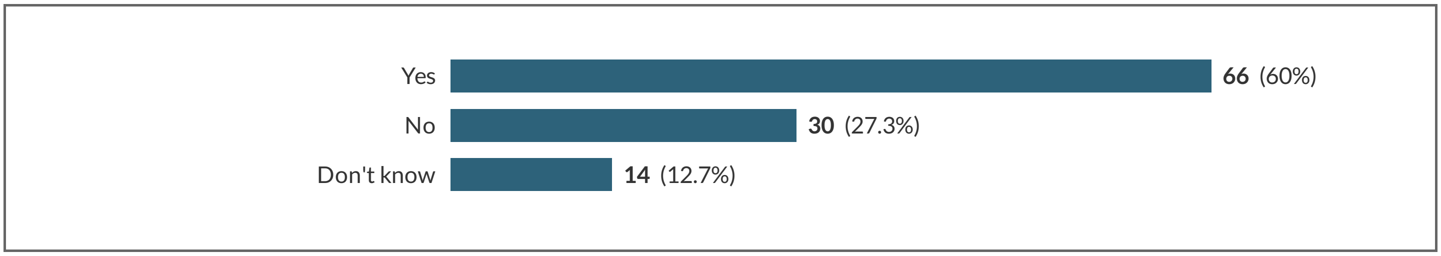

15 Have you had acupuncture treatment yourself?

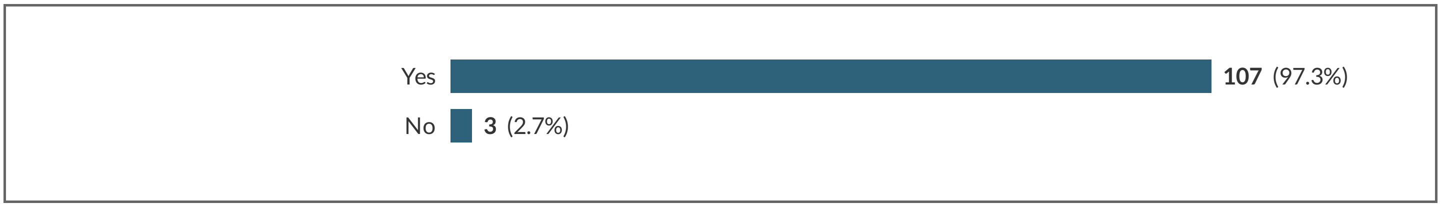

15.a If you answered 'Yes' to Q 15, would you describe yourself as:

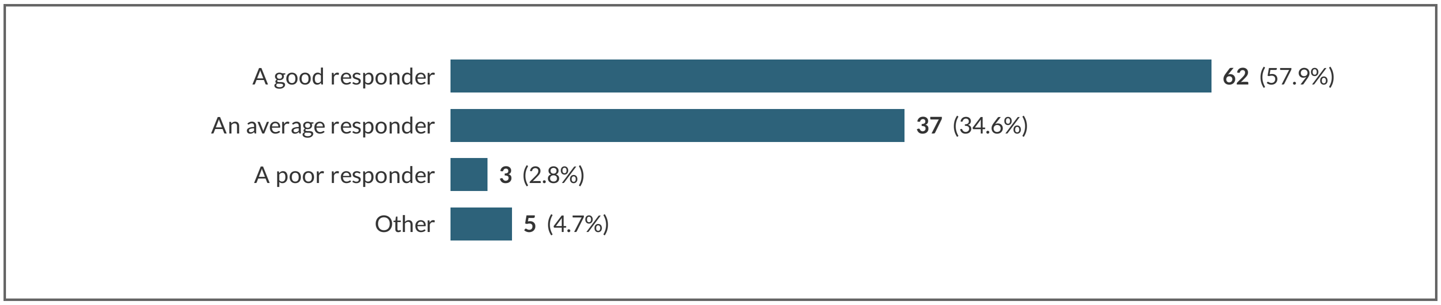

15.a.i Do you have any thoughts about why you respond as you do? (in your own words)

Showing 5 of 82 responses	
I have only acupuncture treatment for minor conditions, so response is good but not life-changing as I care well for myself with regular Nei-gong / Qi-gong and physical exercise. Also, I mainly treat myself, which is usually different when getting treated by someone else because of the difference in energy frequency (stronger signal & response with energy / qi different than yourself).	298348-298340-26107614
No	298348-298340-26471588
Expectation that it will be effective!	298348-298340-26472615
Just the way I'm built, presumably.	298348-298340-26488000
Too analytical about the treatment given and very critical of the outcome. Usually see it as a learning experience.	298348-298340-26489299

15.a.ii If you selected 'Other', please specify.

Showing all 5 responses	
Point choice and insertion, location techniques	298348-298340-26951369
I am a slow responder- it may take several days before I notice a difference and the changes are gradual	298348-298340-28415546
It depends on the practitioner and the quality of their diagnosis and treatment	298348-298340-29743878
I have no way of knowing	298348-298340-29782088
See above plus I don't always respond the same way every time.	298348-298340-29870129

16 What percentage of your patients would you describe as good responders to acupuncture?

16.1 0% vs 100%

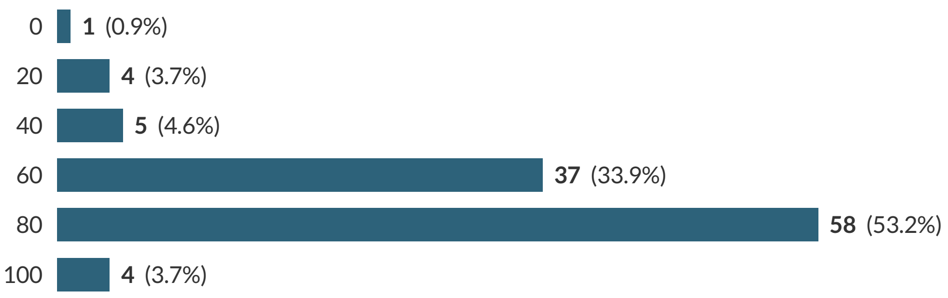

17 What percentage of your patients would you describe as poor responders to acupuncture?

17.1 0% vs 100%

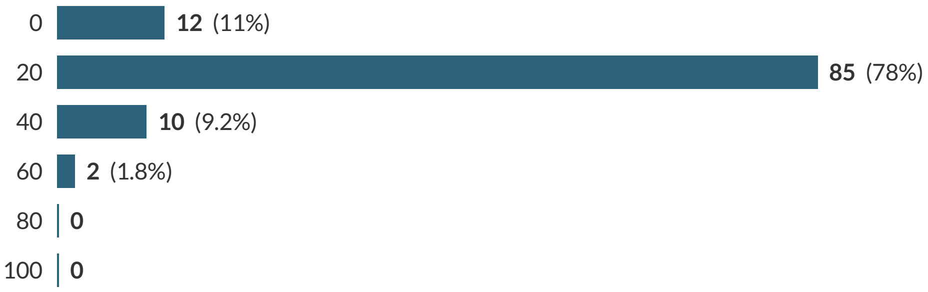

18 In your experience, have you found any conditions that respond particularly well to acupuncture? If so, please give some examples here:

**Showing 5 of 103 responses**	
IBS, Fybromyalgia, Stress, Anxiety, Asthma, PMS, Musculoskeletal disorders, stroke, Infertility, light mental disorders / emotional imbalance.	298348-298340-26107614
Emotional symptoms and MSK conditions	298348-298340-26269325
OA knee<br>non-specific and radiating LBP<br>frozen shoulder<br>tendonopathy<br>neuropathic pain	298348-298340-26471564
Wry neck<br>Achilles tendonitis<br>Tennis elbow	298348-298340-26471588
Anxiety	298348-298340-26471554

19 In your experience, have you found any conditions that respond particularly poorly to acupuncture? If so, give please some examples here:

Showing 5 of 99 responses	
Bloating, when patient carries on with poor lifestyle and does not care about aggravating factors. Emotions, when patient refuses to take the time to deep dive inside their heads and remain blocked in their current pattern of dissatisfaction with their lives.	298348-298340-26107614
autoimmune conditions	298348-298340-26269325
smoking cessation	298348-298340-26471588
Chronic pain with multiple problems	298348-298340-26471554
Post-herpetic neuralgia of chest or abdomen.<br>Deafness	298348-298340-26472615

20 Space for any further comments you may have:

Showing 5 of 37 responses	
Thank you for creating this survey, truly interesting and looking forward to seeing the results.	298348-298340-26107614
As with the problem of short-term response without longer benefit, the difficulty of good v poor responders may be due to patient characteristics, but is actually more likely to be due to either poor/good interaction with the acupuncturist or simply the wrong method of treatment with inappropriate points used or type of stimulation.	298348-298340-26472615
Response strength is continuously variable. It's a matter of degree, not all or nothing, so percentages are fairly meaningless.<br><br>I recognise a group I call pseudo-strong reactors. These react strongly the first time, but thereafter nothing happens. Curiously, these people often believe strongly that acupuncture is going to work. The best results are often in unimaginative middle-aged men who tell you afterwards that they didn't expect it to work!	298348-298340-26488000
I think there is an area of maintenance which isn't covered. I have many patients with, for example, work related pain - lifting etc - and they come maybe monthly for an MOT as they call it. Acupuncture is invaluable to them but can't be said to cure their problems.	298348-298340-26489299
Red heads respond well and may have an "over response"	298348-298340-26626426
